# When Are Statins Cost-Effective in Cardiovascular Prevention? A Systematic Review of Sponsorship Bias and Conclusions in Economic Evaluations of Statins

**DOI:** 10.1371/journal.pone.0069462

**Published:** 2013-07-08

**Authors:** Ferrán Catalá-López, Gabriel Sanfélix-Gimeno, Manuel Ridao, Salvador Peiró

**Affiliations:** 1 Centro Superior de Investigación en Salud Pública (CSISP-FISABIO), Red de Investigación en Servicios de Salud en Enfermedades Crónicas (REDISSEC), Valencia, Spain; 2 Instituto Aragonés de Ciencias de la Salud (I+CS), Red de Investigación en Servicios de Salud en Enfermedades Crónicas (REDISSEC), Zaragoza, Spain; University of California, San Francisco, United States of America

## Abstract

**Background:**

We examined sponsorship of published cost-effectiveness analyses of statin use for cardiovascular (CV) prevention, and determined whether the funding source is associated with study conclusions.

**Methods and Findings:**

We searched PubMed/MEDLINE (up to June 2011) to identify cost-effectiveness analyses of statin use for CV prevention reporting outcomes as incremental costs per quality-adjusted life years (QALY) and/or life years gained (LYG). We examined relationships between the funding source and the study conclusions by means of tests of differences between proportions. Seventy-five studies were included. Forty-eight studies (64.0%) were industry-sponsored. Fifty-two (69.3%) articles compared statins versus non-active alternatives. Secondary CV prevention represented 42.7% of articles, followed by primary CV prevention (38.7%) and both (18.7%). Overall, industry-sponsored studies were much less likely to report unfavourable or neutral conclusions (0% versus 37.1%; *p*<0.001). For primary CV prevention, the proportion with unfavourable or neutral conclusions was 0% for industry-sponsored studies versus 57.9% for non-sponsored studies (*p*<0.001). Conversely, no statistically significant differences were identified for studies evaluating secondary CV prevention (0% versus 12.5%; *p*=0.222). Incremental costs per QALY/LYG estimates reported in industry-sponsored studies were generally more likely to fall below a hypothetical willingness-to-pay threshold of US $50,000.

**Conclusions:**

Our systematic analysis suggests that pharmaceutical industry sponsored economic evaluations of statins have generally favored the cost-effectiveness profile of their products particularly in primary CV prevention.

## Introduction

The recognition that health services, like other activities, have limited resources has lead to the widespread adoption of cost-effectiveness criteria, a collection of methods intended to help decision-makers make the best choices for the adoption of or disinvestment in health technologies under conditions of uncertainty, conflicting objectives and resource constraints [1]. Healthcare decisions and policies are being increasingly driven by cost-effectiveness analyses (CEA), and in several countries the incremental cost-effectiveness ratio (ICER) has become a key determinant of whether a health technology will be publicly funded [2]. Thus, a large number of economic evaluation studies, many of them conducted or sponsored by the manufacturers of the technologies assessed, has been published in recent years [3–7].

Although there is a substantial body of literature for both randomized trials and observational studies indicating the existence of a positive and statistically significant relationship between sources of funding and study results [8–12], this so-called “sponsorship bias” has received relatively little attention in other scientific areas such as cost-effectiveness research (despite the exploiting of opportunities to make diverse assumptions [13]). When available, most studies analyzing a sponsorship bias in cost-effectiveness literature deal with high-cost drugs (mainly oncologic drugs) or new technologies with little clinical evidence at a population-level. However, for certain drugs with a well established use in clinical practice, sponsorship bias has hardly been addressed, including for example the case of specific cardiovascular therapies. Statins (or HMG-CoA reductase inhibitors) lower cholesterol levels, reduce cardiovascular (CV) events and mortality, and are generally well tolerated, with serious side effects being unusual [14,15]. While evidence of the possible benefits of statins in low-risk populations accumulates [16] and prices decline because of the expiry of most patents and the rapid introduction of low-price generics [17], many countries are witnessing a significant expansion in statin prescribing. However, controversies about the real-world clinical effectiveness of statin therapy in primary CV prevention and also regarding the proportion of people eligible for treatment (e.g. financial constraints and the narrow benefit-risk balance in people at low CV risk) still persist [18–27]. In this context, this study aims to examine the association between the funding source of published cost-effectiveness analyses of statin use for both primary and secondary CV prevention and the study conclusions.

## Methods

### Data sources

We conducted a systematic search of PubMed/MEDLINE (up to June 2011) to identify relevant articles. The search strategy consisted of combinations of free text and medical subject heading (MeSH) terms distributed into two blocks: “health economics” and “licensed statins”. We used the following search terms: (*"Economics, Pharmaceutical"[MeSH] OR "Cost-Benefit Analysis"[MeSH] OR "Drug Costs"[MeSH] OR "Costs and Cost Analysis"[MeSH] OR "Quality-Adjusted Life Years"[MeSH] OR "cost effectiveness"[All Fields] OR "pharmacoeconomics"[All Fields]*) *AND* (*("pitavastatin"[Supplementary Concept] OR "pitavastatin"[All Fields]) OR ("rosuvastatin"[Supplementary Concept] OR "rosuvastatin"[All Fields]) OR ("pravastatin"[MeSH Terms] OR "pravastatin"[All Fields]) OR ("lovastatin"[MeSH Terms] OR "lovastatin"[All Fields]) OR ("simvastatin"[MeSH Terms] OR "simvastatin"[All Fields]) OR ("atorvastatin"[Supplementary Concept] OR "atorvastatin"[All Fields])*). Furthermore, we also conducted complementary manual reference searches.

### Study selection

We decided to include CEAs of one or more statins. Two researchers with expertise in health economics and evidence synthesis research screened the titles and abstracts and identified and excluded all papers not meeting any prespecified criteria. The same researchers independently evaluated the remaining studies as full papers. Any disagreements were resolved by consensus.

Inclusion criteria include: 1) Original full CEAs where both costs and outcomes have been measured; 2) Papers reporting study outcomes as the incremental cost per quality-adjusted life years (QALY) and/or life years gained (LYG); 3) Papers assessing one statin compared to another statin or comparing different doses of the same statin (e.g. high-dose versus low-dose comparisons) or papers assessing statins compared to a non-active alternative (e.g. placebo). Studies with combination therapies (e.g. statin and another drug) were included if there was a comparison with a statin alone and/or a non-active alternative; 4) Papers published in English or Spanish. Exclusion criteria were: 1) Editorials, letters, commentaries and case reports; 2) Reviews; 3) Papers assessing statin drugs compared to non-statin cholesterol-lowering drugs; 4) Papers that did not consider a particular statin explicitly.

### Data extraction

For each paper included, we extracted author, year and journal of publication, journal impact factor and subject category, affiliation of the first author, region of study, funding source, prevention category, interventions of study and qualitative conclusions. We also extracted the reported ICERs. We used the following definitions: 1) Impact factor was obtained from 2010 Journal Citation Reports (JCR) and was categorized into quartiles; 2) Subject category or categories which best represent the journal’s field according to the 2010 JCR; 3) Affiliation of the first author was obtained from the article and categorized into: academia, private (e.g. pharmaceutical industry or consulting firm), hospital or other. We further identified the number of articles with at least 1 pharmaceutical industry or consulting firm employee; 4) Region of study was recorded according to the context of the population and targeted country; 5) Source of funding of each paper was presented in 3 categories as follows: industry, non-profit and no funding/none disclosed. A study was considered industry-sponsored if all or part of the funding support was from private industry; 6) Interventions for the primary study outcome were classified into 2 categories: “statin versus statin” or “statin versus non-active alternative”. In statin versus statin (head-to-head) comparisons, the intervention (drug-specific) was defined as the statin identified as such by the lead authors of the paper or the newest statin (the most recent approval date in developed countries e.g., U.S. FDA, European mutual recognition procedure, etc.); 7) The prevention category included: primary CV prevention if it involved the prevention of cardiovascular events in patients who had no clinical evidence of CV disease, secondary CV prevention if the aim was to prevent events in patients who already had clinical evidence of CV disease, or both; 8) Conclusions reported in the published article were defined as: favourable if the intervention using statins was clearly claimed to be the preferred choice (e.g. cited as “cost-effective”, “reduced costs”, “produced cost savings”, “an affordable option”); unfavourable if the final comments were negative (e.g., the statin is “unlikely to be cost-effective”, “produced higher costs”, “is economically unattractive” or “exceeded conventional thresholds of good value for money”); and neutral when the intervention of interest did not surpass the comparator, and/or when some uncertainty was expressed in the conclusions. 9) The ICER is a measure of the additional cost per additional unit of the health gain produced by one intervention compared to another. All incremental costs per QALY/LYG data in the standard base case analysis of the articles reviewed (either the status quo or a projected base case which considered the ‘clinical practice as usual’ or ‘do nothing’ scenario) were extracted and assessed in an alternative exploratory analysis. Costs of the interventions assessed were extracted from studies evaluating costs in a range of international currencies. Therefore, ICER figures were converted (from available rates) to 2011 US dollars ($) by using currency values, to allow meaningful comparisons of ICER between industry-sponsored studies and non-sponsored ones. If an article stated that the intervention was economically dominant (e.g. statin therapy saved costs and provided health benefits) but the results were not reported, we considered values to be equal to null. Because each selected article often included specific base-case scenarios differentiating subgroups of patients (e.g. age and sex), settings and/or perspectives, each CEA may have contributed to more than one ICER.

### Statistical analysis

A descriptive analysis was performed using frequency and percentage counts. We established relationships between qualitative conclusions and 1) type of sponsorship and 2) journal impact factor using Fisher’s exact tests in 2x2 contingency tables (the differences between two proportions). A two-tailed *p* value < 0.05 against the null hypothesis of no relationship was considered statistically significant. We used the journal impact factor as the main journal characteristic to additionally examine whether this measure, reflecting the average number of citations of recent articles published in a certain journal, could be a proxy measure of sponsorship bias in cost-effectiveness research. We also presented dot plot graphs exploring the distributions of ICERs estimates by funding source and prevention category in relation to a hypothetical willingness-to-pay threshold of $50,000 per QALY/LYG. All calculations were performed using the STATA package (Version 12, StataCorp LP, College Station, TX, USA). The systematic review has been reported according to PRISMA (Preferred Reporting Items for Systematic Reviews and Meta-Analyses) guidelines [28].

## Results

**Figure 1:**
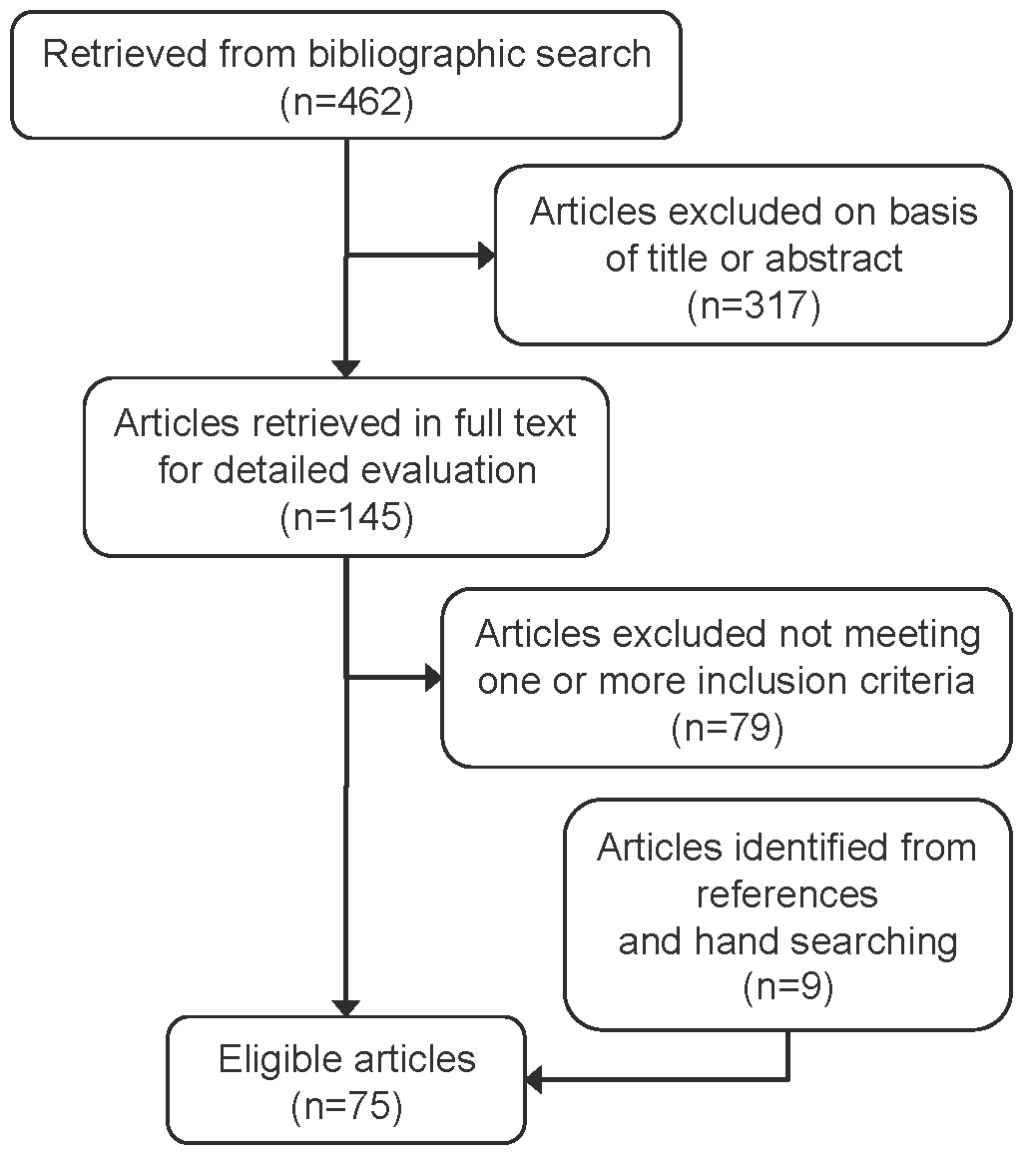
Flow diagram of study selection **process**.

Our initial literature search yielded 462 citations. After screening titles and abstracts, 145 full text reports were obtained and 9 additional articles were identified from the reference lists of retrieved articles and hand searching. Overall, 75 articles met our inclusion criteria (see Figure 1). The full references for the articles included are listed in the Additional Files (Table S1: “List of Included References”). The PRISMA checklist is provided in the Additional Files (Table S2: “PRISMA Checklist”).

Table 1 details the characteristics of the articles reviewed. The studies, most of which were based in Europe and North America, were principally published in journals with a high-impact factor (Tables 1 and 2). Fifty-two articles (69.3%) compared statins versus non-active alternatives. The most frequent statin involved in the studies (defined as the intervention) was atorvastatin (n=21, 28.0%), followed by pravastatin (n=18, 24.0%) and simvastatin (n=16, 21.3%) (Table 3). Studies were funded primarily by industry (64.0%), followed by non-profit or government organizations (18.7%). Secondary CV prevention represented 42.7% of the articles, followed by primary CV prevention (38.7%) or both primary and secondary CV prevention (18.7%). From the 75 articles included, in 44.0% of them the first author was employed in academia and 41.3% of studies had at least 1 author with a pharmaceutical company or consulting firm affiliation. The summary characteristics and results of selected articles are provided in the Additional Files (Table S3: “Summary Characteristics of Selected Articles” and Table S4: “Base case ICERs for Selected Articles”).

**Table 1:** Characteristics of articles included in the study (n=75).

	**n (%)**
**Year of publication**	
1991-1995	5 (6.7)
1996-2000	23 (30.7)
2001-2005	14 (18.7)
2006-2011	33 (44.0)
**Journal impact factor, quartiles (Q)**	
Q1	44 (58.7)
Q2	13 (17.3)
Q3	5 (6.7)
Q4	1 (1.3)
None	12 (16.0)
Journal subject category*	
Cardiac & cardiovasc. systems/peripheral vasc. disease	22 (29.3)
Medicine, general & internal	18 (24.0)
Pharmacology & pharmacy	10 (13.3)
Health care sciences & services/health policy & serv.	9 (12.0)
Endocrinology & metabolism	3 (4.0)
Other/none	13 (17.3)
**Region of study**	
Europe	37 (49.3)
North America	33 (44.0)
Other	5 (6.7)
First author affiliation**	
Academia	33 (44.0)
Private	21 (28.0)
Hospital	18 (24.0)
Other	3 (4.0)
**One or more author industry or consulting firm employee**	
Yes	31 (41.3)
No	44 (58.7)
**Funding source**	
Industry	48 (64.0)
Non-profit/Government	14 (18.7)
No funding/none disclosed	13 (17.3)
**Interventions**	
Statin versus statin	23 (30.7)
Statin versus non-active alternative	52 (69.3)
**Prevention category**	
Primary	29 (38.7)
Secondary	32 (42.7)
Both	14 (18.7)

* Subject category as reported in the 2010 Journal Citation Report. Data do not equal 100% because of rounding errors.

** The affiliation categories of the first author were mutually exclusive according to the following hierarchie: private > academia > hospital > other.

**Table 2:** Journals and impact factor scores by funding source (N=75).

**Journal**	**Impact factor (2010)**	**Industry-sponsored studies**		**Non-profit/ government studies**		**No funding/ none disclosure**	
Circulation	14.8	1		0		0	
J Am Coll Cardiol	14.3	1		0		0	
Eur Heart J	10.0	3		0		2	
Int J Cardiol	6.8	0		0		1	
Am Heart J	5.0	1		1		0	
Heart	4.7	0		1		0	
Atherosclerosis	4.1	1		0		0	
Am J Cardiol	3.7	3		1		1	
Can J Cardiol	2.2	1		1		0	
Rev Esp Cardiol	2.2	1		0		0	
Arch Cardiovasc Dis	1.2	1		0		0	
Acta Cardiol	0.6	1		0		0	
***Totalcardiovasc(n=22).***		***14 (67%)***		***3 (14%)***		***4 (19%)***	
N Engl J Med	53.5	1		0		0	
JAMA	30.0	1		1		0	
Ann Intern Med	16.7	0		2		0	
BMJ	13.5	2		0		1	
Arch Intern Med	10.6	0		1		1	
CAMJ	9.0	1		0		0	
J Intern Med	5.9	1		0		0	
Med J Aust	2.7	1		1		0	
Curr Med Res Opin	2.6	2		1		1	
***Totalmedicine(n=18)***		***9 (50%)***		***6 (33%)***		***3 (17%)***	
Pharmacoeconomics	3.4	6		0		0	
Clin Ther	2.5	2		0		0	
Ann Pharmacother	2.2	1		0		0	
Clin Drug Investing	1.6	1		0		0	
***Totalpharmacol(n=10).***		***10 (100%)***		***0(0%)***		***0(0%)***	
Health Technol Assess	4.2	0		3		0	
Value Health	2.3	3		0		0	
Eur J Health Econ	1.8	2		0		0	
Health Policy	1.4	0		0		1	
***Totalhealthpolicy(n=9)***		***5 (56%)***		***3 (33%)***		***1 (11%)***	
Diabetes care	7.1	1		0		0	
Diabetologia	7.0	2		0		0	
***Totalendocrin(n=3).***		***3 (100%)***		***0(0%)***		***0(0%)***	
Rev Neurol	1.2	1		0		0	
Other journals*	–	6		1		5	
***Totalothers(n=13)***		***7 (54%)***		***1 (8%)***		***5 (38%)***	

Note: PubMed/MEDLINE and Index Medicus abbreviations of journal titles are used.

* Journals not included in the 2010 Journal Citation Report.

**Table 3:** Number of cost-effectiveness analyses comparing drug–drug interventions and/or drug-non active control.

	**Alternatives**						
**Interventions**	Atorva	Fluva	Lova	Prava	Rosuva	Simva	Non-active
Atorvastatin	4^*^	2	0	0	1	2	12
Fluvastatin	0	0	0	1	0	0	4
Lovastatin	0	0	2^*^	0	0	0	3
Pravastatin	0	0	0	0	0	0	18
Rosuvastatin	1	0	0	0	0	3	6
Simvastatin	0	0	0	0	1	2^*^	13

* Includes high-dose versus low-dose statin monotherapy and/or combination therapies.

Overall, industry-sponsored studies were much less likely to report unfavourable or neutral conclusions than non-sponsored ones (0% [0 out of 54] versus 37.1% [13 out of 35], *p*<0.001) (Table 4). For primary CV prevention, the proportion of studies with unfavourable or neutral conclusions was 0% [0 out of 24] for industry-sponsored studies versus 57.9% [11 out of 19] for non-sponsored studies (*p*<0.001). No statistically significant differences were identified for studies evaluating secondary CV prevention (0% [0 out of 30] versus 12.5% [2 out of 16], respectively; *p*=0.222). The journal impact factor was not significantly associated with study conclusions, as shown in Table 5.

**Table 4:** Study conclusions by funding source.

**Conclusion by prevention category**	**Industry sponsored**	Non-industry sponsored^*^	**P-value**
**Primary prevention (n=43)**			
Favourable	24	8	<0.0001
Unfavourable/neutral	0	11	
**Secondary prevention (n=46)**			
Favourable	30	14	0.2221
Unfavourable/neutral	0	2	
**All (n=89)**			
Favourable	54	22	<0.0001
Unfavourable/neutral	0	13	

* Includes non-profit, no funding and/or no disclosure.

**Table 5:** Study conclusions by journal impact factor.

**Conclusion by prevention category**	**High IF journals**	**Low IF journals**	**P-value**
**Primary prevention (n=43)**			
Favourable	24	8	0.4906
Unfavourable/neutral	10	1	
**Secondary prevention (n=46)**			
Favourable	35	9	0.9090
Unfavourable/neutral	1	1	
**All (n=89)**			
Favourable	59	17	0.8402
Unfavourable/neutral	11	2	

IF: impact factor 2010; High IF journals: included in the first and second quartile in the 2010 Journal Citation Report; low IF journals: included in the third and fourth quartile, and journals not included in the 2010 Journal Citation Report.

**Figure 2:**
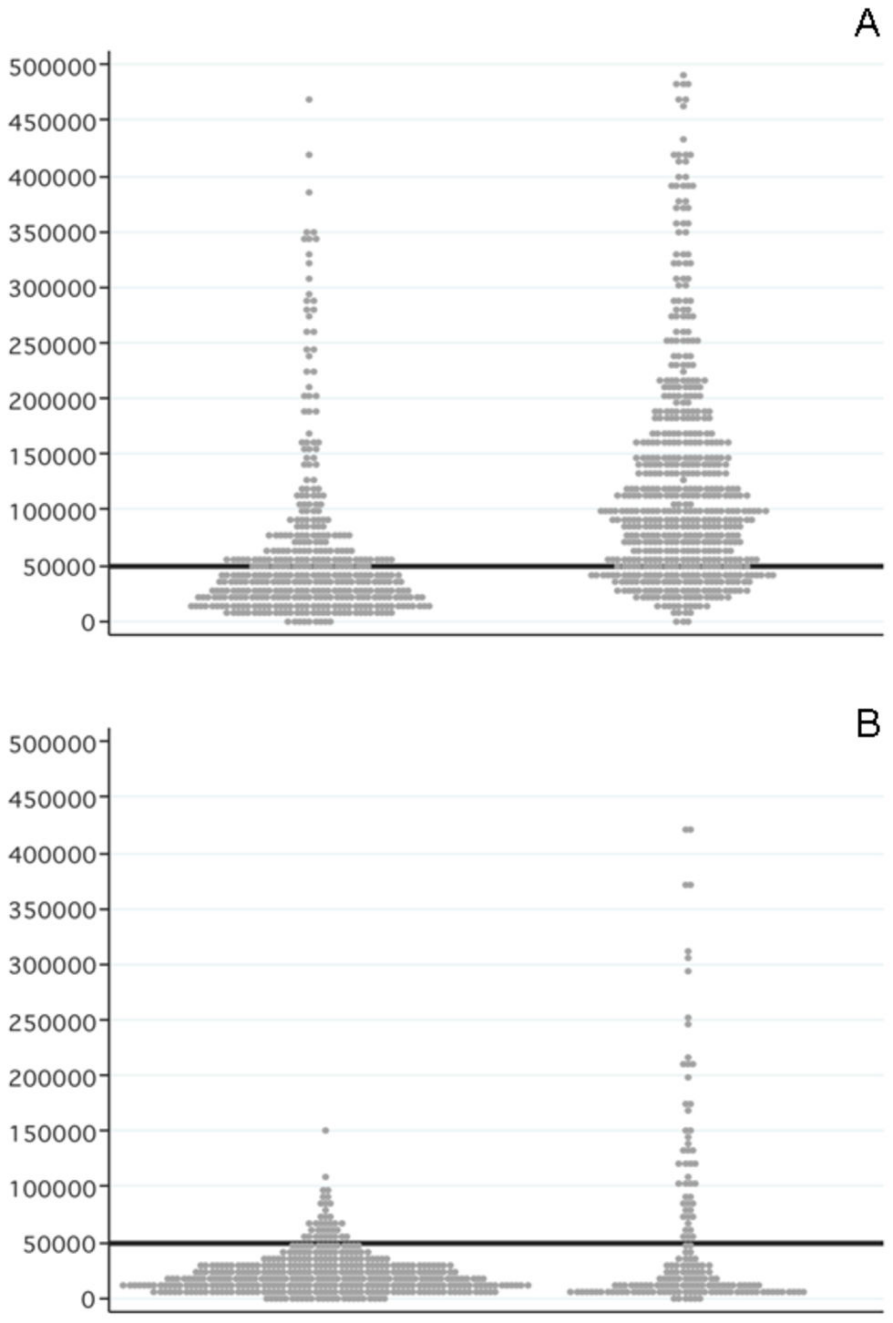
Variations in cost-effectiveness results by funding source and prevention category: a) Primary CV prevention and b) Secondary CV prevention. CV: Cardiovascular. Note: Each dot represents an incremental cost (in US$) per QALY/LYG in the reviewed articles. The horizontal line represents the willingness-to-pay threshold.

A total of 1,612 ICERs were obtained from the 75 CEAs included in our study. Figure 2 shows the variability in the estimated incremental costs per QALYs/LYGs gained by funding source and prevention category. For primary CV prevention, ICERs reported in industry-sponsored studies were more likely to fall below the $50,000 willingness-to-pay threshold than in non-sponsored ones (61.6% [253 out of 411] versus 22.3% [135 out of 605], *p*<0.001). For secondary CV prevention, the majority of ICERs considered fell below the threshold in both industry and non-industry sponsored studies (91.2% [386 out of 423] versus 68.8% [119 out of 173], respectively) (*p*<0.001).

## Discussion

This systematic analysis identified a significant association between the conclusions of statin cost-effectiveness and industry sponsorship. Pharmaceutical company-sponsored studies were significantly less likely to reach neutral or unfavourable conclusions than non-pharmaceutical company-sponsored studies. In fact, all pharmaceutical company-sponsored studies concluded that the corresponding statin had a favourable cost-effectiveness ratio compared to any of the other agents (including competing statins) or placebo. An alternative exploratory analysis showed that ICERs reported in industry-sponsored studies were more likely to fall below the $50,000 willingness-to-pay threshold than those reported in non-sponsored ones. The difference in the “positive values” was especially striking in primary CV prevention.

These results are consistent with other studies assessing the relationship between the source of funding and the cost-effectiveness findings, specifically for oncologic drugs [29–33], neuropsychiatric drugs [34–36], venous thromboembolism pharmacologic prevention [37], drug-eluting stents [38], bisphosphonates for osteoporosis treatment [39], Pap tests for cervical cancer diagnosis [40]. They are also consistent with the CEAs submitted by manufacturers to health technology assessment agencies [5,6], and/or reviews selecting general cost-effectiveness studies [4,7,41,42]. Regarding the sponsorship bias of cost-effectiveness of statin use in CV prevention, our results confirm and extend those reported by Franco and colleagues [43]. They conducted a review including 24 studies between 1990 and 2002 with 216 ICER, showing that differences in cost-effectiveness ratios between industry and non-industry sponsored studies were higher the lower the level of CV risk.

The mechanisms through which authors (and their sponsors) may potentially obtain more favourable cost-effectiveness results have been little studied, but several possibilities have been suggested [30,34,44–49]. For example, the so-called “publication bias” against untraceable or unpublished papers with non-significant or unfavourable results could be present in some CEA research [6]. Another source of sponsorship bias could be that dragged from efficacy studies, which was shown by Bero and colleagues [8], who reported in a cross-sectional study of 192 randomized trials that comparative trials of statins were more likely to report outcomes favouring the sponsor’s statin compared to the comparator drug.

Regarding journal characteristics, it could be argued that they could be associated with the reliability of study findings and conclusions as the quality of the reporting could vary among journals. In the study conducted by Bell and colleagues [4], a CEA review of the English-language articles indexed in PubMed/MEDLINE since 2001, the authors observed that the more quoted journals with an impact factor above 4 were less likely to publish favourable results (e.g. ratios below $20,000-50,000/QALY) than less quoted journals with a lower impact factor. In our research, however, the journal impact factor was not significantly associated with study conclusions. In fact, these differences with our results are probably part of the high prevalence of studies published in high-impact journals in our sample (e.g. at least three-quarters of our selected articles were published in top journals positioned in the higher quartiles of their specialty), this not being a particularity in the cardiovascular area.

If sponsorship bias is a relevant explanation for our results, making decisions (e.g. selection of certain drugs in national formularies) based on results from industry-sponsored evaluations might be considered (arguably wrongly) to be a futile exercise. In such hypothetical situation, an economic evaluation could make healthcare services even more inefficient, increasing spending on medication prescription without obtaining clear benefits in the health of the population.

Our study has several limitations. First, the extent of the relationships between the project, the authors and the sponsors was limited to the information publicly available in the corresponding paper. There were no further inquiries or attempts to verify the truthfulness of the conflict of interest statements. And only information about funding and authors’ affiliation was taken into account in the main analyses, without considering other possible sources of conflict of interest. Additionally, although similar methods have been used in earlier studies [7–9,32,33,35], we acknowledge there could be some amount of subjectivity involved in assessing whether the conclusions of a paper were biased towards the sponsor’s drug. To minimize this potential reporting bias we used explicit inclusion criteria, pre-defined study definitions, and data coding and consensus among researchers in the case of disagreement. Second, study eligibility was restricted to the English and Spanish languages for practical reasons. However, Egger and colleagues [50] pointed out the existence of a language bias in the publication of randomized trials from non-English countries, with a greater likelihood of publication of significant results in English language journals. It is difficult to determine to what extent this bias affects cost-effectiveness research in non-English journals, which is usually focused on a local market. In any case, the language bias is a limitation to consider in this study. Third, while applying the same economic models to different populations or countries is a common practice in CEAs and, therefore, some degree of autocorrelation between studies is expected, we analyzed each study as an independent observation. This effect may have distorted the strength of the association between the type of funding and cost-effectiveness conclusions. We emphasise that caution is needed when comparing the incremental cost-effectiveness figures, as we were unable to analyse further by stratifying them based on baseline cardiac risks.

Generally, publication planning by the pharmaceutical industry has been suggested to undermine the research process in several ways, as marketing interests may influence the research process at multiple different times: during decisions over which studies to conduct, study design, analytic approach, writing of manuscripts, and whether or not to publish research findings [51,52]. It is possible that in the case of high cost drugs, industry funded economic evaluations are designed from the outset to suggest greater cost-effective. These market access strategies are relevant because they could generate confusion about the true therapeutic added-value of new drugs, and result in a considerable loss of credibility of the cost-effectiveness literature, finally leading to concerns about the applicability of economic analyses and impeding the use of economic evaluations in medical decision-making.

Unfortunately, it does not seem to be a problem that can be easily solved and it is hard to identify any effective strategy to limit the potential for manipulation. Full disclosure of the authors’ conflict of interest is the most widespread approach and some journals apply restrictive policies on industry-sponsored cost-effectiveness studies [53,54]. The use of guidelines or good practices for economic evaluation is strongly recommended as a possible solution by journals, experts and professional societies [55], and several editorialists have also proposed strengthening the peer-review processes and making them more transparent [43,44,54]. All of these strategies are probably sound and indispensable in the current context, but they all also have important weaknesses. Policies on conflict of interest so far have not been sufficient to reduce sponsors’ control of the results of cost-effectiveness studies, and the extensive collaborative networks between manufacturers, contracted consulting firms, academia and healthcare centres [56] create situations that allow industry sponsorship to be concealed. The use of methodological guidelines does not prevent slipping "reasonable favourable assumptions" into economic models (as Evans stated “researchers know what sponsors want to hear and what will affect the probability of subsequent support” [13]). Additionally, the accurate peer review of economic models can be extremely laborious [57]. In fact, to redo with local data some parameters from a previous model developed for a pharmaceutical company and to post a “new” local or updated article can be much less costly than the evaluation of that article by the journal’s reviewers [58]. Public reporting of models and unhindered access to the methods used to produce cost-effectiveness estimates (so that they can be examined in detail) could help. If public authorities really want CEAs to become a credible tool for supporting their own decision-making, they will have to encourage and finance the conducting of independent cost-effectiveness research.

In summary, our study demonstrates an important sponsorship bias in the cost-effectiveness of statins for CV prevention, and adds a new piece to the growing body of literature suggesting a major distortion in our knowledge about the true efficiency (“good value for money”) of drugs and medical technologies. The evaluation of medical technologies is not only, or even primarily, the evaluation of their cost-effectiveness. The dimensions of safety, comparative effectiveness and other socially pertinent criteria should be reconciled in determining health priorities. But economic evaluation, by its intrinsic logic for identifying and assessing all costs and benefits, is one of the most informative approaches to making decisions for funding (or not) health technologies, especially when working with imperfect information [59]. It is in this ability to operate with imperfect information that many of its advantages and the potential for interested manipulation reside. Correcting or mitigating these risks is probably one of the most important issues in the cost-effectiveness of medical technologies in order to ensure they reach their full potential to support decision-making processes.

## Supporting Information

Table S1 **List of Included References.**
(PDF)

Table S2 **PRISMA Checklist.**
(PDF)

Table S3 **Summary Characteristics of Selected Articles.**
(PDF)

Table S4 **Base case ICERs for Selected Articles.**
(PDF)
