# Supplementary material for: When Are Statins Cost-Effective in Cardiovascular Prevention? A Systematic Review of Sponsorship Bias and Conclusions in Economic Evaluations of Statins
Source: PLoS One. 2013 Jul 8;8(7):e69462. doi: 10.1371/journal.pone.0069462 (PMC3704635; doi:10.1371/journal.pone.0069462)
Supplement: Table S1 — List of Included References. (PDF) [file pone.0069462.s001.pdf]

Additional file 1_ List of Included References

Studies included in the systematic review (by year of publication)

1991-1995

1. Goldman L, Weinstein MC, Goldman PA, Williams LW. Cost-effectiveness of HMG-CoA reductase inhibition for primary and secondary prevention of coronary heart disease. JAMA. 1991;265(9):1145-51.

2. Hay JW, Wittels EH, Gotto AM Jr. An economic evaluation of lovastatin for cholesterol lowering and coronary artery disease reduction. Am J Cardiol. 1991;67(9):789-96.

3. Goldman L, Goldman PA, Williams LW, Weinstein MC. Cost-effectiveness considerations in the treatment of heterozygous familial hypercholesterolemia with medications. Am J Cardiol. 1993;72(10):75D-79D.

4. Martens LL, Guibert R. Cost-effectiveness analysis of lipid-modifying therapy in Canada: comparison of HMG-CoA reductase inhibitors in the primary prevention of coronary heart disease. Clin Ther. 1994;16(6):1052-62.

5. Hamilton VH, Racicot FE, Zowall H, Coupal L, Grover SA. The cost-effectiveness of HMG-CoA reductase inhibitors to prevent coronary heart disease. Estimating the benefits of increasing HDL-C. JAMA. 1995;273(13):1032-8.

1996-2000

6. Johannesson M, Borgquist L, Jönsson B, Lindholm LH. The cost effectiveness of lipid lowering in Swedish primary health care. The CELL Study Group. J Intern Med. 1996;240(1):23-9.

7. Jönsson B, Johannesson M, Kjekshus J, Olsson AG, Pedersen TR, Wedel H. Cost-effectiveness of cholesterol lowering. Results from the Scandinavian Simvastatin Survival Study (4S). Eur Heart J. 1996;17(7):1001-7.

8. Ashraf T, Hay JW, Pitt B, Wittels E, Crouse J, Davidson M, Furberg CD, Radican L. Cost-effectiveness of pravastatin in secondary prevention of coronary artery disease. Am J Cardiol. 1996;78(4):409-14.

9. Rivière M, Wang S, Leclerc C, Fitzsimon C, Tretiak R. Cost-effectiveness of simvastatin in the secondary prevention of coronary artery disease in Canada. CMAJ. 1997;156(7):991-7.

10. Johannesson M, Jönsson B, Kjekshus J, Olsson AG, Pedersen TR, Wedel H. Cost effectiveness of simvastatin treatment to lower cholesterol levels in patients with coronary heart disease. Scandinavian Simvastatin Survival Study Group. N Engl J Med. 1997;336(5):332-6.

11. Caro J, Klittich W, McGuire A, Ford I, Norrie J, Pettitt D, McMurray J, Shepherd J. The West of Scotland coronary prevention study: economic benefit analysis of primary prevention with pravastatin. BMJ. 1997;315(7122):1577-82.

12. Perreault S, Hamilton VH, Lavoie F, Grover S. Treating hyperlipidemia for the primary prevention of coronary disease. Are higher dosages of lovastatin cost-effective? Arch Intern Med. 1998;158(4):375-81.

13. Lindholm L, Hallgren CG, Boman K, Markgren K, Weinehall L, Ogren JE. Cost effectiveness analysis with defined budget : how to distribute resources for the prevention of cardiovascular disease ? Health Policy 1999;48:155-170

14. Pharoah P. Economic benefit analysis of primary prevention with pravastatin. Modelling economic benefits after such long term treatment is inappropriate. BMJ. 1998;316(7139):1241-2.

15. Troche CJ, Tacke J, Hinzpeter B, Danner M, Lauterbach KW. Cost-effectiveness of primary and secondary prevention in cardiovascular diseases. Eur Heart J. 1998;19 Suppl C:C59-65.

16. Muls E, Van Ganse E, Closon MC. Cost-effectiveness of pravastatin in secondary prevention of coronary heart disease: comparison between Belgium and the United States of a projected risk model. Atherosclerosis. 1998;137 Suppl:S111-6.

17. Huse DM, Russell MW, Miller JD, Kraemer DF, D'Agostino RB, Ellison RC, Hartz SC. Cost-effectiveness of statins. Am J Cardiol. 1998;82(11):1357-63.

18. Grover SA, Coupal L, Paquet S, Zowall H. Cost-effectiveness of 3-hydroxy-3 methylglutaryl-coenzyme A reductase inhibitors in the secondary prevention of cardiovascular disease: forecasting the incremental benefits of preventing coronary and cerebrovascular events. Arch Intern Med. 1999;159(6):593-600.

19. Caro J, Klittich W, McGuire A, Ford I, Pettitt D, Norrie J, Shepherd J. International economic analysis of primary prevention of cardiovascular disease with pravastatin in WOSCOPS. West of Scotland Coronary Prevention Study. Eur Heart J. 1999;20(4):263-8.

20. Jönsson B, Cook JR, Pedersen TR. The cost-effectiveness of lipid lowering in patients with diabetes: results from the 4S trial. Diabetologia. 1999;42(11):1293-301.

21. Elliott WJ, Weir DR. Comparative cost-effectiveness of HMG-CoA reductase inhibitors in secondary prevention of acute myocardial infarction. Am J Health Syst Pharm. 1999;56(17):1726-32.

22. Pickin DM, McCabe CJ, Ramsay LE, Payne N, Haq IU, Yeo WW, Jackson PR. Cost effectiveness of HMG-CoA reductase inhibitor (statin) treatment related to the risk of coronary heart disease and cost of drug treatment. Heart. 1999;82(3):325-32.

23. Morris S, Godber E. Choice of cost-effectiveness measure in the economic evaluation of cholesterol-modifying pharmacotherapy. An illustrative example focusing on the primary prevention of coronary heart disease in Canada. Pharmacoeconomics. 1999;16(2):193-205.

24. Ebrahim S, Davey Smith G, McCabe C, Payne N, Pickin M, Sheldon TA, Lampe F, Sampson F, Ward S, Wannamethee G. What role for statins? A review and economic model. Health Technol Assess. 1999;3(19):i-iv, 1-91.

25. Prosser LA, Stinnett AA, Goldman PA, Williams LW, Hunink MG, Goldman L, Weinstein MC. Cost-effectiveness of cholesterol-lowering therapies according to selected patient characteristics. Ann Intern Med. 2000;132(10):769-79.

26. Ganz DA, Kunts KM, Jacobson GA, Avorn J. Cost-effectiveness of 3-hydroxy-3 methylglutaryl coenzyme A reductase inhibitor therapy in older patients with myocardial infarction. Ann Intern Med 2000;132(10):780-7.

27. Grover SA, Coupal L, Zowall H, Dorais M. Cost-effectiveness of treating hyperlipidemia in the presence of diabetes : who should be treated? Circulation. 2000;102(7):722-7.

28. Caro JJ, Huybrechts KF, De Backer G, De Bacquer D, Closon MC. Are the WOSCOPS clinical and economic findings generalizable to other populations? A case study for Belgium. The

WOSCOPS Economic Analysis Group. West of Scotland Coronary Prevention Study. Acta Cardiol. 2000;55(4):239-46.

2001-2005

29. Grover SA, Coupal L, Zowall H, Alexander CM, Weiss TW, Gomes DR. How cost-effective is the treatment of dyslipidemia in patients with diabetes but without cardiovascular disease? Diabetes Care. 2001;24(1):45-50.
30. Russell MW, Huse DM, Miller JD, Kraemer DF, Hartz SC. Cost effectiveness of HMG-CoA reductase inhibition in Canada. Can J Clin Pharmacol. 2001;8(1):9-16.
31. Shepherd J. Economics of lipid lowering in primary prevention: lessons from the West of Scotland Coronary Prevention Study. Am J Cardiol. 2001;87(5A):19B-22B.
32. Tsevat J, Kuntz KM, Orav EJ, Weinstein MC, Sacks FM, Goldman L. Cost-effectiveness of pravastatin therapy for survivors of myocardial infarction with average cholesterol levels. Am Heart J. 2001;141(5):727-34.
33. van Hout BA, Simoons ML. Cost-effectiveness of HMG coenzyme reductase inhibitors; whom to treat? Eur Heart J. 2001;22(9):751-61.
34. Chau J, Cheung BM, McGhee SM, Lauder IJ, Lau CP, Kumana CR. Cost-effectiveness analysis of applying the Cholesterol and Recurrent Events (CARE) study protocol in Hong Kong. Hong Kong Med J. 2001;7(4):360-8.
35. Lim SS, Vos T, Peeters A, Liew D, McNeil JJ. Cost-effectiveness of prescribing statins according to pharmaceutical benefits scheme criteria. Med J Aust. 2001;175(9):459-64.
36. Glasziou PP, Eckermann SD, Mulray SE, Simes RJ, Martin AJ, Kirby AC, Hall JP, Caleo S, White HD, Tonkin AM. Cholesterol-lowering therapy with pravastatin in patients with average cholesterol levels and established ischaemic heart disease: is it cost-effective? Med J Aust. 2002;177(8):428-34.
37. Barry M, Heerey A. Cost effectiveness of statins for the secondary prevention of coronary heart disease in Ireland. Ir Med J. 2002;95(5):133-5.
38. Scuffham PA, Chaplin S. An economic evaluation of fluvastatin used for the prevention of cardiac events following successful first percutaneous coronary intervention in the UK. Pharmacoeconomics. 2004;22(8):525-35.
39. Pilote L, Ho V, Lavoie F, Coupal L, Zowall H, Grover SA. Cost-effectiveness of lipid-lowering treatment according to lipid level. Can J Cardiol. 2005;21(8):681-7.
40. Delea TE, Jacobson TA, Serruys PW, Edelsberg JS, Oster G. Cost-effectiveness of fluvastatin following successful first percutaneous coronary intervention. Ann Pharmacother. 2005;39(4):610-6.
41. Nagata-Kobayashi S, Shimbo T, Matsui K, Fukui T. Cost-effectiveness of pravastatin for primary prevention of coronary artery disease in Japan. Int J Cardiol. 2005;104(2):213-23.
42. Scuffham PA, Chaplin S. A cost-effectiveness analysis of fluvastatin in patients with diabetes after successful percutaneous coronary intervention. Clin Ther. 2005;27(9):1467-77.

2006-2011

43. Tonkin AM, Eckermann S, White H, Friedlander D, Glasziou P, Magnus P, Kirby A, Mulray S, Denton M, Sallaberger M, Hunt D, Simes J; LIPID Study Group. Cost-effectiveness of cholesterol-lowering therapy with pravastatin in patients with previous acute coronary syndromes aged 65 to 74 years compared with younger patients: results from the LIPID study. Am Heart J. 2006;151(6):1305-12.

44. Fernández de Bobadilla J, López de Sa E, Alonso Troncoso I, Moreno Gómez R, Rubio-Terrés C, Soto Alvarez J. [Cost-effectiveness analysis of the use of atorvastatin in patients with type 2 diabetes mellitus: a pharmacoeconomic model of the CARDS study]. An Med Interna. 2006;23(5):213-9. Spanish.

45. Heart Protection Study Collaborative, Mihaylova B, Briggs A, Armitage J, Parish S, Gray A, Collins R. Lifetime cost effectiveness of simvastatin in a range of risk groups and age groups derived from a randomised trial of 20,536 people. BMJ. 2006;333(7579):1145.

46. Kohli M, Attard C, Lam A, Huse D, Cook J, Bourgault C, Alemao E, Yin D, Marentette M. Cost effectiveness of adding ezetimibe to atorvastatin therapy in patients not at cholesterol treatment goal in Canada. Pharmacoeconomics. 2006;24(8):815-30.

47. Walshe V, Nash A, Barry M. Cost effectiveness of statin therapy for the primary prevention of coronary heart disease. Ir Med J. 2006;99(5):144-5.

48. Raikou M, McGuire A, Colhoun HM, Betteridge DJ, Durrington PN, Hitman GA, Neil HA, Livingstone SJ, Charlton-Menys V, Fuller JH; CARDS Investigators. Cost-effectiveness of primary prevention of cardiovascular disease with atorvastatin in type 2 diabetes: results from the Collaborative Atorvastatin Diabetes Study (CARDS). Diabetologia. 2007;50(4):733-40.

49. Lindgren P, Graff J, Olsson AG, Pedersen TJ, Jönsson B; IDEAL Trial Investigators. Cost-effectiveness of high-dose atorvastatin compared with regular dose simvastatin. Eur Heart J. 2007;28(12):1448-53.

50. Ward S, Lloyd Jones M, Pandor A, Holmes M, Ara R, Ryan A, Yeo W, Payne N. A systematic review and economic evaluation of statins for the prevention of coronary events. Health Technol Assess. 2007;11(14):1-160, iii-iv.

51. Alonso R, Fernández de Bobadilla J, Méndez I, Lázaro P, Mata N, Mata P. [Cost-effectiveness of managing familial hypercholesterolemia using atorvastatin-based preventive therapy]. Rev Esp Cardiol. 2008;61(4):382-93.

52. Pinto CG, Carrageta MO, Miguel LS. Cost-effectiveness of rosuvastatin in the prevention of ischemic heart disease in Portugal. Value Health. 2008;11(2):154-9.

53. Ramsey SD, Clarke LD, Roberts CS, Sullivan SD, Johnson SJ, Liu LZ. An economic evaluation of atorvastatin for primary prevention of cardiovascular events in type 2 diabetes. Pharmacoeconomics. 2008;26(4):329-39.

54. Newman J, Grobman WA, Greenland P. Combination polypharmacy for cardiovascular disease prevention in men: a decision analysis and cost-effectiveness model. Prev Cardiol. 2008;11(1):36-41.

55. Peura P, Martikainen J, Soini E, Hallinen T, Niskanen L. Cost-effectiveness of statins in the prevention of coronary heart disease events in middle-aged Finnish men. Curr Med Res Opin. 2008;24(6):1823-32.

56. Lafuma A, Colin X, Solesse A. Cost-effectiveness of atorvastatin in the prevention of cardiovascular events in diabetic patients: a French adaptation of CARDS. Arch Cardiovasc Dis. 2008;101(5):327-32.

57. Lindgren P, Buxton M, Kahan T, Poulter NR, Dahlöf B, Sever PS, Wedel H, Jönsson B; ASCOT investigators. The lifetime cost effectiveness of amlodipine-based therapy plus atorvastatin compared with atenolol plus atorvastatin, amlodipine-based therapy alone and atenolol-based therapy alone: results from ASCOT1. Pharmacoeconomics. 2009;27(3):221-30.

58. Taylor DC, Pandya A, Thompson D, Chu P, Graff J, Shepherd J, Wenger N, Greten H, Carmena R, Drummond M, Weinstein MC. Cost-effectiveness of intensive atorvastatin therapy in secondary cardiovascular prevention in the United Kingdom, Spain, and Germany, based on the Treating to New Targets study. Eur J Health Econ. 2009;10(3):255-65.

59. Kongnakorn T, Ward A, Roberts CS, O'Brien JA, Proskorovsky I, Caro JJ. Economic evaluation of atorvastatin for prevention of recurrent stroke based on the SPARCL trial. Value Health. 2009;12(6):880-7.

60. Heart Protection Study Collaborative Group. Statin cost-effectiveness in the United States for people at different vascular risk levels. Circ Cardiovasc Qual Outcomes. 2009;2(2):65-72.

61. Ara R, Pandor A, Stevens J, Rees A, Rafia R. Early high-dose lipid-lowering therapy to avoid cardiac events: a systematic review and economic evaluation. Health Technol Assess. 2009;13(34):1-74, 75-118.

62. Wagner M, Goetghebeur M, Merikle E, Pandya A, Chu P, Taylor DC. Cost-effectiveness of intensive lipid lowering therapy with 80 mg of atorvastatin, versus 10 mg of atorvastatin, for secondary prevention of cardiovascular disease in Canada. Can J Clin Pharmacol. 2009;16(2):e331-45.

63. Wagner M, Lindgren P, Merikle E, Goetghebeur M, Jönsson B. Economic evaluation of high-dose (80 mg/day) atorvastatin treatment compared with standard-dose (20 mg/day to 40 mg/day) simvastatin treatment in Canada based on the Incremental Decrease in End-Points Through Aggressive Lipid-Lowering (IDEAL) trial. Can J Cardiol. 2009 Nov;25(11):e362-9.

64. Annemans L, Marbaix S, Webb K, Van Gaal L, Scheen A. Cost effectiveness of atorvastatin in patients with type 2 diabetes mellitus: a pharmacoeconomic analysis of the collaborative atorvastatin diabetes study in the belgian population. Clin Drug Investig. 2010;30(2):133-42.

65. Nherera L, Calvert NW, Demott K, Humphries SE, Neil HA, Minhas R, Thorogood M. Cost-effectiveness analysis of the use of a high-intensity statin compared to a low-intensity statin in the management of patients with familial hypercholesterolaemia. Curr Med Res Opin. 2010;26(3):529-36

66. Soini EJ, Davies G, Martikainen JA, Hu HX, Tunceli K, Niskanen L. Population-based health-economic evaluation of the secondary prevention of coronary heart disease in Finland. Curr Med Res Opin. 2010;26(1):25-36.

67. Rosen VM, Taylor DC, Parekh H, Pandya A, Thompson D, Kuznik A, Waters DD, Drummond M, Weinstein MC. Cost effectiveness of intensive lipid-lowering treatment for patients with congestive heart failure and coronary heart disease in the US. Pharmacoeconomics. 2010;28(1):47-60.

68. Arrospide A, Mar J, Vivancos-Mora J, Rejas-Gutiérrez J, Caro J. [Cost-effectiveness analysis of using high doses of atorvastatin for the secondary stroke prevention in Spain]. Rev Neurol. 2010;51(1):1-11. Spanish.

69. Reckless J, Davies G, Tunceli K, Hu XH, Brudi P. Projected cost-effectiveness of ezetimibe/simvastatin compared with doubling the statin dose in the United Kingdom: findings from the INFORCE study. Value Health. 2010;13(6):726-34.

70. Ohsfeldt RL, Gandhi SK, Smolen LJ, Jensen MM, Fox KM, Gold A, Hsia J. Cost effectiveness of rosuvastatin in patients at risk of cardiovascular disease based on findings from the JUPITER trial. J Med Econ. 2010;13(3):428-37.

71. MacDonald GP. Cost-effectiveness of rosuvastatin for primary prevention of cardiovascular events according to Framingham Risk Score in patients with elevated C-reactive protein. J Am Osteopath Assoc. 2010;110(8):427-36.

72. Slejko JF, Page RL 2nd, Sullivan PW. Cost-effectiveness of statin therapy for vascular event prevention in adults with elevated C-reactive protein: implications of JUPITER. Curr Med Res Opin. 2010;26(10):2485-97.

73. Choudhry NK, Patrick AR, Glynn RJ, Avorn J. The cost-effectiveness of C-reactive protein testing and rosuvastatin treatment for patients with normal cholesterol levels. J Am Coll Cardiol. 2011 Feb 15;57(7):784-91.

74. Ara R, Pandor A, Stevens J, Rafia R, Ward S, Rees A, Durrington P, Reynolds T, Wierzbicki A, Stevenson M. Prescribing high-dose lipid-lowering therapy early to avoid subsequent cardiovascular events: is this a cost-effective strategy? Eur J Cardiovasc Prev Rehabil. 2011 Apr 1. [Epub ahead of print]

75. Michailov GV, Davies GM, Krobot KJ. Cost-effectiveness of extended-release niacin/laropiprant added to a stable simvastatin dose in secondary prevention patients not at cholesterol goal in Germany. Eur J Health Econ. 2011 Apr 5. [Epub ahead of print]
