# Supplementary material for: When Are Statins Cost-Effective in Cardiovascular Prevention? A Systematic Review of Sponsorship Bias and Conclusions in Economic Evaluations of Statins
Source: PLoS One. 2013 Jul 8;8(7):e69462. doi: 10.1371/journal.pone.0069462 (PMC3704635; doi:10.1371/journal.pone.0069462)
Supplement: Table S3 — Summary Characteristics of Selected Articles. (PDF) [file pone.0069462.s003.pdf]

Additional file_3: Summary characteristics of selected studies

No.	Author	Year	Journal	Category	Q	Region	First author affiliati	Assessment	Sponsorship	Explicit threshold	Primary prevention	Secondary prevention
1	Goldman	1991	JAMA	Medicine, General & Int	Q1	North America	Hospital	Statin vs non active	Public or non-profit	Yes	Unfavourable	Favourable
2	Hay	1991	Am J Cardiol	Cardiac & Cardiovascula	Q1	North America	University	Statin vs non active	Private	Yes	Favourable	NA
3	Goldman	1993	Am J Cardiol	Cardiac & Cardiovascula	Q1	North America	Hospital	Statin vs statin	Public or non-profit	Yes	Favourable	NA
4	Martens	1994	Clin Ther	Pharmacology	Q2	North America	Private	Statin vs statin	Private	No	Favourable	NA
5	Hamilton	1995	JAMA	Medicine, General & Int	Q1	North America	Hospital	Statin vs non active	Private	Yes	Favourable	NA
6	Johanesson	1996	J Intern Med	Medicine, General & Int	Q1	Europe	University	Statin vs non active	Private	Yes	Favourable	NA
7	Jönsson	1996	Eur Heart J	Cardiac & Cardiovascula	Q1	Europe	University	Statin vs non active	Private	Yes	NA	Favourable
8	Pharoa	1996	BMJ	Medicine, General & Int	Q1	Europe	Hospital	Statin vs non active	None	No	Neutral	Favourable
9	Ashraf	1996	Am J Cardiol	Cardiac & Cardiovascula	Q1	North America	Private	Statin vs non active	Private	Yes	NA	Favourable
10	Riviere	1997	CMAJ	Medicine, General & Int	Q1	North America	Private	Statin vs non active	Private	Yes	NA	Favourable
11	Caro	1997	BMJ	Medicine, General & Int	Q1	North America	Private	Statin vs non active	Private	No	Favourable	NA
12	Johanesson	1997	N Engl J Med	Medicine, General & Int	Q1	Europe	University	Statin vs non active	Private	No	NA	Favourable
13	Troche	1998	Eur Heart J	Cardiac & Cardiovascula	Q1	Europe	University	Statin vs non active	None	No	Unfavourable	NA
14	Muls	1998	Atherosclerosis	Peripheral Vascular Dis	Q1	Europe	University	Statin vs non active	Private	Yes	NA	Favourable
15	Huse	1998	Am J Cardiol	Cardiac & Cardiovascula	Q1	North America	Private	Statin vs statin	Private	No	Favourable	Favourable
16	Perreault	1998	Arch Intern Med	Medicine, General & Int	Q1	North America	Hospital	Statin vs statin	Public or non-profit	Yes	Favourable	NA
17	Lindholm	1999	Health Policy	Health Care Sciences &	Q3	Europe	Primary care	Statin vs non active	None	Yes	NA	Favourable
18	Grover	1999	Arch Intern Med	Medicine, General & Int	Q1	North America	Hospital	Statin vs non active	None	No	NA	Favourable
19	Morris	1999	Pharmacoeconomics	Pharmacology	Q2	North America	University	Statin vs non active	Private	No	Favourable	NA
20	Ebrahim	1999	Health Technol Assess	Health Care Sciences &	Q1	Europe	University	Statin vs non active	Public or non-profit	Yes	Neutral	Favourable
21	Elliot	1999	Am J Health Syst Phar	NA	NA	North America	University	Statin vs statin	Private	Yes	NA	Favourable
22	Pickin	1999	Heart	Cardiac & Cardiovascula	Q1	Europe	University	Statin vs non active	Public or non-profit	No	Neutral	Favourable
23	Jönsson	1999	Diabetologia	Endocrinology	Q1	Europe	University	Statin vs non active	Private	Yes	NA	Favourable
24	Caro	1999	Eur Heart J	Cardiac & Cardiovascula	Q1	Europe	Private	Statin vs non active	Private	Yes	Favourable	NA
25	Grover	2000	Circulation	Cardiac & Cardiovascula	Q1	North America	Hospital	Statin vs non active	Private	Yes	Favourable	Favourable
26	Prosser	2000	Ann Intern Med	Medicine, General & Int	Q1	North America	University	Statin vs non active	Public or non-profit	Yes	Neutral	Favourable
27	Caro	2000	Acta Cardiol	Cardiac & Cardiovascula	Q4	Europe	Private	Statin vs non active	Private	Yes	Favourable	NA
28	Ganz	2000	Ann Intern Med	Medicine, General & Int	Q1	North America	Hospital	Statin vs non active	Public or non-profit	No	NA	Favourable
29	Shepherd	2001	Am J Cardiol	Cardiac & Cardiovascula	Q1	Europe	Healthcare adminis	Statin vs non active	None	No	Favourable	NA
30	Lim	2001	Med J Austr	Medicine, General & Int	Q1	Oceania	University	Statin vs non active	Public or non-profit	No	Neutral	NA
31	Grover	2001	Diab Care	Endocrinology	Q1	North America	Hospital	Statin vs non active	Private	No	Favourable	Favourable
32	Tsevat	2001	Am Heart J	Cardiac & Cardiovascula	Q1	North America	Hospital	Statin vs non active	Private	No	NA	Favourable
33	Chau	2001	Hong Kong Med J	NA	NA	Asia	University	Statin vs non active	None	Yes	NA	Neutral
34	van Hout	2001	Eur Heart J	Cardiac & Cardiovascula	Q1	Europe	University	Statin vs non active	None	Yes	NA	Favourable
35	Russell	2001	Can J Clin Pharmacol	NA	NA	North America	Private	Statin vs statin	Private	No	Favourable	Favourable
36	Glasziou	2002	Med J Austr	Medicine, General & Int	Q1	Oceania	University	Statin vs non active	Private	No	NA	Favourable
37	Barry	2002	Ir Med J	NA	NA	Europe	Hospital	Statin vs non active	None	Yes	NA	Favourable

38	Scuffham	2004	Pharmacoeconomics	Pharmacology	Q2	Europe	University	Statin vs non active	Private	Yes	NA	Favourable
39	Pilote	2005	Can J Cardiol	Cardiac & Cardiovascula	Q2	North America	Hospital	Statin vs non active	Public or non-profit	No	Favourable	Favourable
40	Delea	2005	Ann Pharmacother	Pharmacology	Q3	North America	Private	Statin vs non active	Private	Yes	NA	Favourable
41	Nagata-Kobaya	2005	Int J Cardiol	Cardiac & Cardiovascula	Q1	Asia	University	Statin vs non active	None	Yes	Unfavourable	NA
42	Scuffham	2005	Clin Ther	Pharmacology	Q2	Europe	University	Statin vs non active	Private	Yes	NA	Favourable
43	Heart Protecti	2006	BMJ	Medicine, General & In	Q1	Europe	University	Statin vs non active	Private	Yes	NA	Favourable
44	Tonkin	2006	Am Heart J	Cardiac & Cardiovascula	Q1	Oceania	University	Statin vs non active	Public or non-profit	No	NA	Favourable
45	Kohli	2006	Pharmacoeconomics	Pharmacology	Q2	North America	Private	Statin vs statin	Private	No	Favourable	Favourable
46	Fernández de	2006	An Med Interna	NA	NA	Europe	Private	Statin vs non active	Private	Yes	Favourable	NA
47	Walshe	2006	Ir Med J	NA	NA	Europe	Hospital	Statin vs statin	None	Yes	Neutral	NA
48	Lindgren	2007	Eur Heart J	Cardiac & Cardiovascula	Q1	Europe	Private	Statin vs statin	Private	Yes	NA	Favourable
49	Ward	2007	Health Technol Assess	Health Care Sciences &	Q1	Europe	University	Statin vs statin	Public or non-profit	Yes	Neutral	Neutral
50	Raikou	2007	Diabetologia	Endocrinology	Q1	Europe	University	Statin vs non active	Private	Yes	Favourable	NA
51	Lafuma	2008	Arch Cardiovasc Dis	Cardiac & Cardiovascula	Q3	Europe	Private	Statin vs non active	Private	Yes	Favourable	NA
52	Peura	2008	Curr Med Res Opin	Medicine, General & In	Q1	Europe	University	Statin vs statin	Private	Yes	Favourable	Favourable
53	Pinto	2008	Value Health	Health Care Sciences &	Q1	Europe	University	Statin vs statin	Private	Yes	Favourable	NA
54	Ramsey	2008	Pharmacoeconomics	Pharmacology	Q2	North America	University	Statin vs non active	Private	Yes	Favourable	NA
55	Newman	2008	Prev Cardiol.	NA	NA	North America	University	Statin vs statin	None	Yes	Favourable	NA
56	Alonso	2008	Rev Esp Cardiol	Cardiac & Cardiovascula	Q2	Europe	Hospital	Statin vs non active	Private	Yes	Favourable	NA
57	Heart Protecti	2009	Circ Cardiovasc Qual C	NA	NA	North America	Hospital	Statin vs non active	Private	No	NA	Favourable
58	Kongnakorn	2009	Value Health	Health Care Sciences &	Q1	North America	Private	Statin vs non active	Private	Yes	NA	Favourable
59	Ara	2009	Health Technol Assess	Health Care Sciences &	Q1	Europe	University	Statin vs statin	Public or non-profit	Yes	NA	Favourable
60	Taylor	2009	Eur J Health Econ	Health Policy & Services	Q2	Europe	Private	Statin vs statin	Private	Yes	NA	Favourable
61	Wagner	2009	Can J Clin Pharmacol	NA	NA	North America	Private	Statin vs statin	Private	Yes	NA	Favourable
62	Wagner	2009	Can J Cardiol	Cardiac & Cardiovascula	Q2	North America	Private	Statin vs statin	Private	Yes	NA	Favourable
63	Lindgren	2009	Pharmacoeconomics	Pharmacology	Q2	Europe	Private	Statin vs non active	Private	Yes	Favourable	NA
64	Annemans	2010	Clin Drug Investig	Pharmacology	Q3	Europe	University	Statin vs non active	Private	Yes	Favourable	NA
65	Ohsfeldt	2010	J Med Econ	NA	NA	North America	University	Statin vs non active	Private	Yes	Favourable	NA
66	Soini	2010	Curr Med Res Opin	Medicine, General & In	Q1	Europe	Private	Statin vs statin	Private	Yes	NA	Favourable
67	Slejko	2010	Curr Med Res Opin	Medicine, General & In	Q1	North America	University	Statin vs non active	None	Yes	Favourable	NA
68	Nherera	2010	Curr Med Res Opin	Medicine, General & In	Q1	Europe	Primary care	Statin vs statin	Public or non-profit	Yes	Favourable	NA
69	MacDonald	2010	J Am Osteopath Assoc	NA	NA	North America	Hospital	Statin vs non active	None	Yes	Favourable	NA
70	Reckless	2010	Value Health	Health Care Sciences &	Q1	Europe	University	Statin vs statin	Private	Yes	NA	Favourable
71	Rosen	2010	Pharmacoeconomics	Pharmacology	Q2	North America	Private	Statin vs statin	Private	Yes	NA	Favourable
72	Arrospide	2011	Rev Neurol	Clinical Neurology	Q3	Europe	Hospital	Statin vs non active	Private	Yes	NA	Favourable
73	Ara	2011	Eur J Cardiovasc Prev	NA	NA	Europe	University	Statin vs statin	Public or non-profit	Yes	NA	Favourable
74	Choudhry	2011	J Am Coll Cardiol	Cardiac & Cardiovascula	Q1	North America	Hospital	Statin vs non active	Private	Yes	Favourable	NA
75	Michailov	2011	Eur J Health Econ	Health Policy & Services	Q2	Europe	Private	Statin vs statin	Private	Yes	NA	Favourable
