# Supplementary material for: When Are Statins Cost-Effective in Cardiovascular Prevention? A Systematic Review of Sponsorship Bias and Conclusions in Economic Evaluations of Statins
Source: PLoS One. 2013 Jul 8;8(7):e69462. doi: 10.1371/journal.pone.0069462 (PMC3704635; doi:10.1371/journal.pone.0069462)
Supplement: Table S4 — Base case ICERs for Selected Articles. (PDF) [file pone.0069462.s004.pdf]

Additional file_4: Base case ICERs for selected articles

No.	Author	Year	Assessment	Prevention	Sponsorship	Outcome measure	Currency (year)	ICER	ICER_US$ corrected
1	Goldman	1991	Statin vs non active	Secondary	Public or non-profit	Life Years Gained	$ USA (1989)	0	0
1	Goldman	1991	Statin vs non active	Secondary	Public or non-profit	Life Years Gained	$ USA (1989)	0	0
1	Goldman	1991	Statin vs non active	Secondary	Public or non-profit	Life Years Gained	$ USA (1989)	1600	1600
1	Goldman	1991	Statin vs non active	Secondary	Public or non-profit	Life Years Gained	$ USA (1989)	3500	3500
1	Goldman	1991	Statin vs non active	Secondary	Public or non-profit	Life Years Gained	$ USA (1989)	4500	4500
1	Goldman	1991	Statin vs non active	Secondary	Public or non-profit	Life Years Gained	$ USA (1989)	8100	8100
1	Goldman	1991	Statin vs statin	Secondary	Public or non-profit	Life Years Gained	$ USA (1989)	8600	8600
1	Goldman	1991	Statin vs non active	Secondary	Public or non-profit	Life Years Gained	$ USA (1989)	10000	10000
1	Goldman	1991	Statin vs non active	Secondary	Public or non-profit	Life Years Gained	$ USA (1989)	12000	12000
1	Goldman	1991	Statin vs non active	Primary	Public or non-profit	Life Years Gained	$ USA (1989)	13000	13000
1	Goldman	1991	Statin vs statin	Secondary	Public or non-profit	Life Years Gained	$ USA (1989)	14000	14000
1	Goldman	1991	Statin vs non active	Primary	Public or non-profit	Life Years Gained	$ USA (1989)	15000	15000
1	Goldman	1991	Statin vs non active	Secondary	Public or non-profit	Life Years Gained	$ USA (1989)	15000	15000
1	Goldman	1991	Statin vs non active	Secondary	Public or non-profit	Life Years Gained	$ USA (1989)	16000	16000
1	Goldman	1991	Statin vs statin	Secondary	Public or non-profit	Life Years Gained	$ USA (1989)	17000	17000
1	Goldman	1991	Statin vs non active	Primary	Public or non-profit	Life Years Gained	$ USA (1989)	17000	17000
1	Goldman	1991	Statin vs non active	Secondary	Public or non-profit	Life Years Gained	$ USA (1989)	17000	17000
1	Goldman	1991	Statin vs non active	Primary	Public or non-profit	Life Years Gained	$ USA (1989)	18000	18000
1	Goldman	1991	Statin vs non active	Secondary	Public or non-profit	Life Years Gained	$ USA (1989)	19000	19000
1	Goldman	1991	Statin vs non active	Primary	Public or non-profit	Life Years Gained	$ USA (1989)	20000	20000
1	Goldman	1991	Statin vs non active	Primary	Public or non-profit	Life Years Gained	$ USA (1989)	20000	20000
1	Goldman	1991	Statin vs non active	Primary	Public or non-profit	Life Years Gained	$ USA (1989)	21000	21000
1	Goldman	1991	Statin vs non active	Primary	Public or non-profit	Life Years Gained	$ USA (1989)	22000	22000
1	Goldman	1991	Statin vs non active	Primary	Public or non-profit	Life Years Gained	$ USA (1989)	22000	22000
1	Goldman	1991	Statin vs non active	Primary	Public or non-profit	Life Years Gained	$ USA (1989)	23000	23000
1	Goldman	1991	Statin vs non active	Secondary	Public or non-profit	Life Years Gained	$ USA (1989)	23000	23000
1	Goldman	1991	Statin vs non active	Primary	Public or non-profit	Life Years Gained	$ USA (1989)	24000	24000
1	Goldman	1991	Statin vs non active	Primary	Public or non-profit	Life Years Gained	$ USA (1989)	24000	24000
1	Goldman	1991	Statin vs non active	Primary	Public or non-profit	Life Years Gained	$ USA (1989)	24000	24000
1	Goldman	1991	Statin vs non active	Primary	Public or non-profit	Life Years Gained	$ USA (1989)	24000	24000
1	Goldman	1991	Statin vs non active	Primary	Public or non-profit	Life Years Gained	$ USA (1989)	24000	24000
1	Goldman	1991	Statin vs non active	Primary	Public or non-profit	Life Years Gained	$ USA (1989)	25000	25000
1	Goldman	1991	Statin vs non active	Secondary	Public or non-profit	Life Years Gained	$ USA (1989)	25000	25000
1	Goldman	1991	Statin vs non active	Primary	Public or non-profit	Life Years Gained	$ USA (1989)	26000	26000
1	Goldman	1991	Statin vs statin	Secondary	Public or non-profit	Life Years Gained	$ USA (1989)	27000	27000
1	Goldman	1991	Statin vs non active	Primary	Public or non-profit	Life Years Gained	$ USA (1989)	27000	27000
1	Goldman	1991	Statin vs non active	Primary	Public or non-profit	Life Years Gained	$ USA (1989)	28000	28000
1	Goldman	1991	Statin vs non active	Primary	Public or non-profit	Life Years Gained	$ USA (1989)	28000	28000
1	Goldman	1991	Statin vs non active	Primary	Public or non-profit	Life Years Gained	$ USA (1989)	28000	28000
1	Goldman	1991	Statin vs statin	Secondary	Public or non-profit	Life Years Gained	$ USA (1989)	29000	29000
1	Goldman	1991	Statin vs statin	Secondary	Public or non-profit	Life Years Gained	$ USA (1989)	29000	29000
1	Goldman	1991	Statin vs non active	Primary	Public or non-profit	Life Years Gained	$ USA (1989)	29000	29000

1	Goldman	1991	Statin vs non active	Primary	Public or non-profit	Life Years Gained	$ USA (1989)	29000	29000
1	Goldman	1991	Statin vs statin	Secondary	Public or non-profit	Life Years Gained	$ USA (1989)	30000	30000
1	Goldman	1991	Statin vs statin	Secondary	Public or non-profit	Life Years Gained	$ USA (1989)	30000	30000
1	Goldman	1991	Statin vs non active	Primary	Public or non-profit	Life Years Gained	$ USA (1989)	30000	30000
1	Goldman	1991	Statin vs non active	Primary	Public or non-profit	Life Years Gained	$ USA (1989)	30000	30000
1	Goldman	1991	Statin vs non active	Primary	Public or non-profit	Life Years Gained	$ USA (1989)	30000	30000
1	Goldman	1991	Statin vs non active	Primary	Public or non-profit	Life Years Gained	$ USA (1989)	30000	30000
1	Goldman	1991	Statin vs non active	Secondary	Public or non-profit	Life Years Gained	$ USA (1989)	30000	30000
1	Goldman	1991	Statin vs non active	Secondary	Public or non-profit	Life Years Gained	$ USA (1989)	30000	30000
1	Goldman	1991	Statin vs non active	Primary	Public or non-profit	Life Years Gained	$ USA (1989)	31000	31000
1	Goldman	1991	Statin vs non active	Primary	Public or non-profit	Life Years Gained	$ USA (1989)	31000	31000
1	Goldman	1991	Statin vs non active	Primary	Public or non-profit	Life Years Gained	$ USA (1989)	32000	32000
1	Goldman	1991	Statin vs non active	Primary	Public or non-profit	Life Years Gained	$ USA (1989)	32000	32000
1	Goldman	1991	Statin vs non active	Primary	Public or non-profit	Life Years Gained	$ USA (1989)	32000	32000
1	Goldman	1991	Statin vs non active	Primary	Public or non-profit	Life Years Gained	$ USA (1989)	33000	33000
1	Goldman	1991	Statin vs non active	Primary	Public or non-profit	Life Years Gained	$ USA (1989)	34000	34000
1	Goldman	1991	Statin vs non active	Primary	Public or non-profit	Life Years Gained	$ USA (1989)	34000	34000
1	Goldman	1991	Statin vs non active	Primary	Public or non-profit	Life Years Gained	$ USA (1989)	34000	34000
1	Goldman	1991	Statin vs non active	Primary	Public or non-profit	Life Years Gained	$ USA (1989)	34000	34000
1	Goldman	1991	Statin vs non active	Primary	Public or non-profit	Life Years Gained	$ USA (1989)	35000	35000
1	Goldman	1991	Statin vs non active	Primary	Public or non-profit	Life Years Gained	$ USA (1989)	36000	36000
1	Goldman	1991	Statin vs non active	Primary	Public or non-profit	Life Years Gained	$ USA (1989)	36000	36000
1	Goldman	1991	Statin vs non active	Secondary	Public or non-profit	Life Years Gained	$ USA (1989)	36000	36000
1	Goldman	1991	Statin vs non active	Primary	Public or non-profit	Life Years Gained	$ USA (1989)	37000	37000
1	Goldman	1991	Statin vs non active	Primary	Public or non-profit	Life Years Gained	$ USA (1989)	37000	37000
1	Goldman	1991	Statin vs non active	Primary	Public or non-profit	Life Years Gained	$ USA (1989)	37000	37000
1	Goldman	1991	Statin vs non active	Primary	Public or non-profit	Life Years Gained	$ USA (1989)	37000	37000
1	Goldman	1991	Statin vs non active	Primary	Public or non-profit	Life Years Gained	$ USA (1989)	37000	37000
1	Goldman	1991	Statin vs non active	Primary	Public or non-profit	Life Years Gained	$ USA (1989)	37000	37000
1	Goldman	1991	Statin vs non active	Primary	Public or non-profit	Life Years Gained	$ USA (1989)	37000	37000
1	Goldman	1991	Statin vs statin	Secondary	Public or non-profit	Life Years Gained	$ USA (1989)	38000	38000
1	Goldman	1991	Statin vs non active	Primary	Public or non-profit	Life Years Gained	$ USA (1989)	38000	38000
1	Goldman	1991	Statin vs non active	Secondary	Public or non-profit	Life Years Gained	$ USA (1989)	38000	38000
1	Goldman	1991	Statin vs non active	Primary	Public or non-profit	Life Years Gained	$ USA (1989)	39000	39000
1	Goldman	1991	Statin vs non active	Primary	Public or non-profit	Life Years Gained	$ USA (1989)	39000	39000
1	Goldman	1991	Statin vs non active	Primary	Public or non-profit	Life Years Gained	$ USA (1989)	39000	39000
1	Goldman	1991	Statin vs non active	Primary	Public or non-profit	Life Years Gained	$ USA (1989)	39000	39000
1	Goldman	1991	Statin vs non active	Primary	Public or non-profit	Life Years Gained	$ USA (1989)	40000	40000
1	Goldman	1991	Statin vs non active	Primary	Public or non-profit	Life Years Gained	$ USA (1989)	41000	41000
1	Goldman	1991	Statin vs non active	Primary	Public or non-profit	Life Years Gained	$ USA (1989)	41000	41000
1	Goldman	1991	Statin vs non active	Primary	Public or non-profit	Life Years Gained	$ USA (1989)	41000	41000
1	Goldman	1991	Statin vs non active	Primary	Public or non-profit	Life Years Gained	$ USA (1989)	41000	41000
1	Goldman	1991	Statin vs non active	Primary	Public or non-profit	Life Years Gained	$ USA (1989)	42000	42000

1	Goldman	1991	Statin vs non active	Primary	Public or non-profit	Life Years Gained	$ USA (1989)	42000	42000
1	Goldman	1991	Statin vs non active	Primary	Public or non-profit	Life Years Gained	$ USA (1989)	42000	42000
1	Goldman	1991	Statin vs non active	Primary	Public or non-profit	Life Years Gained	$ USA (1989)	42000	42000
1	Goldman	1991	Statin vs non active	Primary	Public or non-profit	Life Years Gained	$ USA (1989)	43000	43000
1	Goldman	1991	Statin vs non active	Primary	Public or non-profit	Life Years Gained	$ USA (1989)	43000	43000
1	Goldman	1991	Statin vs non active	Primary	Public or non-profit	Life Years Gained	$ USA (1989)	43000	43000
1	Goldman	1991	Statin vs non active	Primary	Public or non-profit	Life Years Gained	$ USA (1989)	43000	43000
1	Goldman	1991	Statin vs non active	Primary	Public or non-profit	Life Years Gained	$ USA (1989)	43000	43000
1	Goldman	1991	Statin vs non active	Primary	Public or non-profit	Life Years Gained	$ USA (1989)	44000	44000
1	Goldman	1991	Statin vs non active	Primary	Public or non-profit	Life Years Gained	$ USA (1989)	44000	44000
1	Goldman	1991	Statin vs statin	Secondary	Public or non-profit	Life Years Gained	$ USA (1989)	45000	45000
1	Goldman	1991	Statin vs non active	Primary	Public or non-profit	Life Years Gained	$ USA (1989)	45000	45000
1	Goldman	1991	Statin vs non active	Primary	Public or non-profit	Life Years Gained	$ USA (1989)	45000	45000
1	Goldman	1991	Statin vs non active	Primary	Public or non-profit	Life Years Gained	$ USA (1989)	46000	46000
1	Goldman	1991	Statin vs non active	Primary	Public or non-profit	Life Years Gained	$ USA (1989)	47000	47000
1	Goldman	1991	Statin vs non active	Primary	Public or non-profit	Life Years Gained	$ USA (1989)	47000	47000
1	Goldman	1991	Statin vs statin	Secondary	Public or non-profit	Life Years Gained	$ USA (1989)	48000	48000
1	Goldman	1991	Statin vs non active	Primary	Public or non-profit	Life Years Gained	$ USA (1989)	48000	48000
1	Goldman	1991	Statin vs non active	Primary	Public or non-profit	Life Years Gained	$ USA (1989)	48000	48000
1	Goldman	1991	Statin vs non active	Primary	Public or non-profit	Life Years Gained	$ USA (1989)	48000	48000
1	Goldman	1991	Statin vs statin	Secondary	Public or non-profit	Life Years Gained	$ USA (1989)	49000	49000
1	Goldman	1991	Statin vs non active	Primary	Public or non-profit	Life Years Gained	$ USA (1989)	49000	49000
1	Goldman	1991	Statin vs non active	Primary	Public or non-profit	Life Years Gained	$ USA (1989)	49000	49000
1	Goldman	1991	Statin vs non active	Primary	Public or non-profit	Life Years Gained	$ USA (1989)	49000	49000
1	Goldman	1991	Statin vs non active	Primary	Public or non-profit	Life Years Gained	$ USA (1989)	49000	49000
1	Goldman	1991	Statin vs non active	Primary	Public or non-profit	Life Years Gained	$ USA (1989)	49000	49000
1	Goldman	1991	Statin vs non active	Primary	Public or non-profit	Life Years Gained	$ USA (1989)	50000	50000
1	Goldman	1991	Statin vs non active	Primary	Public or non-profit	Life Years Gained	$ USA (1989)	50000	50000
1	Goldman	1991	Statin vs non active	Primary	Public or non-profit	Life Years Gained	$ USA (1989)	50000	50000
1	Goldman	1991	Statin vs non active	Primary	Public or non-profit	Life Years Gained	$ USA (1989)	52000	52000
1	Goldman	1991	Statin vs non active	Primary	Public or non-profit	Life Years Gained	$ USA (1989)	52000	52000
1	Goldman	1991	Statin vs non active	Primary	Public or non-profit	Life Years Gained	$ USA (1989)	52000	52000
1	Goldman	1991	Statin vs non active	Primary	Public or non-profit	Life Years Gained	$ USA (1989)	52000	52000
1	Goldman	1991	Statin vs non active	Primary	Public or non-profit	Life Years Gained	$ USA (1989)	52000	52000
1	Goldman	1991	Statin vs non active	Primary	Public or non-profit	Life Years Gained	$ USA (1989)	52000	52000
1	Goldman	1991	Statin vs statin	Secondary	Public or non-profit	Life Years Gained	$ USA (1989)	53000	53000
1	Goldman	1991	Statin vs non active	Primary	Public or non-profit	Life Years Gained	$ USA (1989)	53000	53000
1	Goldman	1991	Statin vs non active	Primary	Public or non-profit	Life Years Gained	$ USA (1989)	53000	53000
1	Goldman	1991	Statin vs non active	Primary	Public or non-profit	Life Years Gained	$ USA (1989)	54000	54000
1	Goldman	1991	Statin vs non active	Primary	Public or non-profit	Life Years Gained	$ USA (1989)	55000	55000
1	Goldman	1991	Statin vs non active	Primary	Public or non-profit	Life Years Gained	$ USA (1989)	55000	55000
1	Goldman	1991	Statin vs non active	Primary	Public or non-profit	Life Years Gained	$ USA (1989)	56000	56000
1	Goldman	1991	Statin vs statin	Secondary	Public or non-profit	Life Years Gained	$ USA (1989)	57000	57000

1	Goldman	1991	Statin vs non active	Primary	Public or non-profit	Life Years Gained	$ USA (1989)	57000	57000
1	Goldman	1991	Statin vs non active	Primary	Public or non-profit	Life Years Gained	$ USA (1989)	57000	57000
1	Goldman	1991	Statin vs non active	Primary	Public or non-profit	Life Years Gained	$ USA (1989)	57000	57000
1	Goldman	1991	Statin vs non active	Primary	Public or non-profit	Life Years Gained	$ USA (1989)	57000	57000
1	Goldman	1991	Statin vs statin	Secondary	Public or non-profit	Life Years Gained	$ USA (1989)	58000	58000
1	Goldman	1991	Statin vs non active	Primary	Public or non-profit	Life Years Gained	$ USA (1989)	58000	58000
1	Goldman	1991	Statin vs non active	Primary	Public or non-profit	Life Years Gained	$ USA (1989)	58000	58000
1	Goldman	1991	Statin vs non active	Primary	Public or non-profit	Life Years Gained	$ USA (1989)	58000	58000
1	Goldman	1991	Statin vs non active	Primary	Public or non-profit	Life Years Gained	$ USA (1989)	58000	58000
1	Goldman	1991	Statin vs non active	Primary	Public or non-profit	Life Years Gained	$ USA (1989)	59000	59000
1	Goldman	1991	Statin vs non active	Primary	Public or non-profit	Life Years Gained	$ USA (1989)	61000	61000
1	Goldman	1991	Statin vs non active	Primary	Public or non-profit	Life Years Gained	$ USA (1989)	61000	61000
1	Goldman	1991	Statin vs non active	Primary	Public or non-profit	Life Years Gained	$ USA (1989)	61000	61000
1	Goldman	1991	Statin vs statin	Secondary	Public or non-profit	Life Years Gained	$ USA (1989)	62000	62000
1	Goldman	1991	Statin vs non active	Primary	Public or non-profit	Life Years Gained	$ USA (1989)	62000	62000
1	Goldman	1991	Statin vs non active	Primary	Public or non-profit	Life Years Gained	$ USA (1989)	62000	62000
1	Goldman	1991	Statin vs non active	Primary	Public or non-profit	Life Years Gained	$ USA (1989)	62000	62000
1	Goldman	1991	Statin vs non active	Primary	Public or non-profit	Life Years Gained	$ USA (1989)	63000	63000
1	Goldman	1991	Statin vs non active	Primary	Public or non-profit	Life Years Gained	$ USA (1989)	63000	63000
1	Goldman	1991	Statin vs non active	Primary	Public or non-profit	Life Years Gained	$ USA (1989)	63000	63000
1	Goldman	1991	Statin vs non active	Primary	Public or non-profit	Life Years Gained	$ USA (1989)	63000	63000
1	Goldman	1991	Statin vs non active	Primary	Public or non-profit	Life Years Gained	$ USA (1989)	66000	66000
1	Goldman	1991	Statin vs non active	Primary	Public or non-profit	Life Years Gained	$ USA (1989)	66000	66000
1	Goldman	1991	Statin vs non active	Primary	Public or non-profit	Life Years Gained	$ USA (1989)	67000	67000
1	Goldman	1991	Statin vs non active	Primary	Public or non-profit	Life Years Gained	$ USA (1989)	68000	68000
1	Goldman	1991	Statin vs non active	Primary	Public or non-profit	Life Years Gained	$ USA (1989)	68000	68000
1	Goldman	1991	Statin vs non active	Primary	Public or non-profit	Life Years Gained	$ USA (1989)	69000	69000
1	Goldman	1991	Statin vs non active	Primary	Public or non-profit	Life Years Gained	$ USA (1989)	69000	69000
1	Goldman	1991	Statin vs non active	Primary	Public or non-profit	Life Years Gained	$ USA (1989)	69000	69000
1	Goldman	1991	Statin vs non active	Primary	Public or non-profit	Life Years Gained	$ USA (1989)	70000	70000
1	Goldman	1991	Statin vs non active	Primary	Public or non-profit	Life Years Gained	$ USA (1989)	70000	70000
1	Goldman	1991	Statin vs statin	Secondary	Public or non-profit	Life Years Gained	$ USA (1989)	72000	72000
1	Goldman	1991	Statin vs non active	Primary	Public or non-profit	Life Years Gained	$ USA (1989)	72000	72000
1	Goldman	1991	Statin vs non active	Primary	Public or non-profit	Life Years Gained	$ USA (1989)	72000	72000
1	Goldman	1991	Statin vs statin	Secondary	Public or non-profit	Life Years Gained	$ USA (1989)	73000	73000
1	Goldman	1991	Statin vs non active	Primary	Public or non-profit	Life Years Gained	$ USA (1989)	73000	73000
1	Goldman	1991	Statin vs non active	Primary	Public or non-profit	Life Years Gained	$ USA (1989)	73000	73000
1	Goldman	1991	Statin vs non active	Primary	Public or non-profit	Life Years Gained	$ USA (1989)	73000	73000
1	Goldman	1991	Statin vs non active	Primary	Public or non-profit	Life Years Gained	$ USA (1989)	73000	73000
1	Goldman	1991	Statin vs non active	Secondary	Public or non-profit	Life Years Gained	$ USA (1989)	73000	73000
1	Goldman	1991	Statin vs non active	Primary	Public or non-profit	Life Years Gained	$ USA (1989)	74000	74000
1	Goldman	1991	Statin vs non active	Primary	Public or non-profit	Life Years Gained	$ USA (1989)	74000	74000
1	Goldman	1991	Statin vs non active	Primary	Public or non-profit	Life Years Gained	$ USA (1989)	75000	75000

1	Goldman	1991	Statin vs non active	Primary	Public or non-profit	Life Years Gained	$ USA (1989)	75000	75000
1	Goldman	1991	Statin vs non active	Primary	Public or non-profit	Life Years Gained	$ USA (1989)	76000	76000
1	Goldman	1991	Statin vs non active	Primary	Public or non-profit	Life Years Gained	$ USA (1989)	77000	77000
1	Goldman	1991	Statin vs non active	Primary	Public or non-profit	Life Years Gained	$ USA (1989)	77000	77000
1	Goldman	1991	Statin vs non active	Primary	Public or non-profit	Life Years Gained	$ USA (1989)	78000	78000
1	Goldman	1991	Statin vs non active	Primary	Public or non-profit	Life Years Gained	$ USA (1989)	79000	79000
1	Goldman	1991	Statin vs non active	Primary	Public or non-profit	Life Years Gained	$ USA (1989)	80000	80000
1	Goldman	1991	Statin vs non active	Primary	Public or non-profit	Life Years Gained	$ USA (1989)	80000	80000
1	Goldman	1991	Statin vs non active	Primary	Public or non-profit	Life Years Gained	$ USA (1989)	80000	80000
1	Goldman	1991	Statin vs statin	Secondary	Public or non-profit	Life Years Gained	$ USA (1989)	81000	81000
1	Goldman	1991	Statin vs non active	Primary	Public or non-profit	Life Years Gained	$ USA (1989)	81000	81000
1	Goldman	1991	Statin vs non active	Primary	Public or non-profit	Life Years Gained	$ USA (1989)	81000	81000
1	Goldman	1991	Statin vs non active	Primary	Public or non-profit	Life Years Gained	$ USA (1989)	81000	81000
1	Goldman	1991	Statin vs non active	Primary	Public or non-profit	Life Years Gained	$ USA (1989)	81000	81000
1	Goldman	1991	Statin vs non active	Primary	Public or non-profit	Life Years Gained	$ USA (1989)	81000	81000
1	Goldman	1991	Statin vs non active	Primary	Public or non-profit	Life Years Gained	$ USA (1989)	82000	82000
1	Goldman	1991	Statin vs non active	Primary	Public or non-profit	Life Years Gained	$ USA (1989)	82000	82000
1	Goldman	1991	Statin vs statin	Secondary	Public or non-profit	Life Years Gained	$ USA (1989)	83000	83000
1	Goldman	1991	Statin vs non active	Primary	Public or non-profit	Life Years Gained	$ USA (1989)	83000	83000
1	Goldman	1991	Statin vs non active	Primary	Public or non-profit	Life Years Gained	$ USA (1989)	83000	83000
1	Goldman	1991	Statin vs non active	Primary	Public or non-profit	Life Years Gained	$ USA (1989)	83000	83000
1	Goldman	1991	Statin vs statin	Secondary	Public or non-profit	Life Years Gained	$ USA (1989)	84000	84000
1	Goldman	1991	Statin vs non active	Primary	Public or non-profit	Life Years Gained	$ USA (1989)	84000	84000
1	Goldman	1991	Statin vs non active	Primary	Public or non-profit	Life Years Gained	$ USA (1989)	85000	85000
1	Goldman	1991	Statin vs non active	Primary	Public or non-profit	Life Years Gained	$ USA (1989)	85000	85000
1	Goldman	1991	Statin vs non active	Primary	Public or non-profit	Life Years Gained	$ USA (1989)	85000	85000
1	Goldman	1991	Statin vs non active	Primary	Public or non-profit	Life Years Gained	$ USA (1989)	86000	86000
1	Goldman	1991	Statin vs non active	Primary	Public or non-profit	Life Years Gained	$ USA (1989)	87000	87000
1	Goldman	1991	Statin vs non active	Primary	Public or non-profit	Life Years Gained	$ USA (1989)	88000	88000
1	Goldman	1991	Statin vs non active	Primary	Public or non-profit	Life Years Gained	$ USA (1989)	88000	88000
1	Goldman	1991	Statin vs non active	Primary	Public or non-profit	Life Years Gained	$ USA (1989)	89000	89000
1	Goldman	1991	Statin vs non active	Primary	Public or non-profit	Life Years Gained	$ USA (1989)	89000	89000
1	Goldman	1991	Statin vs non active	Primary	Public or non-profit	Life Years Gained	$ USA (1989)	89000	89000
1	Goldman	1991	Statin vs non active	Primary	Public or non-profit	Life Years Gained	$ USA (1989)	89000	89000
1	Goldman	1991	Statin vs non active	Primary	Public or non-profit	Life Years Gained	$ USA (1989)	91000	91000
1	Goldman	1991	Statin vs non active	Primary	Public or non-profit	Life Years Gained	$ USA (1989)	91000	91000
1	Goldman	1991	Statin vs non active	Primary	Public or non-profit	Life Years Gained	$ USA (1989)	92000	92000
1	Goldman	1991	Statin vs non active	Primary	Public or non-profit	Life Years Gained	$ USA (1989)	92000	92000
1	Goldman	1991	Statin vs statin	Secondary	Public or non-profit	Life Years Gained	$ USA (1989)	93000	93000
1	Goldman	1991	Statin vs non active	Primary	Public or non-profit	Life Years Gained	$ USA (1989)	93000	93000
1	Goldman	1991	Statin vs non active	Primary	Public or non-profit	Life Years Gained	$ USA (1989)	93000	93000
1	Goldman	1991	Statin vs non active	Primary	Public or non-profit	Life Years Gained	$ USA (1989)	95000	95000
1	Goldman	1991	Statin vs non active	Primary	Public or non-profit	Life Years Gained	$ USA (1989)	96000	96000

1	Goldman	1991	Statin vs non active	Primary	Public or non-profit	Life Years Gained	$ USA (1989)	96000	96000
1	Goldman	1991	Statin vs non active	Primary	Public or non-profit	Life Years Gained	$ USA (1989)	98000	98000
1	Goldman	1991	Statin vs non active	Primary	Public or non-profit	Life Years Gained	$ USA (1989)	98000	98000
1	Goldman	1991	Statin vs non active	Primary	Public or non-profit	Life Years Gained	$ USA (1989)	98000	98000
1	Goldman	1991	Statin vs non active	Primary	Public or non-profit	Life Years Gained	$ USA (1989)	98000	98000
1	Goldman	1991	Statin vs non active	Primary	Public or non-profit	Life Years Gained	$ USA (1989)	99000	99000
1	Goldman	1991	Statin vs statin	Secondary	Public or non-profit	Life Years Gained	$ USA (1989)	100000	100000
1	Goldman	1991	Statin vs non active	Primary	Public or non-profit	Life Years Gained	$ USA (1989)	100000	100000
1	Goldman	1991	Statin vs non active	Primary	Public or non-profit	Life Years Gained	$ USA (1989)	100000	100000
1	Goldman	1991	Statin vs non active	Primary	Public or non-profit	Life Years Gained	$ USA (1989)	100000	100000
1	Goldman	1991	Statin vs non active	Primary	Public or non-profit	Life Years Gained	$ USA (1989)	100000	100000
1	Goldman	1991	Statin vs non active	Primary	Public or non-profit	Life Years Gained	$ USA (1989)	100000	100000
1	Goldman	1991	Statin vs non active	Primary	Public or non-profit	Life Years Gained	$ USA (1989)	105000	105000
1	Goldman	1991	Statin vs non active	Primary	Public or non-profit	Life Years Gained	$ USA (1989)	106000	106000
1	Goldman	1991	Statin vs non active	Primary	Public or non-profit	Life Years Gained	$ USA (1989)	110000	110000
1	Goldman	1991	Statin vs non active	Primary	Public or non-profit	Life Years Gained	$ USA (1989)	110000	110000
1	Goldman	1991	Statin vs non active	Primary	Public or non-profit	Life Years Gained	$ USA (1989)	110000	110000
1	Goldman	1991	Statin vs non active	Primary	Public or non-profit	Life Years Gained	$ USA (1989)	110000	110000
1	Goldman	1991	Statin vs non active	Primary	Public or non-profit	Life Years Gained	$ USA (1989)	110000	110000
1	Goldman	1991	Statin vs non active	Primary	Public or non-profit	Life Years Gained	$ USA (1989)	110000	110000
1	Goldman	1991	Statin vs non active	Primary	Public or non-profit	Life Years Gained	$ USA (1989)	110000	110000
1	Goldman	1991	Statin vs non active	Primary	Public or non-profit	Life Years Gained	$ USA (1989)	110000	110000
1	Goldman	1991	Statin vs non active	Primary	Public or non-profit	Life Years Gained	$ USA (1989)	110000	110000
1	Goldman	1991	Statin vs non active	Primary	Public or non-profit	Life Years Gained	$ USA (1989)	110000	110000
1	Goldman	1991	Statin vs statin	Secondary	Public or non-profit	Life Years Gained	$ USA (1989)	120000	120000
1	Goldman	1991	Statin vs statin	Secondary	Public or non-profit	Life Years Gained	$ USA (1989)	120000	120000
1	Goldman	1991	Statin vs non active	Primary	Public or non-profit	Life Years Gained	$ USA (1989)	120000	120000
1	Goldman	1991	Statin vs non active	Primary	Public or non-profit	Life Years Gained	$ USA (1989)	120000	120000
1	Goldman	1991	Statin vs non active	Primary	Public or non-profit	Life Years Gained	$ USA (1989)	120000	120000
1	Goldman	1991	Statin vs non active	Primary	Public or non-profit	Life Years Gained	$ USA (1989)	120000	120000
1	Goldman	1991	Statin vs non active	Primary	Public or non-profit	Life Years Gained	$ USA (1989)	120000	120000
1	Goldman	1991	Statin vs statin	Secondary	Public or non-profit	Life Years Gained	$ USA (1989)	130000	130000
1	Goldman	1991	Statin vs statin	Secondary	Public or non-profit	Life Years Gained	$ USA (1989)	130000	130000
1	Goldman	1991	Statin vs non active	Primary	Public or non-profit	Life Years Gained	$ USA (1989)	130000	130000
1	Goldman	1991	Statin vs non active	Primary	Public or non-profit	Life Years Gained	$ USA (1989)	130000	130000
1	Goldman	1991	Statin vs non active	Primary	Public or non-profit	Life Years Gained	$ USA (1989)	130000	130000
1	Goldman	1991	Statin vs non active	Primary	Public or non-profit	Life Years Gained	$ USA (1989)	130000	130000
1	Goldman	1991	Statin vs non active	Primary	Public or non-profit	Life Years Gained	$ USA (1989)	130000	130000
1	Goldman	1991	Statin vs non active	Primary	Public or non-profit	Life Years Gained	$ USA (1989)	130000	130000
1	Goldman	1991	Statin vs non active	Primary	Public or non-profit	Life Years Gained	$ USA (1989)	130000	130000
1	Goldman	1991	Statin vs non active	Primary	Public or non-profit	Life Years Gained	$ USA (1989)	140000	140000
1	Goldman	1991	Statin vs non active	Primary	Public or non-profit	Life Years Gained	$ USA (1989)	140000	140000
1	Goldman	1991	Statin vs non active	Primary	Public or non-profit	Life Years Gained	$ USA (1989)	140000	140000

1	Goldman	1991	Statin vs non active	Primary	Public or non-profit	Life Years Gained	$ USA (1989)	140000	140000
1	Goldman	1991	Statin vs non active	Primary	Public or non-profit	Life Years Gained	$ USA (1989)	140000	140000
1	Goldman	1991	Statin vs non active	Primary	Public or non-profit	Life Years Gained	$ USA (1989)	140000	140000
1	Goldman	1991	Statin vs non active	Primary	Public or non-profit	Life Years Gained	$ USA (1989)	140000	140000
1	Goldman	1991	Statin vs statin	Secondary	Public or non-profit	Life Years Gained	$ USA (1989)	150000	150000
1	Goldman	1991	Statin vs non active	Primary	Public or non-profit	Life Years Gained	$ USA (1989)	150000	150000
1	Goldman	1991	Statin vs non active	Primary	Public or non-profit	Life Years Gained	$ USA (1989)	150000	150000
1	Goldman	1991	Statin vs non active	Primary	Public or non-profit	Life Years Gained	$ USA (1989)	150000	150000
1	Goldman	1991	Statin vs non active	Primary	Public or non-profit	Life Years Gained	$ USA (1989)	150000	150000
1	Goldman	1991	Statin vs non active	Primary	Public or non-profit	Life Years Gained	$ USA (1989)	150000	150000
1	Goldman	1991	Statin vs non active	Primary	Public or non-profit	Life Years Gained	$ USA (1989)	160000	160000
1	Goldman	1991	Statin vs non active	Primary	Public or non-profit	Life Years Gained	$ USA (1989)	170000	170000
1	Goldman	1991	Statin vs non active	Primary	Public or non-profit	Life Years Gained	$ USA (1989)	170000	170000
1	Goldman	1991	Statin vs non active	Primary	Public or non-profit	Life Years Gained	$ USA (1989)	170000	170000
1	Goldman	1991	Statin vs non active	Primary	Public or non-profit	Life Years Gained	$ USA (1989)	170000	170000
1	Goldman	1991	Statin vs non active	Primary	Public or non-profit	Life Years Gained	$ USA (1989)	180000	180000
1	Goldman	1991	Statin vs non active	Primary	Public or non-profit	Life Years Gained	$ USA (1989)	180000	180000
1	Goldman	1991	Statin vs non active	Primary	Public or non-profit	Life Years Gained	$ USA (1989)	180000	180000
1	Goldman	1991	Statin vs non active	Primary	Public or non-profit	Life Years Gained	$ USA (1989)	180000	180000
1	Goldman	1991	Statin vs non active	Primary	Public or non-profit	Life Years Gained	$ USA (1989)	190000	190000
1	Goldman	1991	Statin vs non active	Primary	Public or non-profit	Life Years Gained	$ USA (1989)	190000	190000
1	Goldman	1991	Statin vs non active	Primary	Public or non-profit	Life Years Gained	$ USA (1989)	190000	190000
1	Goldman	1991	Statin vs non active	Primary	Public or non-profit	Life Years Gained	$ USA (1989)	190000	190000
1	Goldman	1991	Statin vs non active	Primary	Public or non-profit	Life Years Gained	$ USA (1989)	195000	195000
1	Goldman	1991	Statin vs non active	Primary	Public or non-profit	Life Years Gained	$ USA (1989)	200000	200000
1	Goldman	1991	Statin vs non active	Primary	Public or non-profit	Life Years Gained	$ USA (1989)	200000	200000
1	Goldman	1991	Statin vs non active	Primary	Public or non-profit	Life Years Gained	$ USA (1989)	200000	200000
1	Goldman	1991	Statin vs statin	Secondary	Public or non-profit	Life Years Gained	$ USA (1989)	210000	210000
1	Goldman	1991	Statin vs non active	Primary	Public or non-profit	Life Years Gained	$ USA (1989)	210000	210000
1	Goldman	1991	Statin vs non active	Secondary	Public or non-profit	Life Years Gained	$ USA (1989)	210000	210000
1	Goldman	1991	Statin vs non active	Primary	Public or non-profit	Life Years Gained	$ USA (1989)	220000	220000
1	Goldman	1991	Statin vs non active	Primary	Public or non-profit	Life Years Gained	$ USA (1989)	220000	220000
1	Goldman	1991	Statin vs non active	Primary	Public or non-profit	Life Years Gained	$ USA (1989)	230000	230000
1	Goldman	1991	Statin vs non active	Primary	Public or non-profit	Life Years Gained	$ USA (1989)	230000	230000
1	Goldman	1991	Statin vs non active	Primary	Public or non-profit	Life Years Gained	$ USA (1989)	250000	250000
1	Goldman	1991	Statin vs non active	Primary	Public or non-profit	Life Years Gained	$ USA (1989)	250000	250000
1	Goldman	1991	Statin vs non active	Primary	Public or non-profit	Life Years Gained	$ USA (1989)	260000	260000
1	Goldman	1991	Statin vs non active	Primary	Public or non-profit	Life Years Gained	$ USA (1989)	260000	260000
1	Goldman	1991	Statin vs non active	Primary	Public or non-profit	Life Years Gained	$ USA (1989)	270000	270000
1	Goldman	1991	Statin vs non active	Primary	Public or non-profit	Life Years Gained	$ USA (1989)	280000	280000
1	Goldman	1991	Statin vs non active	Primary	Public or non-profit	Life Years Gained	$ USA (1989)	280000	280000
1	Goldman	1991	Statin vs statin	Secondary	Public or non-profit	Life Years Gained	$ USA (1989)	310000	310000
1	Goldman	1991	Statin vs non active	Primary	Public or non-profit	Life Years Gained	$ USA (1989)	320000	320000

1	Goldman	1991	Statin vs non active	Primary	Public or non-profit	Life Years Gained	$ USA (1989)	330000	330000
1	Goldman	1991	Statin vs non active	Primary	Public or non-profit	Life Years Gained	$ USA (1989)	330000	330000
1	Goldman	1991	Statin vs non active	Primary	Public or non-profit	Life Years Gained	$ USA (1989)	350000	350000
1	Goldman	1991	Statin vs non active	Primary	Public or non-profit	Life Years Gained	$ USA (1989)	360000	360000
1	Goldman	1991	Statin vs non active	Primary	Public or non-profit	Life Years Gained	$ USA (1989)	370000	370000
1	Goldman	1991	Statin vs non active	Primary	Public or non-profit	Life Years Gained	$ USA (1989)	370000	370000
1	Goldman	1991	Statin vs non active	Primary	Public or non-profit	Life Years Gained	$ USA (1989)	390000	390000
1	Goldman	1991	Statin vs non active	Primary	Public or non-profit	Life Years Gained	$ USA (1989)	400000	400000
1	Goldman	1991	Statin vs non active	Primary	Public or non-profit	Life Years Gained	$ USA (1989)	480000	480000
1	Goldman	1991	Statin vs non active	Primary	Public or non-profit	Life Years Gained	$ USA (1989)	480000	480000
1	Goldman	1991	Statin vs non active	Primary	Public or non-profit	Life Years Gained	$ USA (1989)	490000	490000
1	Goldman	1991	Statin vs non active	Primary	Public or non-profit	Life Years Gained	$ USA (1989)	500000	500000
1	Goldman	1991	Statin vs non active	Primary	Public or non-profit	Life Years Gained	$ USA (1989)	530000	530000
1	Goldman	1991	Statin vs non active	Primary	Public or non-profit	Life Years Gained	$ USA (1989)	670000	670000
1	Goldman	1991	Statin vs non active	Primary	Public or non-profit	Life Years Gained	$ USA (1989)	690000	690000
1	Goldman	1991	Statin vs non active	Primary	Public or non-profit	Life Years Gained	$ USA (1989)	710000	710000
1	Goldman	1991	Statin vs non active	Primary	Public or non-profit	Life Years Gained	$ USA (1989)	800000	800000
1	Goldman	1991	Statin vs non active	Primary	Public or non-profit	Life Years Gained	$ USA (1989)	990000	990000
1	Goldman	1991	Statin vs non active	Primary	Public or non-profit	Life Years Gained	$ USA (1989)	1000000	1000000
1	Goldman	1991	Statin vs non active	Primary	Public or non-profit	Life Years Gained	$ USA (1989)	1500000	1500000
2	Hay	1991	Statin vs non active	Primary	Private	Life Years Gained	$ USA (1989)	6000	6000
2	Hay	1991	Statin vs non active	Primary	Private	Life Years Gained	$ USA (1989)	8000	8000
2	Hay	1991	Statin vs non active	Primary	Private	Life Years Gained	$ USA (1989)	9000	9000
2	Hay	1991	Statin vs non active	Primary	Private	Life Years Gained	$ USA (1989)	9000	9000
2	Hay	1991	Statin vs non active	Primary	Private	Life Years Gained	$ USA (1989)	11000	11000
2	Hay	1991	Statin vs non active	Primary	Private	Life Years Gained	$ USA (1989)	12000	12000
2	Hay	1991	Statin vs non active	Primary	Private	Life Years Gained	$ USA (1989)	13000	13000
2	Hay	1991	Statin vs non active	Primary	Private	Life Years Gained	$ USA (1989)	13000	13000
2	Hay	1991	Statin vs non active	Primary	Private	Life Years Gained	$ USA (1989)	14000	14000
2	Hay	1991	Statin vs non active	Primary	Private	Life Years Gained	$ USA (1989)	14000	14000
2	Hay	1991	Statin vs non active	Primary	Private	Life Years Gained	$ USA (1989)	15000	15000
2	Hay	1991	Statin vs non active	Primary	Private	Life Years Gained	$ USA (1989)	18000	18000
2	Hay	1991	Statin vs non active	Primary	Private	Life Years Gained	$ USA (1989)	19000	19000
2	Hay	1991	Statin vs non active	Primary	Private	Life Years Gained	$ USA (1989)	19000	19000
2	Hay	1991	Statin vs non active	Primary	Private	Life Years Gained	$ USA (1989)	19000	19000
2	Hay	1991	Statin vs non active	Primary	Private	Life Years Gained	$ USA (1989)	20000	20000
2	Hay	1991	Statin vs non active	Primary	Private	Life Years Gained	$ USA (1989)	20000	20000
2	Hay	1991	Statin vs non active	Primary	Private	Life Years Gained	$ USA (1989)	21000	21000
2	Hay	1991	Statin vs non active	Primary	Private	Life Years Gained	$ USA (1989)	22000	22000
2	Hay	1991	Statin vs non active	Primary	Private	Life Years Gained	$ USA (1989)	22500	22500
2	Hay	1991	Statin vs non active	Primary	Private	Life Years Gained	$ USA (1989)	23000	23000
2	Hay	1991	Statin vs non active	Primary	Private	Life Years Gained	$ USA (1989)	24000	24000
2	Hay	1991	Statin vs non active	Primary	Private	Life Years Gained	$ USA (1989)	25000	25000

2	Hay	1991	Statin vs non active	Primary	Private	Life Years Gained	$ USA (1989)	25000	25000
2	Hay	1991	Statin vs non active	Primary	Private	Life Years Gained	$ USA (1989)	27000	27000
2	Hay	1991	Statin vs non active	Primary	Private	Life Years Gained	$ USA (1989)	28000	28000
2	Hay	1991	Statin vs non active	Primary	Private	Life Years Gained	$ USA (1989)	29000	29000
2	Hay	1991	Statin vs non active	Primary	Private	Life Years Gained	$ USA (1989)	29000	29000
2	Hay	1991	Statin vs non active	Primary	Private	Life Years Gained	$ USA (1989)	30000	30000
2	Hay	1991	Statin vs non active	Primary	Private	Life Years Gained	$ USA (1989)	31000	31000
2	Hay	1991	Statin vs non active	Primary	Private	Life Years Gained	$ USA (1989)	31000	31000
2	Hay	1991	Statin vs non active	Primary	Private	Life Years Gained	$ USA (1989)	34000	34000
2	Hay	1991	Statin vs non active	Primary	Private	Life Years Gained	$ USA (1989)	34000	34000
2	Hay	1991	Statin vs non active	Primary	Private	Life Years Gained	$ USA (1989)	35000	35000
2	Hay	1991	Statin vs non active	Primary	Private	Life Years Gained	$ USA (1989)	35000	35000
2	Hay	1991	Statin vs non active	Primary	Private	Life Years Gained	$ USA (1989)	36000	36000
2	Hay	1991	Statin vs non active	Primary	Private	Life Years Gained	$ USA (1989)	37000	37000
2	Hay	1991	Statin vs non active	Primary	Private	Life Years Gained	$ USA (1989)	38000	38000
2	Hay	1991	Statin vs non active	Primary	Private	Life Years Gained	$ USA (1989)	40000	40000
2	Hay	1991	Statin vs non active	Primary	Private	Life Years Gained	$ USA (1989)	41000	41000
2	Hay	1991	Statin vs non active	Primary	Private	Life Years Gained	$ USA (1989)	42000	42000
2	Hay	1991	Statin vs non active	Primary	Private	Life Years Gained	$ USA (1989)	43000	43000
2	Hay	1991	Statin vs non active	Primary	Private	Life Years Gained	$ USA (1989)	43000	43000
2	Hay	1991	Statin vs non active	Primary	Private	Life Years Gained	$ USA (1989)	46000	46000
2	Hay	1991	Statin vs non active	Primary	Private	Life Years Gained	$ USA (1989)	46000	46000
2	Hay	1991	Statin vs non active	Primary	Private	Life Years Gained	$ USA (1989)	48000	48000
2	Hay	1991	Statin vs non active	Primary	Private	Life Years Gained	$ USA (1989)	53000	53000
2	Hay	1991	Statin vs non active	Primary	Private	Life Years Gained	$ USA (1989)	53000	53000
2	Hay	1991	Statin vs non active	Primary	Private	Life Years Gained	$ USA (1989)	54000	54000
2	Hay	1991	Statin vs non active	Primary	Private	Life Years Gained	$ USA (1989)	54000	54000
2	Hay	1991	Statin vs non active	Primary	Private	Life Years Gained	$ USA (1989)	57000	57000
2	Hay	1991	Statin vs non active	Primary	Private	Life Years Gained	$ USA (1989)	57000	57000
2	Hay	1991	Statin vs non active	Primary	Private	Life Years Gained	$ USA (1989)	58000	58000
2	Hay	1991	Statin vs non active	Primary	Private	Life Years Gained	$ USA (1989)	58000	58000
2	Hay	1991	Statin vs non active	Primary	Private	Life Years Gained	$ USA (1989)	58000	58000
2	Hay	1991	Statin vs non active	Primary	Private	Life Years Gained	$ USA (1989)	59000	59000
2	Hay	1991	Statin vs non active	Primary	Private	Life Years Gained	$ USA (1989)	66000	66000
2	Hay	1991	Statin vs non active	Primary	Private	Life Years Gained	$ USA (1989)	68000	68000
2	Hay	1991	Statin vs non active	Primary	Private	Life Years Gained	$ USA (1989)	70000	70000
2	Hay	1991	Statin vs non active	Primary	Private	Life Years Gained	$ USA (1989)	75000	75000
2	Hay	1991	Statin vs non active	Primary	Private	Life Years Gained	$ USA (1989)	80000	80000
2	Hay	1991	Statin vs non active	Primary	Private	Life Years Gained	$ USA (1989)	81000	81000
2	Hay	1991	Statin vs non active	Primary	Private	Life Years Gained	$ USA (1989)	81000	81000
2	Hay	1991	Statin vs non active	Primary	Private	Life Years Gained	$ USA (1989)	82000	82000
2	Hay	1991	Statin vs non active	Primary	Private	Life Years Gained	$ USA (1989)	86000	86000
2	Hay	1991	Statin vs non active	Primary	Private	Life Years Gained	$ USA (1989)	89000	89000

2	Hay	1991	Statin vs non active	Primary	Private	Life Years Gained	$ USA (1989)	106000	106000
2	Hay	1991	Statin vs non active	Primary	Private	Life Years Gained	$ USA (1989)	108000	108000
2	Hay	1991	Statin vs non active	Primary	Private	Life Years Gained	$ USA (1989)	110000	110000
2	Hay	1991	Statin vs non active	Primary	Private	Life Years Gained	$ USA (1989)	111000	111000
2	Hay	1991	Statin vs non active	Primary	Private	Life Years Gained	$ USA (1989)	112000	112000
2	Hay	1991	Statin vs non active	Primary	Private	Life Years Gained	$ USA (1989)	120000	120000
2	Hay	1991	Statin vs non active	Primary	Private	Life Years Gained	$ USA (1989)	126000	126000
2	Hay	1991	Statin vs non active	Primary	Private	Life Years Gained	$ USA (1989)	151000	151000
2	Hay	1991	Statin vs non active	Primary	Private	Life Years Gained	$ USA (1989)	154000	154000
2	Hay	1991	Statin vs non active	Primary	Private	Life Years Gained	$ USA (1989)	160000	160000
2	Hay	1991	Statin vs non active	Primary	Private	Life Years Gained	$ USA (1989)	167000	167000
2	Hay	1991	Statin vs non active	Primary	Private	Life Years Gained	$ USA (1989)	204000	204000
2	Hay	1991	Statin vs non active	Primary	Private	Life Years Gained	$ USA (1989)	278000	278000
2	Hay	1991	Statin vs non active	Primary	Private	Life Years Gained	$ USA (1989)	297000	297000
3	Goldman	1993	Statin vs statin	Primary	Public or non-profit	Life Years Gained	$ USA (1989)	2000	2000
3	Goldman	1993	Statin vs statin	Primary	Public or non-profit	Life Years Gained	$ USA (1989)	6000	6000
3	Goldman	1993	Statin vs statin	Primary	Public or non-profit	Life Years Gained	$ USA (1989)	16000	16000
3	Goldman	1993	Statin vs statin	Primary	Public or non-profit	Life Years Gained	$ USA (1989)	30000	30000
3	Goldman	1993	Statin vs statin	Primary	Public or non-profit	Life Years Gained	$ USA (1989)	42000	42000
3	Goldman	1993	Statin vs statin	Primary	Public or non-profit	Life Years Gained	$ USA (1989)	70000	70000
3	Goldman	1993	Statin vs statin	Primary	Public or non-profit	Life Years Gained	$ USA (1989)	70000	70000
3	Goldman	1993	Statin vs statin	Primary	Public or non-profit	Life Years Gained	$ USA (1989)	90000	90000
3	Goldman	1993	Statin vs statin	Primary	Public or non-profit	Life Years Gained	$ USA (1989)	120000	120000
3	Goldman	1993	Statin vs statin	Primary	Public or non-profit	Life Years Gained	$ USA (1989)	150000	150000
3	Goldman	1993	Statin vs statin	Primary	Public or non-profit	Life Years Gained	$ USA (1989)	160000	160000
3	Goldman	1993	Statin vs statin	Primary	Public or non-profit	Life Years Gained	$ USA (1989)	210000	210000
3	Goldman	1993	Statin vs statin	Primary	Public or non-profit	Life Years Gained	$ USA (1989)	320000	320000
3	Goldman	1993	Statin vs statin	Primary	Public or non-profit	Life Years Gained	$ USA (1989)	380000	380000
3	Goldman	1993	Statin vs statin	Primary	Public or non-profit	Life Years Gained	$ USA (1989)	470000	470000
3	Goldman	1993	Statin vs statin	Primary	Public or non-profit	Life Years Gained	$ USA (1989)	720000	720000
4	Martens	1994	Statin vs non active	Primary	Private	Life Years Gained	$ Canadian (1993)	38800	40430
4	Martens	1994	Statin vs non active	Primary	Private	Life Years Gained	$ Canadian (1993)	48300	50330
4	Martens	1994	Statin vs non active	Primary	Private	Life Years Gained	$ Canadian (1993)	53000	55227
4	Martens	1994	Statin vs non active	Primary	Private	Life Years Gained	$ Canadian (1993)	56200	58562
4	Martens	1994	Statin vs statin	Primary	Private	Life Years Gained	$ Canadian (1993)	88200	91906
4	Martens	1994	Statin vs statin	Primary	Private	Life Years Gained	$ Canadian (1993)	198100	206424
4	Martens	1994	Statin vs statin	Primary	Private	Life Years Gained	$ Canadian (1993)	330300	344179
5	Hamilton	1995	Statin vs non active	Primary	Private	Life Years Gained	$ Canadian (1993)	17231	17955
5	Hamilton	1995	Statin vs non active	Primary	Private	Life Years Gained	$ Canadian (1993)	19415	20231
5	Hamilton	1995	Statin vs non active	Primary	Private	Life Years Gained	$ Canadian (1993)	20882	21759
5	Hamilton	1995	Statin vs non active	Primary	Private	Life Years Gained	$ Canadian (1993)	22297	23234
5	Hamilton	1995	Statin vs non active	Primary	Private	Life Years Gained	$ Canadian (1993)	22642	23593
5	Hamilton	1995	Statin vs non active	Primary	Private	Life Years Gained	$ Canadian (1993)	27872	29043

5	Hamilton	1995	Statin vs non active	Primary	Private	Life Years Gained	$ Canadian (1993)	30540	31823
5	Hamilton	1995	Statin vs non active	Primary	Private	Life Years Gained	$ Canadian (1993)	33257	34654
5	Hamilton	1995	Statin vs non active	Primary	Private	Life Years Gained	$ Canadian (1993)	35166	36644
5	Hamilton	1995	Statin vs non active	Primary	Private	Life Years Gained	$ Canadian (1993)	35526	37019
5	Hamilton	1995	Statin vs non active	Primary	Private	Life Years Gained	$ Canadian (1993)	35785	37289
5	Hamilton	1995	Statin vs non active	Primary	Private	Life Years Gained	$ Canadian (1993)	36627	38166
5	Hamilton	1995	Statin vs non active	Primary	Private	Life Years Gained	$ Canadian (1993)	37453	39027
5	Hamilton	1995	Statin vs non active	Primary	Private	Life Years Gained	$ Canadian (1993)	40436	42135
5	Hamilton	1995	Statin vs non active	Primary	Private	Life Years Gained	$ Canadian (1993)	41945	43708
5	Hamilton	1995	Statin vs non active	Primary	Private	Life Years Gained	$ Canadian (1993)	42313	44091
5	Hamilton	1995	Statin vs non active	Primary	Private	Life Years Gained	$ Canadian (1993)	42458	44242
5	Hamilton	1995	Statin vs non active	Primary	Private	Life Years Gained	$ Canadian (1993)	42504	44290
5	Hamilton	1995	Statin vs non active	Primary	Private	Life Years Gained	$ Canadian (1993)	43127	44939
5	Hamilton	1995	Statin vs non active	Primary	Private	Life Years Gained	$ Canadian (1993)	44525	46396
5	Hamilton	1995	Statin vs non active	Primary	Private	Life Years Gained	$ Canadian (1993)	46571	48528
5	Hamilton	1995	Statin vs non active	Primary	Private	Life Years Gained	$ Canadian (1993)	47445	49439
5	Hamilton	1995	Statin vs non active	Primary	Private	Life Years Gained	$ Canadian (1993)	48214	50240
5	Hamilton	1995	Statin vs non active	Primary	Private	Life Years Gained	$ Canadian (1993)	50079	52183
5	Hamilton	1995	Statin vs non active	Primary	Private	Life Years Gained	$ Canadian (1993)	51293	53448
5	Hamilton	1995	Statin vs non active	Primary	Private	Life Years Gained	$ Canadian (1993)	55579	57914
5	Hamilton	1995	Statin vs non active	Primary	Private	Life Years Gained	$ Canadian (1993)	56191	58552
5	Hamilton	1995	Statin vs non active	Primary	Private	Life Years Gained	$ Canadian (1993)	57689	60113
5	Hamilton	1995	Statin vs non active	Primary	Private	Life Years Gained	$ Canadian (1993)	61891	64492
5	Hamilton	1995	Statin vs non active	Primary	Private	Life Years Gained	$ Canadian (1993)	61898	64499
5	Hamilton	1995	Statin vs non active	Primary	Private	Life Years Gained	$ Canadian (1993)	67634	70476
5	Hamilton	1995	Statin vs non active	Primary	Private	Life Years Gained	$ Canadian (1993)	73121	76194
5	Hamilton	1995	Statin vs non active	Primary	Private	Life Years Gained	$ Canadian (1993)	75625	78803
5	Hamilton	1995	Statin vs non active	Primary	Private	Life Years Gained	$ Canadian (1993)	76749	79974
5	Hamilton	1995	Statin vs non active	Primary	Private	Life Years Gained	$ Canadian (1993)	86551	90188
5	Hamilton	1995	Statin vs non active	Primary	Private	Life Years Gained	$ Canadian (1993)	91655	95506
5	Hamilton	1995	Statin vs non active	Primary	Private	Life Years Gained	$ Canadian (1993)	101868	106148
5	Hamilton	1995	Statin vs non active	Primary	Private	Life Years Gained	$ Canadian (1993)	105708	110150
5	Hamilton	1995	Statin vs non active	Primary	Private	Life Years Gained	$ Canadian (1993)	151132	157483
5	Hamilton	1995	Statin vs non active	Primary	Private	Life Years Gained	$ Canadian (1993)	155891	162442
6	Johanesson	1996	Statin vs non active	Primary	Private	Life Years Gained	$ USA (1991)	15000	15000
6	Johanesson	1996	Statin vs non active	Primary	Private	Life Years Gained	$ USA (1991)	51000	51000
7	Jönsson	1996	Statin vs non active	Secondary	Private	Life Years Gained	£ UK (1995)	5502	8878
8	Pharoa	1996	Statin vs non active	Secondary	None	Life Years Gained	£ UK (1995)	32000	51636
8	Pharoa	1996	Statin vs non active	Primary	None	Life Years Gained	£ UK (1995)	147000	237205
9	Ashraf	1996	Statin vs non active	Secondary	Private	Life Years Gained	$ USA (1995)	7124	7124
9	Ashraf	1996	Statin vs non active	Secondary	Private	Life Years Gained	$ USA (1995)	9368	9368
9	Ashraf	1996	Statin vs non active	Secondary	Private	Life Years Gained	$ USA (1995)	12665	12665
10	Riviere	1997	Statin vs non active	Secondary	Private	Life Years Gained	$ Canadian (1995)	9867	10282

11	Caro	1997	Statin vs non active	Primary	Private	Life Years Gained	£ UK (1996)	13995	22583
11	Caro	1997	Statin vs non active	Primary	Private	Life Years Gained	£ UK (1996)	20375	32878
12	Johanesson	1997	Statin vs non active	Secondary	Private	Life Years Gained	$ USA (1995)	0	0
12	Johanesson	1997	Statin vs non active	Secondary	Private	Life Years Gained	$ USA (1995)	0	0
12	Johanesson	1997	Statin vs non active	Secondary	Private	Life Years Gained	$ USA (1995)	0	0
12	Johanesson	1997	Statin vs non active	Secondary	Private	Life Years Gained	$ USA (1995)	0	0
12	Johanesson	1997	Statin vs non active	Secondary	Private	Life Years Gained	$ USA (1995)	0	0
12	Johanesson	1997	Statin vs non active	Secondary	Private	Life Years Gained	$ USA (1995)	0	0
12	Johanesson	1997	Statin vs non active	Secondary	Private	Life Years Gained	$ USA (1995)	1200	1200
12	Johanesson	1997	Statin vs non active	Secondary	Private	Life Years Gained	$ USA (1995)	1600	1600
12	Johanesson	1997	Statin vs non active	Secondary	Private	Life Years Gained	$ USA (1995)	2100	2100
12	Johanesson	1997	Statin vs non active	Secondary	Private	Life Years Gained	$ USA (1995)	3200	3200
12	Johanesson	1997	Statin vs non active	Secondary	Private	Life Years Gained	$ USA (1995)	3800	3800
12	Johanesson	1997	Statin vs non active	Secondary	Private	Life Years Gained	$ USA (1995)	4700	4700
12	Johanesson	1997	Statin vs non active	Secondary	Private	Life Years Gained	$ USA (1995)	4900	4900
12	Johanesson	1997	Statin vs non active	Secondary	Private	Life Years Gained	$ USA (1995)	6200	6200
12	Johanesson	1997	Statin vs non active	Secondary	Private	Life Years Gained	$ USA (1995)	6200	6200
12	Johanesson	1997	Statin vs non active	Secondary	Private	Life Years Gained	$ USA (1995)	8500	8500
12	Johanesson	1997	Statin vs non active	Secondary	Private	Life Years Gained	$ USA (1995)	8600	8600
12	Johanesson	1997	Statin vs non active	Secondary	Private	Life Years Gained	$ USA (1995)	13300	13300
13	Troche	1998	Statin vs non active	Primary	None	Life Years Gained	$ USA (1995)	195000	195000
14	Muls	1998	Statin vs non active	Secondary	Private	Life Years Gained	$ USA (1995)	7124	7124
14	Muls	1998	Statin vs non active	Secondary	Private	Life Years Gained	$ USA (1995)	9368	9368
14	Muls	1998	Statin vs non active	Secondary	Private	Life Years Gained	$ USA (1995)	12665	12665
14	Muls	1998	Statin vs non active	Secondary	Private	Life Years Gained	$ USA (1995)	13274	13274
14	Muls	1998	Statin vs non active	Secondary	Private	Life Years Gained	$ USA (1995)	17792	17792
14	Muls	1998	Statin vs non active	Secondary	Private	Life Years Gained	$ USA (1995)	24359	24359
15	Huse	1998	Statin vs statin	Primary	Private	Life Years Gained	$ USA (1997)	865	865
15	Huse	1998	Statin vs statin	Primary	Private	Life Years Gained	$ USA (1997)	3080	3080
15	Huse	1998	Statin vs statin	Primary	Private	Life Years Gained	$ USA (1997)	3208	3208
15	Huse	1998	Statin vs statin	Primary	Private	Life Years Gained	$ USA (1997)	3315	3315
15	Huse	1998	Statin vs non active	Primary	Private	Life Years Gained	$ USA (1997)	4294	4294
15	Huse	1998	Statin vs statin	Primary	Private	Life Years Gained	$ USA (1997)	4514	4514
15	Huse	1998	Statin vs statin	Secondary	Private	Life Years Gained	$ USA (1997)	4665	4665
15	Huse	1998	Statin vs statin	Secondary	Private	Life Years Gained	$ USA (1997)	5332	5332
15	Huse	1998	Statin vs statin	Secondary	Private	Life Years Gained	$ USA (1997)	5506	5506
15	Huse	1998	Statin vs non active	Primary	Private	Life Years Gained	$ USA (1997)	6081	6081
15	Huse	1998	Statin vs statin	Secondary	Private	Life Years Gained	$ USA (1997)	6122	6122
15	Huse	1998	Statin vs statin	Secondary	Private	Life Years Gained	$ USA (1997)	6169	6169
15	Huse	1998	Statin vs statin	Secondary	Private	Life Years Gained	$ USA (1997)	6587	6587
15	Huse	1998	Statin vs statin	Secondary	Private	Life Years Gained	$ USA (1997)	6746	6746
15	Huse	1998	Statin vs statin	Primary	Private	Life Years Gained	$ USA (1997)	6998	6998
15	Huse	1998	Statin vs statin	Secondary	Private	Life Years Gained	$ USA (1997)	7124	7124

15	Huse	1998	Statin vs non active	Primary	Private	Life Years Gained	$ USA (1997)	7223	7223
15	Huse	1998	Statin vs non active	Primary	Private	Life Years Gained	$ USA (1997)	7276	7276
15	Huse	1998	Statin vs statin	Secondary	Private	Life Years Gained	$ USA (1997)	7415	7415
15	Huse	1998	Statin vs non active	Primary	Private	Life Years Gained	$ USA (1997)	7442	7442
15	Huse	1998	Statin vs statin	Secondary	Private	Life Years Gained	$ USA (1997)	7976	7976
15	Huse	1998	Statin vs statin	Secondary	Private	Life Years Gained	$ USA (1997)	7983	7983
15	Huse	1998	Statin vs statin	Secondary	Private	Life Years Gained	$ USA (1997)	8149	8149
15	Huse	1998	Statin vs non active	Secondary	Private	Life Years Gained	$ USA (1997)	8224	8224
15	Huse	1998	Statin vs non active	Primary	Private	Life Years Gained	$ USA (1997)	8313	8313
15	Huse	1998	Statin vs non active	Secondary	Private	Life Years Gained	$ USA (1997)	8713	8713
15	Huse	1998	Statin vs statin	Primary	Private	Life Years Gained	$ USA (1997)	8889	8889
15	Huse	1998	Statin vs statin	Secondary	Private	Life Years Gained	$ USA (1997)	9118	9118
15	Huse	1998	Statin vs non active	Primary	Private	Life Years Gained	$ USA (1997)	9270	9270
15	Huse	1998	Statin vs non active	Secondary	Private	Life Years Gained	$ USA (1997)	9311	9311
15	Huse	1998	Statin vs non active	Primary	Private	Life Years Gained	$ USA (1997)	9417	9417
15	Huse	1998	Statin vs non active	Primary	Private	Life Years Gained	$ USA (1997)	9454	9454
15	Huse	1998	Statin vs statin	Secondary	Private	Life Years Gained	$ USA (1997)	9522	9522
15	Huse	1998	Statin vs non active	Secondary	Private	Life Years Gained	$ USA (1997)	10170	10170
15	Huse	1998	Statin vs statin	Secondary	Private	Life Years Gained	$ USA (1997)	10429	10429
15	Huse	1998	Statin vs statin	Secondary	Private	Life Years Gained	$ USA (1997)	10466	10466
15	Huse	1998	Statin vs non active	Secondary	Private	Life Years Gained	$ USA (1997)	10574	10574
15	Huse	1998	Statin vs non active	Primary	Private	Life Years Gained	$ USA (1997)	10603	10603
15	Huse	1998	Statin vs non active	Secondary	Private	Life Years Gained	$ USA (1997)	10630	10630
15	Huse	1998	Statin vs statin	Secondary	Private	Life Years Gained	$ USA (1997)	10639	10639
15	Huse	1998	Statin vs non active	Secondary	Private	Life Years Gained	$ USA (1997)	10686	10686
15	Huse	1998	Statin vs non active	Primary	Private	Life Years Gained	$ USA (1997)	10928	10928
15	Huse	1998	Statin vs statin	Secondary	Private	Life Years Gained	$ USA (1997)	10954	10954
15	Huse	1998	Statin vs non active	Secondary	Private	Life Years Gained	$ USA (1997)	11015	11015
15	Huse	1998	Statin vs non active	Primary	Private	Life Years Gained	$ USA (1997)	11053	11053
15	Huse	1998	Statin vs non active	Secondary	Private	Life Years Gained	$ USA (1997)	11142	11142
15	Huse	1998	Statin vs non active	Primary	Private	Life Years Gained	$ USA (1997)	11310	11310
15	Huse	1998	Statin vs non active	Secondary	Private	Life Years Gained	$ USA (1997)	11384	11384
15	Huse	1998	Statin vs non active	Secondary	Private	Life Years Gained	$ USA (1997)	11540	11540
15	Huse	1998	Statin vs non active	Primary	Private	Life Years Gained	$ USA (1997)	11714	11714
15	Huse	1998	Statin vs non active	Secondary	Private	Life Years Gained	$ USA (1997)	11846	11846
15	Huse	1998	Statin vs non active	Secondary	Private	Life Years Gained	$ USA (1997)	11872	11872
15	Huse	1998	Statin vs non active	Secondary	Private	Life Years Gained	$ USA (1997)	11940	11940
15	Huse	1998	Statin vs non active	Primary	Private	Life Years Gained	$ USA (1997)	11988	11988
15	Huse	1998	Statin vs non active	Primary	Private	Life Years Gained	$ USA (1997)	12137	12137
15	Huse	1998	Statin vs non active	Secondary	Private	Life Years Gained	$ USA (1997)	12549	12549
15	Huse	1998	Statin vs non active	Primary	Private	Life Years Gained	$ USA (1997)	12739	12739
15	Huse	1998	Statin vs non active	Secondary	Private	Life Years Gained	$ USA (1997)	12835	12835
15	Huse	1998	Statin vs non active	Secondary	Private	Life Years Gained	$ USA (1997)	12849	12849

15	Huse	1998	Statin vs non active	Secondary	Private	Life Years Gained	$ USA (1997)	13027	13027
15	Huse	1998	Statin vs non active	Secondary	Private	Life Years Gained	$ USA (1997)	13030	13030
15	Huse	1998	Statin vs non active	Primary	Private	Life Years Gained	$ USA (1997)	13064	13064
15	Huse	1998	Statin vs non active	Secondary	Private	Life Years Gained	$ USA (1997)	13129	13129
15	Huse	1998	Statin vs non active	Secondary	Private	Life Years Gained	$ USA (1997)	13320	13320
15	Huse	1998	Statin vs non active	Secondary	Private	Life Years Gained	$ USA (1997)	13334	13334
15	Huse	1998	Statin vs non active	Secondary	Private	Life Years Gained	$ USA (1997)	13338	13338
15	Huse	1998	Statin vs statin	Secondary	Private	Life Years Gained	$ USA (1997)	13491	13491
15	Huse	1998	Statin vs non active	Secondary	Private	Life Years Gained	$ USA (1997)	13523	13523
15	Huse	1998	Statin vs statin	Primary	Private	Life Years Gained	$ USA (1997)	13580	13580
15	Huse	1998	Statin vs non active	Primary	Private	Life Years Gained	$ USA (1997)	13608	13608
15	Huse	1998	Statin vs statin	Secondary	Private	Life Years Gained	$ USA (1997)	13799	13799
15	Huse	1998	Statin vs non active	Secondary	Private	Life Years Gained	$ USA (1997)	13908	13908
15	Huse	1998	Statin vs non active	Primary	Private	Life Years Gained	$ USA (1997)	14353	14353
15	Huse	1998	Statin vs statin	Primary	Private	Life Years Gained	$ USA (1997)	14359	14359
15	Huse	1998	Statin vs non active	Secondary	Private	Life Years Gained	$ USA (1997)	14465	14465
15	Huse	1998	Statin vs non active	Secondary	Private	Life Years Gained	$ USA (1997)	14536	14536
15	Huse	1998	Statin vs non active	Secondary	Private	Life Years Gained	$ USA (1997)	14668	14668
15	Huse	1998	Statin vs non active	Secondary	Private	Life Years Gained	$ USA (1997)	14831	14831
15	Huse	1998	Statin vs non active	Secondary	Private	Life Years Gained	$ USA (1997)	14942	14942
15	Huse	1998	Statin vs non active	Secondary	Private	Life Years Gained	$ USA (1997)	15041	15041
15	Huse	1998	Statin vs non active	Secondary	Private	Life Years Gained	$ USA (1997)	15190	15190
15	Huse	1998	Statin vs non active	Secondary	Private	Life Years Gained	$ USA (1997)	15264	15264
15	Huse	1998	Statin vs non active	Primary	Private	Life Years Gained	$ USA (1997)	15266	15266
15	Huse	1998	Statin vs non active	Primary	Private	Life Years Gained	$ USA (1997)	15331	15331
15	Huse	1998	Statin vs non active	Secondary	Private	Life Years Gained	$ USA (1997)	15770	15770
15	Huse	1998	Statin vs non active	Primary	Private	Life Years Gained	$ USA (1997)	15823	15823
15	Huse	1998	Statin vs non active	Secondary	Private	Life Years Gained	$ USA (1997)	15823	15823
15	Huse	1998	Statin vs statin	Primary	Private	Life Years Gained	$ USA (1997)	15839	15839
15	Huse	1998	Statin vs non active	Secondary	Private	Life Years Gained	$ USA (1997)	15934	15934
15	Huse	1998	Statin vs non active	Secondary	Private	Life Years Gained	$ USA (1997)	15969	15969
15	Huse	1998	Statin vs non active	Secondary	Private	Life Years Gained	$ USA (1997)	15994	15994
15	Huse	1998	Statin vs non active	Secondary	Private	Life Years Gained	$ USA (1997)	16112	16112
15	Huse	1998	Statin vs non active	Secondary	Private	Life Years Gained	$ USA (1997)	16115	16115
15	Huse	1998	Statin vs non active	Secondary	Private	Life Years Gained	$ USA (1997)	16156	16156
15	Huse	1998	Statin vs non active	Primary	Private	Life Years Gained	$ USA (1997)	16339	16339
15	Huse	1998	Statin vs non active	Secondary	Private	Life Years Gained	$ USA (1997)	16351	16351
15	Huse	1998	Statin vs non active	Secondary	Private	Life Years Gained	$ USA (1997)	16400	16400
15	Huse	1998	Statin vs non active	Secondary	Private	Life Years Gained	$ USA (1997)	16409	16409
15	Huse	1998	Statin vs statin	Secondary	Private	Life Years Gained	$ USA (1997)	16611	16611
15	Huse	1998	Statin vs non active	Primary	Private	Life Years Gained	$ USA (1997)	16795	16795
15	Huse	1998	Statin vs non active	Secondary	Private	Life Years Gained	$ USA (1997)	16904	16904
15	Huse	1998	Statin vs non active	Secondary	Private	Life Years Gained	$ USA (1997)	16962	16962

15	Huse	1998	Statin vs non active	Secondary	Private	Life Years Gained	$ USA (1997)	17414	17414
15	Huse	1998	Statin vs non active	Secondary	Private	Life Years Gained	$ USA (1997)	17475	17475
15	Huse	1998	Statin vs non active	Secondary	Private	Life Years Gained	$ USA (1997)	17575	17575
15	Huse	1998	Statin vs non active	Secondary	Private	Life Years Gained	$ USA (1997)	17609	17609
15	Huse	1998	Statin vs statin	Primary	Private	Life Years Gained	$ USA (1997)	17664	17664
15	Huse	1998	Statin vs non active	Secondary	Private	Life Years Gained	$ USA (1997)	17689	17689
15	Huse	1998	Statin vs non active	Secondary	Private	Life Years Gained	$ USA (1997)	17693	17693
15	Huse	1998	Statin vs non active	Secondary	Private	Life Years Gained	$ USA (1997)	17918	17918
15	Huse	1998	Statin vs non active	Secondary	Private	Life Years Gained	$ USA (1997)	18061	18061
15	Huse	1998	Statin vs non active	Primary	Private	Life Years Gained	$ USA (1997)	18064	18064
15	Huse	1998	Statin vs non active	Secondary	Private	Life Years Gained	$ USA (1997)	18509	18509
15	Huse	1998	Statin vs non active	Secondary	Private	Life Years Gained	$ USA (1997)	18524	18524
15	Huse	1998	Statin vs statin	Primary	Private	Life Years Gained	$ USA (1997)	18609	18609
15	Huse	1998	Statin vs non active	Primary	Private	Life Years Gained	$ USA (1997)	18862	18862
15	Huse	1998	Statin vs non active	Secondary	Private	Life Years Gained	$ USA (1997)	19480	19480
15	Huse	1998	Statin vs non active	Secondary	Private	Life Years Gained	$ USA (1997)	19557	19557
15	Huse	1998	Statin vs non active	Primary	Private	Life Years Gained	$ USA (1997)	19724	19724
15	Huse	1998	Statin vs statin	Primary	Private	Life Years Gained	$ USA (1997)	19730	19730
15	Huse	1998	Statin vs statin	Secondary	Private	Life Years Gained	$ USA (1997)	19784	19784
15	Huse	1998	Statin vs non active	Secondary	Private	Life Years Gained	$ USA (1997)	20067	20067
15	Huse	1998	Statin vs non active	Secondary	Private	Life Years Gained	$ USA (1997)	20075	20075
15	Huse	1998	Statin vs non active	Secondary	Private	Life Years Gained	$ USA (1997)	20099	20099
15	Huse	1998	Statin vs non active	Secondary	Private	Life Years Gained	$ USA (1997)	20109	20109
15	Huse	1998	Statin vs non active	Secondary	Private	Life Years Gained	$ USA (1997)	20150	20150
15	Huse	1998	Statin vs non active	Primary	Private	Life Years Gained	$ USA (1997)	20215	20215
15	Huse	1998	Statin vs non active	Secondary	Private	Life Years Gained	$ USA (1997)	20225	20225
15	Huse	1998	Statin vs non active	Secondary	Private	Life Years Gained	$ USA (1997)	20661	20661
15	Huse	1998	Statin vs non active	Secondary	Private	Life Years Gained	$ USA (1997)	20675	20675
15	Huse	1998	Statin vs non active	Secondary	Private	Life Years Gained	$ USA (1997)	20687	20687
15	Huse	1998	Statin vs non active	Primary	Private	Life Years Gained	$ USA (1997)	20975	20975
15	Huse	1998	Statin vs non active	Secondary	Private	Life Years Gained	$ USA (1997)	21364	21364
15	Huse	1998	Statin vs non active	Secondary	Private	Life Years Gained	$ USA (1997)	21474	21474
15	Huse	1998	Statin vs non active	Secondary	Private	Life Years Gained	$ USA (1997)	21512	21512
15	Huse	1998	Statin vs statin	Secondary	Private	Life Years Gained	$ USA (1997)	21918	21918
15	Huse	1998	Statin vs non active	Secondary	Private	Life Years Gained	$ USA (1997)	22084	22084
15	Huse	1998	Statin vs non active	Primary	Private	Life Years Gained	$ USA (1997)	22333	22333
15	Huse	1998	Statin vs statin	Primary	Private	Life Years Gained	$ USA (1997)	22356	22356
15	Huse	1998	Statin vs non active	Secondary	Private	Life Years Gained	$ USA (1997)	22400	22400
15	Huse	1998	Statin vs non active	Secondary	Private	Life Years Gained	$ USA (1997)	22490	22490
15	Huse	1998	Statin vs statin	Secondary	Private	Life Years Gained	$ USA (1997)	22512	22512
15	Huse	1998	Statin vs non active	Secondary	Private	Life Years Gained	$ USA (1997)	22524	22524
15	Huse	1998	Statin vs non active	Secondary	Private	Life Years Gained	$ USA (1997)	22544	22544
15	Huse	1998	Statin vs non active	Secondary	Private	Life Years Gained	$ USA (1997)	22681	22681

15	Huse	1998	Statin vs non active	Secondary	Private	Life Years Gained	$ USA (1997)	22695	22695
15	Huse	1998	Statin vs non active	Primary	Private	Life Years Gained	$ USA (1997)	23139	23139
15	Huse	1998	Statin vs non active	Primary	Private	Life Years Gained	$ USA (1997)	23549	23549
15	Huse	1998	Statin vs non active	Secondary	Private	Life Years Gained	$ USA (1997)	23558	23558
15	Huse	1998	Statin vs non active	Secondary	Private	Life Years Gained	$ USA (1997)	23632	23632
15	Huse	1998	Statin vs non active	Secondary	Private	Life Years Gained	$ USA (1997)	23934	23934
15	Huse	1998	Statin vs non active	Secondary	Private	Life Years Gained	$ USA (1997)	24200	24200
15	Huse	1998	Statin vs non active	Secondary	Private	Life Years Gained	$ USA (1997)	24797	24797
15	Huse	1998	Statin vs non active	Secondary	Private	Life Years Gained	$ USA (1997)	25140	25140
15	Huse	1998	Statin vs non active	Secondary	Private	Life Years Gained	$ USA (1997)	25206	25206
15	Huse	1998	Statin vs statin	Primary	Private	Life Years Gained	$ USA (1997)	25249	25249
15	Huse	1998	Statin vs non active	Primary	Private	Life Years Gained	$ USA (1997)	25653	25653
15	Huse	1998	Statin vs non active	Primary	Private	Life Years Gained	$ USA (1997)	25750	25750
15	Huse	1998	Statin vs non active	Secondary	Private	Life Years Gained	$ USA (1997)	25774	25774
15	Huse	1998	Statin vs non active	Primary	Private	Life Years Gained	$ USA (1997)	25931	25931
15	Huse	1998	Statin vs non active	Secondary	Private	Life Years Gained	$ USA (1997)	26272	26272
15	Huse	1998	Statin vs non active	Secondary	Private	Life Years Gained	$ USA (1997)	26747	26747
15	Huse	1998	Statin vs non active	Secondary	Private	Life Years Gained	$ USA (1997)	27039	27039
15	Huse	1998	Statin vs non active	Primary	Private	Life Years Gained	$ USA (1997)	27200	27200
15	Huse	1998	Statin vs non active	Secondary	Private	Life Years Gained	$ USA (1997)	27229	27229
15	Huse	1998	Statin vs non active	Secondary	Private	Life Years Gained	$ USA (1997)	27389	27389
15	Huse	1998	Statin vs non active	Secondary	Private	Life Years Gained	$ USA (1997)	27810	27810
15	Huse	1998	Statin vs statin	Primary	Private	Life Years Gained	$ USA (1997)	27819	27819
15	Huse	1998	Statin vs statin	Primary	Private	Life Years Gained	$ USA (1997)	28037	28037
15	Huse	1998	Statin vs non active	Primary	Private	Life Years Gained	$ USA (1997)	28336	28336
15	Huse	1998	Statin vs non active	Primary	Private	Life Years Gained	$ USA (1997)	28940	28940
15	Huse	1998	Statin vs non active	Primary	Private	Life Years Gained	$ USA (1997)	29015	29015
15	Huse	1998	Statin vs non active	Secondary	Private	Life Years Gained	$ USA (1997)	30179	30179
15	Huse	1998	Statin vs non active	Primary	Private	Life Years Gained	$ USA (1997)	30493	30493
15	Huse	1998	Statin vs non active	Secondary	Private	Life Years Gained	$ USA (1997)	30505	30505
15	Huse	1998	Statin vs statin	Primary	Private	Life Years Gained	$ USA (1997)	30715	30715
15	Huse	1998	Statin vs non active	Secondary	Private	Life Years Gained	$ USA (1997)	30776	30776
15	Huse	1998	Statin vs non active	Secondary	Private	Life Years Gained	$ USA (1997)	30822	30822
15	Huse	1998	Statin vs non active	Primary	Private	Life Years Gained	$ USA (1997)	31170	31170
15	Huse	1998	Statin vs non active	Primary	Private	Life Years Gained	$ USA (1997)	31544	31544
15	Huse	1998	Statin vs non active	Secondary	Private	Life Years Gained	$ USA (1997)	31665	31665
15	Huse	1998	Statin vs non active	Secondary	Private	Life Years Gained	$ USA (1997)	31819	31819
15	Huse	1998	Statin vs non active	Secondary	Private	Life Years Gained	$ USA (1997)	32123	32123
15	Huse	1998	Statin vs non active	Secondary	Private	Life Years Gained	$ USA (1997)	32292	32292
15	Huse	1998	Statin vs non active	Primary	Private	Life Years Gained	$ USA (1997)	32609	32609
15	Huse	1998	Statin vs non active	Secondary	Private	Life Years Gained	$ USA (1997)	32662	32662
15	Huse	1998	Statin vs non active	Secondary	Private	Life Years Gained	$ USA (1997)	32735	32735
15	Huse	1998	Statin vs non active	Primary	Private	Life Years Gained	$ USA (1997)	32914	32914

15	Huse	1998	Statin vs non active	Primary	Private	Life Years Gained	$ USA (1997)	33142	33142
15	Huse	1998	Statin vs non active	Primary	Private	Life Years Gained	$ USA (1997)	33207	33207
15	Huse	1998	Statin vs non active	Primary	Private	Life Years Gained	$ USA (1997)	33910	33910
15	Huse	1998	Statin vs non active	Secondary	Private	Life Years Gained	$ USA (1997)	34859	34859
15	Huse	1998	Statin vs non active	Primary	Private	Life Years Gained	$ USA (1997)	34900	34900
15	Huse	1998	Statin vs non active	Secondary	Private	Life Years Gained	$ USA (1997)	35543	35543
15	Huse	1998	Statin vs non active	Secondary	Private	Life Years Gained	$ USA (1997)	35913	35913
15	Huse	1998	Statin vs non active	Primary	Private	Life Years Gained	$ USA (1997)	36335	36335
15	Huse	1998	Statin vs non active	Primary	Private	Life Years Gained	$ USA (1997)	37495	37495
15	Huse	1998	Statin vs non active	Primary	Private	Life Years Gained	$ USA (1997)	37722	37722
15	Huse	1998	Statin vs non active	Primary	Private	Life Years Gained	$ USA (1997)	38432	38432
15	Huse	1998	Statin vs non active	Secondary	Private	Life Years Gained	$ USA (1997)	38540	38540
15	Huse	1998	Statin vs non active	Secondary	Private	Life Years Gained	$ USA (1997)	38589	38589
15	Huse	1998	Statin vs non active	Primary	Private	Life Years Gained	$ USA (1997)	39247	39247
15	Huse	1998	Statin vs non active	Primary	Private	Life Years Gained	$ USA (1997)	39583	39583
15	Huse	1998	Statin vs non active	Secondary	Private	Life Years Gained	$ USA (1997)	40730	40730
15	Huse	1998	Statin vs non active	Secondary	Private	Life Years Gained	$ USA (1997)	40861	40861
15	Huse	1998	Statin vs non active	Primary	Private	Life Years Gained	$ USA (1997)	40978	40978
15	Huse	1998	Statin vs non active	Secondary	Private	Life Years Gained	$ USA (1997)	41577	41577
15	Huse	1998	Statin vs non active	Primary	Private	Life Years Gained	$ USA (1997)	41736	41736
15	Huse	1998	Statin vs non active	Primary	Private	Life Years Gained	$ USA (1997)	41890	41890
15	Huse	1998	Statin vs non active	Secondary	Private	Life Years Gained	$ USA (1997)	42170	42170
15	Huse	1998	Statin vs non active	Primary	Private	Life Years Gained	$ USA (1997)	42624	42624
15	Huse	1998	Statin vs non active	Primary	Private	Life Years Gained	$ USA (1997)	42738	42738
15	Huse	1998	Statin vs non active	Secondary	Private	Life Years Gained	$ USA (1997)	43117	43117
15	Huse	1998	Statin vs non active	Primary	Private	Life Years Gained	$ USA (1997)	43823	43823
15	Huse	1998	Statin vs non active	Primary	Private	Life Years Gained	$ USA (1997)	43862	43862
15	Huse	1998	Statin vs non active	Primary	Private	Life Years Gained	$ USA (1997)	44036	44036
15	Huse	1998	Statin vs non active	Primary	Private	Life Years Gained	$ USA (1997)	44036	44036
15	Huse	1998	Statin vs non active	Secondary	Private	Life Years Gained	$ USA (1997)	45737	45737
15	Huse	1998	Statin vs non active	Primary	Private	Life Years Gained	$ USA (1997)	46573	46573
15	Huse	1998	Statin vs non active	Primary	Private	Life Years Gained	$ USA (1997)	46693	46693
15	Huse	1998	Statin vs non active	Secondary	Private	Life Years Gained	$ USA (1997)	46769	46769
15	Huse	1998	Statin vs non active	Primary	Private	Life Years Gained	$ USA (1997)	47077	47077
15	Huse	1998	Statin vs non active	Primary	Private	Life Years Gained	$ USA (1997)	47201	47201
15	Huse	1998	Statin vs non active	Secondary	Private	Life Years Gained	$ USA (1997)	47373	47373
15	Huse	1998	Statin vs non active	Primary	Private	Life Years Gained	$ USA (1997)	47537	47537
15	Huse	1998	Statin vs non active	Primary	Private	Life Years Gained	$ USA (1997)	47588	47588
15	Huse	1998	Statin vs non active	Secondary	Private	Life Years Gained	$ USA (1997)	47677	47677
15	Huse	1998	Statin vs non active	Primary	Private	Life Years Gained	$ USA (1997)	50266	50266
15	Huse	1998	Statin vs non active	Secondary	Private	Life Years Gained	$ USA (1997)	50905	50905
15	Huse	1998	Statin vs non active	Secondary	Private	Life Years Gained	$ USA (1997)	51756	51756
15	Huse	1998	Statin vs non active	Primary	Private	Life Years Gained	$ USA (1997)	52297	52297

15	Huse	1998	Statin vs non active	Primary	Private	Life Years Gained	$ USA (1997)	52623	52623
15	Huse	1998	Statin vs non active	Primary	Private	Life Years Gained	$ USA (1997)	52649	52649
15	Huse	1998	Statin vs non active	Primary	Private	Life Years Gained	$ USA (1997)	52813	52813
15	Huse	1998	Statin vs non active	Primary	Private	Life Years Gained	$ USA (1997)	55873	55873
15	Huse	1998	Statin vs non active	Primary	Private	Life Years Gained	$ USA (1997)	56492	56492
15	Huse	1998	Statin vs non active	Primary	Private	Life Years Gained	$ USA (1997)	56690	56690
15	Huse	1998	Statin vs non active	Secondary	Private	Life Years Gained	$ USA (1997)	56871	56871
15	Huse	1998	Statin vs non active	Primary	Private	Life Years Gained	$ USA (1997)	56912	56912
15	Huse	1998	Statin vs non active	Primary	Private	Life Years Gained	$ USA (1997)	58356	58356
15	Huse	1998	Statin vs non active	Primary	Private	Life Years Gained	$ USA (1997)	59036	59036
15	Huse	1998	Statin vs non active	Primary	Private	Life Years Gained	$ USA (1997)	60004	60004
15	Huse	1998	Statin vs non active	Secondary	Private	Life Years Gained	$ USA (1997)	62062	62062
15	Huse	1998	Statin vs non active	Primary	Private	Life Years Gained	$ USA (1997)	62967	62967
15	Huse	1998	Statin vs non active	Primary	Private	Life Years Gained	$ USA (1997)	63076	63076
15	Huse	1998	Statin vs non active	Secondary	Private	Life Years Gained	$ USA (1997)	63614	63614
15	Huse	1998	Statin vs non active	Primary	Private	Life Years Gained	$ USA (1997)	65408	65408
15	Huse	1998	Statin vs non active	Primary	Private	Life Years Gained	$ USA (1997)	67307	67307
15	Huse	1998	Statin vs non active	Primary	Private	Life Years Gained	$ USA (1997)	70066	70066
15	Huse	1998	Statin vs non active	Primary	Private	Life Years Gained	$ USA (1997)	77389	77389
15	Huse	1998	Statin vs non active	Primary	Private	Life Years Gained	$ USA (1997)	77908	77908
15	Huse	1998	Statin vs non active	Primary	Private	Life Years Gained	$ USA (1997)	82993	82993
15	Huse	1998	Statin vs statin	Primary	Private	Life Years Gained	$ USA (1997)	106985	106985
15	Huse	1998	Statin vs statin	Primary	Private	Life Years Gained	$ USA (1997)	127469	127469
15	Huse	1998	Statin vs statin	Primary	Private	Life Years Gained	$ USA (1997)	139882	139882
15	Huse	1998	Statin vs non active	Primary	Private	Life Years Gained	$ USA (1997)	160918	160918
15	Huse	1998	Statin vs statin	Primary	Private	Life Years Gained	$ USA (1997)	162917	162917
15	Huse	1998	Statin vs statin	Primary	Private	Life Years Gained	$ USA (1997)	185608	185608
15	Huse	1998	Statin vs non active	Primary	Private	Life Years Gained	$ USA (1997)	189631	189631
15	Huse	1998	Statin vs non active	Primary	Private	Life Years Gained	$ USA (1997)	190490	190490
15	Huse	1998	Statin vs non active	Primary	Private	Life Years Gained	$ USA (1997)	201558	201558
15	Huse	1998	Statin vs non active	Primary	Private	Life Years Gained	$ USA (1997)	208349	208349
15	Huse	1998	Statin vs non active	Primary	Private	Life Years Gained	$ USA (1997)	222107	222107
15	Huse	1998	Statin vs non active	Primary	Private	Life Years Gained	$ USA (1997)	225201	225201
15	Huse	1998	Statin vs non active	Primary	Private	Life Years Gained	$ USA (1997)	235199	235199
15	Huse	1998	Statin vs non active	Primary	Private	Life Years Gained	$ USA (1997)	245472	245472
15	Huse	1998	Statin vs non active	Primary	Private	Life Years Gained	$ USA (1997)	245631	245631
15	Huse	1998	Statin vs non active	Primary	Private	Life Years Gained	$ USA (1997)	258942	258942
15	Huse	1998	Statin vs non active	Primary	Private	Life Years Gained	$ USA (1997)	259754	259754
15	Huse	1998	Statin vs non active	Primary	Private	Life Years Gained	$ USA (1997)	274229	274229
15	Huse	1998	Statin vs non active	Primary	Private	Life Years Gained	$ USA (1997)	277332	277332
15	Huse	1998	Statin vs non active	Primary	Private	Life Years Gained	$ USA (1997)	289028	289028
15	Huse	1998	Statin vs non active	Primary	Private	Life Years Gained	$ USA (1997)	289711	289711
15	Huse	1998	Statin vs non active	Primary	Private	Life Years Gained	$ USA (1997)	308411	308411

15	Huse	1998	Statin vs non active	Primary	Private	Life Years Gained	$ USA (1997)	318991	318991
15	Huse	1998	Statin vs non active	Primary	Private	Life Years Gained	$ USA (1997)	325976	325976
15	Huse	1998	Statin vs non active	Primary	Private	Life Years Gained	$ USA (1997)	341803	341803
15	Huse	1998	Statin vs non active	Primary	Private	Life Years Gained	$ USA (1997)	345875	345875
15	Huse	1998	Statin vs non active	Primary	Private	Life Years Gained	$ USA (1997)	352403	352403
15	Huse	1998	Statin vs non active	Primary	Private	Life Years Gained	$ USA (1997)	382790	382790
15	Huse	1998	Statin vs non active	Primary	Private	Life Years Gained	$ USA (1997)	417060	417060
15	Huse	1998	Statin vs non active	Primary	Private	Life Years Gained	$ USA (1997)	468115	468115
16	Perreault	1998	Statin vs non active	Primary	Public or non-profit	Life Years Gained	$ Canadian (1996)	11040	11504
16	Perreault	1998	Statin vs statin	Primary	Public or non-profit	Life Years Gained	$ Canadian (1996)	14469	15077
16	Perreault	1998	Statin vs non active	Primary	Public or non-profit	Life Years Gained	$ Canadian (1996)	18666	19450
16	Perreault	1998	Statin vs non active	Primary	Public or non-profit	Life Years Gained	$ Canadian (1996)	21461	22363
16	Perreault	1998	Statin vs statin	Primary	Public or non-profit	Life Years Gained	$ Canadian (1996)	24369	25393
16	Perreault	1998	Statin vs non active	Primary	Public or non-profit	Life Years Gained	$ Canadian (1996)	24391	25416
16	Perreault	1998	Statin vs statin	Primary	Public or non-profit	Life Years Gained	$ Canadian (1996)	24648	25684
16	Perreault	1998	Statin vs statin	Primary	Public or non-profit	Life Years Gained	$ Canadian (1996)	25065	26118
16	Perreault	1998	Statin vs non active	Primary	Public or non-profit	Life Years Gained	$ Canadian (1996)	27653	28815
16	Perreault	1998	Statin vs statin	Primary	Public or non-profit	Life Years Gained	$ Canadian (1996)	27872	29043
16	Perreault	1998	Statin vs non active	Primary	Public or non-profit	Life Years Gained	$ Canadian (1996)	29105	30328
16	Perreault	1998	Statin vs non active	Primary	Public or non-profit	Life Years Gained	$ Canadian (1996)	29215	30443
16	Perreault	1998	Statin vs statin	Primary	Public or non-profit	Life Years Gained	$ Canadian (1996)	30067	31330
16	Perreault	1998	Statin vs statin	Primary	Public or non-profit	Life Years Gained	$ Canadian (1996)	35499	36991
16	Perreault	1998	Statin vs non active	Primary	Public or non-profit	Life Years Gained	$ Canadian (1996)	35562	37056
16	Perreault	1998	Statin vs statin	Primary	Public or non-profit	Life Years Gained	$ Canadian (1996)	40059	41742
16	Perreault	1998	Statin vs non active	Primary	Public or non-profit	Life Years Gained	$ Canadian (1996)	40759	42472
16	Perreault	1998	Statin vs statin	Primary	Public or non-profit	Life Years Gained	$ Canadian (1996)	41433	43174
16	Perreault	1998	Statin vs statin	Primary	Public or non-profit	Life Years Gained	$ Canadian (1996)	42437	44220
16	Perreault	1998	Statin vs statin	Primary	Public or non-profit	Life Years Gained	$ Canadian (1996)	42593	44383
16	Perreault	1998	Statin vs non active	Primary	Public or non-profit	Life Years Gained	$ Canadian (1996)	44807	46690
16	Perreault	1998	Statin vs statin	Primary	Public or non-profit	Life Years Gained	$ Canadian (1996)	45987	47919
16	Perreault	1998	Statin vs statin	Primary	Public or non-profit	Life Years Gained	$ Canadian (1996)	48321	50351
16	Perreault	1998	Statin vs statin	Primary	Public or non-profit	Life Years Gained	$ Canadian (1996)	48914	50969
16	Perreault	1998	Statin vs non active	Primary	Public or non-profit	Life Years Gained	$ Canadian (1996)	51444	53606
16	Perreault	1998	Statin vs statin	Primary	Public or non-profit	Life Years Gained	$ Canadian (1996)	51592	53760
16	Perreault	1998	Statin vs non active	Primary	Public or non-profit	Life Years Gained	$ Canadian (1996)	52463	54667
16	Perreault	1998	Statin vs statin	Primary	Public or non-profit	Life Years Gained	$ Canadian (1996)	56381	58750
16	Perreault	1998	Statin vs statin	Primary	Public or non-profit	Life Years Gained	$ Canadian (1996)	61357	63935
16	Perreault	1998	Statin vs statin	Primary	Public or non-profit	Life Years Gained	$ Canadian (1996)	68750	71639
16	Perreault	1998	Statin vs statin	Primary	Public or non-profit	Life Years Gained	$ Canadian (1996)	71258	74252
16	Perreault	1998	Statin vs statin	Primary	Public or non-profit	Life Years Gained	$ Canadian (1996)	73938	77045
16	Perreault	1998	Statin vs statin	Primary	Public or non-profit	Life Years Gained	$ Canadian (1996)	74371	77496
16	Perreault	1998	Statin vs statin	Primary	Public or non-profit	Life Years Gained	$ Canadian (1996)	77456	80711
16	Perreault	1998	Statin vs statin	Primary	Public or non-profit	Life Years Gained	$ Canadian (1996)	100726	104959

16	Perreault	1998	Statin vs statin	Primary	Public or non-profit	Life Years Gained	$ Canadian (1996)	101567	105835
17	Lindholm	1999	Statin vs non active	Secondary	None	Life Years Gained	ECU (1996)	47200	67090
17	Lindholm	1999	Statin vs non active	Secondary	None	Life Years Gained	ECU (1996)	53400	75903
17	Lindholm	1999	Statin vs non active	Secondary	None	Life Years Gained	ECU (1996)	60800	86421
17	Lindholm	1999	Statin vs non active	Secondary	None	Life Years Gained	ECU (1996)	64500	91680
17	Lindholm	1999	Statin vs non active	Secondary	None	Life Years Gained	ECU (1996)	72200	102625
17	Lindholm	1999	Statin vs non active	Secondary	None	Life Years Gained	ECU (1996)	73000	103762
17	Lindholm	1999	Statin vs non active	Secondary	None	Life Years Gained	ECU (1996)	73700	104757
17	Lindholm	1999	Statin vs non active	Secondary	None	Life Years Gained	ECU (1996)	84400	119966
17	Lindholm	1999	Statin vs non active	Secondary	None	Life Years Gained	ECU (1996)	85400	121388
17	Lindholm	1999	Statin vs non active	Secondary	None	Life Years Gained	ECU (1996)	91800	130485
17	Lindholm	1999	Statin vs non active	Secondary	None	Life Years Gained	ECU (1996)	97800	139013
17	Lindholm	1999	Statin vs non active	Secondary	None	Life Years Gained	ECU (1996)	103000	146404
17	Lindholm	1999	Statin vs non active	Secondary	None	Life Years Gained	ECU (1996)	104400	148394
17	Lindholm	1999	Statin vs non active	Secondary	None	Life Years Gained	ECU (1996)	118600	168578
17	Lindholm	1999	Statin vs non active	Secondary	None	Life Years Gained	ECU (1996)	122200	173695
17	Lindholm	1999	Statin vs non active	Secondary	None	Life Years Gained	ECU (1996)	124100	176396
17	Lindholm	1999	Statin vs non active	Secondary	None	Life Years Gained	ECU (1996)	141100	200560
17	Lindholm	1999	Statin vs non active	Secondary	None	Life Years Gained	ECU (1996)	148500	211078
17	Lindholm	1999	Statin vs non active	Secondary	None	Life Years Gained	ECU (1996)	153900	218753
17	Lindholm	1999	Statin vs non active	Secondary	None	Life Years Gained	ECU (1996)	172400	245049
17	Lindholm	1999	Statin vs non active	Secondary	None	Life Years Gained	ECU (1996)	175800	249882
17	Lindholm	1999	Statin vs non active	Secondary	None	Life Years Gained	ECU (1996)	208900	296930
17	Lindholm	1999	Statin vs non active	Secondary	None	Life Years Gained	ECU (1996)	213800	303895
17	Lindholm	1999	Statin vs non active	Secondary	None	Life Years Gained	ECU (1996)	262700	373402
17	Lindholm	1999	Statin vs non active	Secondary	None	Life Years Gained	ECU (1996)	262700	373402
17	Lindholm	1999	Statin vs non active	Secondary	None	Life Years Gained	ECU (1996)	295700	420308
17	Lindholm	1999	Statin vs non active	Secondary	None	Life Years Gained	ECU (1996)	295700	420308
17	Lindholm	1999	Statin vs non active	Secondary	None	Life Years Gained	ECU (1996)	377200	536152
17	Lindholm	1999	Statin vs non active	Secondary	None	Life Years Gained	ECU (1996)	467100	663936
17	Lindholm	1999	Statin vs non active	Secondary	None	Life Years Gained	ECU (1996)	467100	663936
17	Lindholm	1999	Statin vs non active	Secondary	None	Life Years Gained	ECU (1996)	515600	732874
17	Lindholm	1999	Statin vs non active	Secondary	None	Life Years Gained	ECU (1996)	803100	1141526
18	Grover	1999	Statin vs non active	Secondary	None	Life Years Gained	$ USA (1996)	4419	4419
18	Grover	1999	Statin vs non active	Secondary	None	Life Years Gained	$ USA (1996)	4430	4430
18	Grover	1999	Statin vs non active	Secondary	None	Life Years Gained	$ USA (1996)	4471	4471
18	Grover	1999	Statin vs non active	Secondary	None	Life Years Gained	$ USA (1996)	4487	4487
18	Grover	1999	Statin vs non active	Secondary	None	Life Years Gained	$ USA (1996)	4573	4573
18	Grover	1999	Statin vs non active	Secondary	None	Life Years Gained	$ USA (1996)	4675	4675
18	Grover	1999	Statin vs non active	Secondary	None	Life Years Gained	$ USA (1996)	4927	4927
18	Grover	1999	Statin vs non active	Secondary	None	Life Years Gained	$ USA (1996)	4947	4947
18	Grover	1999	Statin vs non active	Secondary	None	Life Years Gained	$ USA (1996)	4996	4996
18	Grover	1999	Statin vs non active	Secondary	None	Life Years Gained	$ USA (1996)	5016	5016

18	Grover	1999	Statin vs non active	Secondary	None	Life Years Gained	$ USA (1996)	5138	5138
18	Grover	1999	Statin vs non active	Secondary	None	Life Years Gained	$ USA (1996)	5182	5182
18	Grover	1999	Statin vs non active	Secondary	None	Life Years Gained	$ USA (1996)	5241	5241
18	Grover	1999	Statin vs non active	Secondary	None	Life Years Gained	$ USA (1996)	5420	5420
18	Grover	1999	Statin vs non active	Secondary	None	Life Years Gained	$ USA (1996)	5424	5424
18	Grover	1999	Statin vs non active	Secondary	None	Life Years Gained	$ USA (1996)	5629	5629
18	Grover	1999	Statin vs non active	Secondary	None	Life Years Gained	$ USA (1996)	5841	5841
18	Grover	1999	Statin vs non active	Secondary	None	Life Years Gained	$ USA (1996)	6050	6050
18	Grover	1999	Statin vs non active	Secondary	None	Life Years Gained	$ USA (1996)	6573	6573
18	Grover	1999	Statin vs non active	Secondary	None	Life Years Gained	$ USA (1996)	6875	6875
18	Grover	1999	Statin vs non active	Secondary	None	Life Years Gained	$ USA (1996)	7072	7072
18	Grover	1999	Statin vs non active	Secondary	None	Life Years Gained	$ USA (1996)	7447	7447
18	Grover	1999	Statin vs non active	Secondary	None	Life Years Gained	$ USA (1996)	7738	7738
18	Grover	1999	Statin vs non active	Secondary	None	Life Years Gained	$ USA (1996)	7775	7775
18	Grover	1999	Statin vs non active	Secondary	None	Life Years Gained	$ USA (1996)	7797	7797
18	Grover	1999	Statin vs non active	Secondary	None	Life Years Gained	$ USA (1996)	7947	7947
18	Grover	1999	Statin vs non active	Secondary	None	Life Years Gained	$ USA (1996)	7991	7991
18	Grover	1999	Statin vs non active	Secondary	None	Life Years Gained	$ USA (1996)	8042	8042
18	Grover	1999	Statin vs non active	Secondary	None	Life Years Gained	$ USA (1996)	8389	8389
18	Grover	1999	Statin vs non active	Secondary	None	Life Years Gained	$ USA (1996)	8389	8389
18	Grover	1999	Statin vs non active	Secondary	None	Life Years Gained	$ USA (1996)	8532	8532
18	Grover	1999	Statin vs non active	Secondary	None	Life Years Gained	$ USA (1996)	9010	9010
18	Grover	1999	Statin vs non active	Secondary	None	Life Years Gained	$ USA (1996)	9548	9548
18	Grover	1999	Statin vs non active	Secondary	None	Life Years Gained	$ USA (1996)	9926	9926
18	Grover	1999	Statin vs non active	Secondary	None	Life Years Gained	$ USA (1996)	10072	10072
18	Grover	1999	Statin vs non active	Secondary	None	Life Years Gained	$ USA (1996)	11412	11412
18	Grover	1999	Statin vs non active	Secondary	None	Life Years Gained	$ USA (1996)	11665	11665
18	Grover	1999	Statin vs non active	Secondary	None	Life Years Gained	$ USA (1996)	11761	11761
18	Grover	1999	Statin vs non active	Secondary	None	Life Years Gained	$ USA (1996)	13090	13090
18	Grover	1999	Statin vs non active	Secondary	None	Life Years Gained	$ USA (1996)	13404	13404
18	Grover	1999	Statin vs non active	Secondary	None	Life Years Gained	$ USA (1996)	13477	13477
18	Grover	1999	Statin vs non active	Secondary	None	Life Years Gained	$ USA (1996)	13582	13582
18	Grover	1999	Statin vs non active	Secondary	None	Life Years Gained	$ USA (1996)	13747	13747
18	Grover	1999	Statin vs non active	Secondary	None	Life Years Gained	$ USA (1996)	15984	15984
18	Grover	1999	Statin vs non active	Secondary	None	Life Years Gained	$ USA (1996)	17745	17745
18	Grover	1999	Statin vs non active	Secondary	None	Life Years Gained	$ USA (1996)	18397	18397
18	Grover	1999	Statin vs non active	Secondary	None	Life Years Gained	$ USA (1996)	20987	20987
18	Grover	1999	Statin vs non active	Secondary	None	Life Years Gained	$ USA (1996)	21719	21719
19	Morris	1999	Statin vs non active	Primary	Private	Life Years Gained	$ Canadian (1998)	36645	38185
19	Morris	1999	Statin vs statin	Primary	Private	Life Years Gained	$ Canadian (1998)	38088	39688
19	Morris	1999	Statin vs statin	Primary	Private	Life Years Gained	$ Canadian (1998)	40407	42105
19	Morris	1999	Statin vs statin	Primary	Private	Life Years Gained	$ Canadian (1998)	41648	43398
19	Morris	1999	Statin vs statin	Primary	Private	Life Years Gained	$ Canadian (1998)	45506	47418

19	Morris	1999	Statin vs statin	Primary	Private	Life Years Gained	$ Canadian (1998)	55959	58310
19	Morris	1999	Statin vs statin	Primary	Private	Life Years Gained	$ Canadian (1998)	56567	58944
19	Morris	1999	Statin vs statin	Primary	Private	Life Years Gained	$ Canadian (1998)	57571	59990
19	Morris	1999	Statin vs statin	Primary	Private	Life Years Gained	$ Canadian (1998)	59583	62087
19	Morris	1999	Statin vs statin	Primary	Private	Life Years Gained	$ Canadian (1998)	59590	62094
19	Morris	1999	Statin vs statin	Primary	Private	Life Years Gained	$ Canadian (1998)	61811	64408
19	Morris	1999	Statin vs statin	Primary	Private	Life Years Gained	$ Canadian (1998)	63382	66045
19	Morris	1999	Statin vs statin	Primary	Private	Life Years Gained	$ Canadian (1998)	73440	76526
19	Morris	1999	Statin vs statin	Primary	Private	Life Years Gained	$ Canadian (1998)	76225	79428
19	Morris	1999	Statin vs statin	Primary	Private	Life Years Gained	$ Canadian (1998)	89437	93195
19	Morris	1999	Statin vs statin	Primary	Private	Life Years Gained	$ Canadian (1998)	134600	140256
19	Morris	1999	Statin vs statin	Primary	Private	Life Years Gained	$ Canadian (1998)	335669	349774
20	Ebrahim	1999	Statin vs statin	Secondary	Public or non-profit	Life Years Gained	£ UK (1997)	2188	3531
20	Ebrahim	1999	Statin vs statin	Primary	Public or non-profit	Life Years Gained	£ UK (1997)	4889	7889
20	Ebrahim	1999	Statin vs statin	Secondary	Public or non-profit	Life Years Gained	£ UK (1997)	6096	9837
20	Ebrahim	1999	Statin vs statin	Secondary	Public or non-profit	Life Years Gained	£ UK (1997)	7721	12459
20	Ebrahim	1999	Statin vs statin	Primary	Public or non-profit	Life Years Gained	£ UK (1997)	10452	16866
20	Ebrahim	1999	Statin vs statin	Primary	Public or non-profit	Life Years Gained	£ UK (1997)	12767	20601
21	Elliot	1999	Statin vs statin	Secondary	Private	Life Years Gained	$ USA (1999)	5421	5421
21	Elliot	1999	Statin vs statin	Secondary	Private	Life Years Gained	$ USA (1999)	5790	5790
21	Elliot	1999	Statin vs statin	Secondary	Private	Life Years Gained	$ USA (1999)	6158	6158
21	Elliot	1999	Statin vs statin	Secondary	Private	Life Years Gained	$ USA (1999)	8575	8575
21	Elliot	1999	Statin vs statin	Secondary	Private	Life Years Gained	$ USA (1999)	9232	9232
21	Elliot	1999	Statin vs statin	Secondary	Private	Life Years Gained	$ USA (1999)	15073	15073
22	Pickin	1999	Statin vs non active	Secondary	Public or non-profit	Life Years Gained	£ UK (1997)	5100	8230
22	Pickin	1999	Statin vs non active	Primary	Public or non-profit	Life Years Gained	£ UK (1997)	8200	13232
22	Pickin	1999	Statin vs non active	Primary	Public or non-profit	Life Years Gained	£ UK (1997)	10700	17266
22	Pickin	1999	Statin vs non active	Primary	Public or non-profit	Life Years Gained	£ UK (1997)	12500	20171
23	Jönsson	1999	Statin vs non active	Secondary	Private	Life Years Gained	Euro (1995-1997)	0	0
23	Jönsson	1999	Statin vs non active	Secondary	Private	Life Years Gained	Euro (1995-1997)	0	0
23	Jönsson	1999	Statin vs non active	Secondary	Private	Life Years Gained	Euro (1995-1997)	0	0
23	Jönsson	1999	Statin vs non active	Secondary	Private	Life Years Gained	Euro (1995-1997)	0	0
23	Jönsson	1999	Statin vs non active	Secondary	Private	Life Years Gained	Euro (1995-1997)	89	127
23	Jönsson	1999	Statin vs non active	Secondary	Private	Life Years Gained	Euro (1995-1997)	881	1252
23	Jönsson	1999	Statin vs non active	Secondary	Private	Life Years Gained	Euro (1995-1997)	1177	1673
23	Jönsson	1999	Statin vs non active	Secondary	Private	Life Years Gained	Euro (1995-1997)	1442	2050
23	Jönsson	1999	Statin vs non active	Secondary	Private	Life Years Gained	Euro (1995-1997)	1554	2209
23	Jönsson	1999	Statin vs non active	Secondary	Private	Life Years Gained	Euro (1995-1997)	1719	2443
23	Jönsson	1999	Statin vs non active	Secondary	Private	Life Years Gained	Euro (1995-1997)	2170	3084
23	Jönsson	1999	Statin vs non active	Secondary	Private	Life Years Gained	Euro (1995-1997)	2944	4185
23	Jönsson	1999	Statin vs non active	Secondary	Private	Life Years Gained	Euro (1995-1997)	4196	5964
23	Jönsson	1999	Statin vs non active	Secondary	Private	Life Years Gained	Euro (1995-1997)	6080	8642
23	Jönsson	1999	Statin vs non active	Secondary	Private	Life Years Gained	Euro (1995-1997)	6227	8851

23	Jönsson	1999	Statin vs non active	Secondary	Private	Life Years Gained	Euro (1995-1997)	6489	9223
23	Jönsson	1999	Statin vs non active	Secondary	Private	Life Years Gained	Euro (1995-1997)	6772	9626
23	Jönsson	1999	Statin vs non active	Secondary	Private	Life Years Gained	Euro (1995-1997)	6783	9641
23	Jönsson	1999	Statin vs non active	Secondary	Private	Life Years Gained	Euro (1995-1997)	7235	10284
23	Jönsson	1999	Statin vs non active	Secondary	Private	Life Years Gained	Euro (1995-1997)	7738	10999
23	Jönsson	1999	Statin vs non active	Secondary	Private	Life Years Gained	Euro (1995-1997)	7807	11097
23	Jönsson	1999	Statin vs non active	Secondary	Private	Life Years Gained	Euro (1995-1997)	9566	13597
24	Caro	1999	Statin vs non active	Primary	Private	Life Years Gained	$ UK (1997)	8150	8150
24	Caro	1999	Statin vs non active	Primary	Private	Life Years Gained	$ UK (1997)	8876	8876
24	Caro	1999	Statin vs non active	Primary	Private	Life Years Gained	$ UK (1997)	10999	10999
24	Caro	1999	Statin vs non active	Primary	Private	Life Years Gained	$ UK (1997)	13273	13273
24	Caro	1999	Statin vs non active	Primary	Private	Life Years Gained	$ UK (1997)	14773	14773
25	Grover	2000	Statin vs non active	Primary	Private	Life Years Gained	$ USA (1996)	7000	7000
25	Grover	2000	Statin vs non active	Primary	Private	Life Years Gained	$ USA (1996)	15000	15000
25	Grover	2000	Statin vs non active	Primary	Private	Life Years Gained	$ USA (1996)	5000	5000
25	Grover	2000	Statin vs non active	Primary	Private	Life Years Gained	$ USA (1996)	14000	14000
26	Prosser	2000	Statin vs non active	Secondary	Public or non-profit	Quality-Adjusted Life Years	$ USA (1997)	1800	1800
26	Prosser	2000	Statin vs non active	Secondary	Public or non-profit	Quality-Adjusted Life Years	$ USA (1997)	3900	3900
26	Prosser	2000	Statin vs non active	Secondary	Public or non-profit	Quality-Adjusted Life Years	$ USA (1997)	4500	4500
26	Prosser	2000	Statin vs non active	Secondary	Public or non-profit	Quality-Adjusted Life Years	$ USA (1997)	6700	6700
26	Prosser	2000	Statin vs non active	Secondary	Public or non-profit	Quality-Adjusted Life Years	$ USA (1997)	8100	8100
26	Prosser	2000	Statin vs non active	Secondary	Public or non-profit	Quality-Adjusted Life Years	$ USA (1997)	8400	8400
26	Prosser	2000	Statin vs non active	Secondary	Public or non-profit	Quality-Adjusted Life Years	$ USA (1997)	9500	9500
26	Prosser	2000	Statin vs non active	Secondary	Public or non-profit	Quality-Adjusted Life Years	$ USA (1997)	9900	9900
26	Prosser	2000	Statin vs non active	Secondary	Public or non-profit	Quality-Adjusted Life Years	$ USA (1997)	11000	11000
26	Prosser	2000	Statin vs non active	Secondary	Public or non-profit	Quality-Adjusted Life Years	$ USA (1997)	40000	40000
26	Prosser	2000	Statin vs non active	Primary	Public or non-profit	Quality-Adjusted Life Years	$ USA (1997)	54000	54000
26	Prosser	2000	Statin vs non active	Primary	Public or non-profit	Quality-Adjusted Life Years	$ USA (1997)	55000	55000
26	Prosser	2000	Statin vs non active	Primary	Public or non-profit	Quality-Adjusted Life Years	$ USA (1997)	55000	55000
26	Prosser	2000	Statin vs non active	Primary	Public or non-profit	Quality-Adjusted Life Years	$ USA (1997)	56000	56000
26	Prosser	2000	Statin vs non active	Primary	Public or non-profit	Quality-Adjusted Life Years	$ USA (1997)	56000	56000
26	Prosser	2000	Statin vs non active	Primary	Public or non-profit	Quality-Adjusted Life Years	$ USA (1997)	56000	56000
26	Prosser	2000	Statin vs non active	Primary	Public or non-profit	Quality-Adjusted Life Years	$ USA (1997)	57000	57000
26	Prosser	2000	Statin vs non active	Primary	Public or non-profit	Quality-Adjusted Life Years	$ USA (1997)	58000	58000
26	Prosser	2000	Statin vs non active	Primary	Public or non-profit	Quality-Adjusted Life Years	$ USA (1997)	59000	59000
26	Prosser	2000	Statin vs non active	Primary	Public or non-profit	Quality-Adjusted Life Years	$ USA (1997)	61000	61000
26	Prosser	2000	Statin vs non active	Primary	Public or non-profit	Quality-Adjusted Life Years	$ USA (1997)	62000	62000
26	Prosser	2000	Statin vs non active	Primary	Public or non-profit	Quality-Adjusted Life Years	$ USA (1997)	64000	64000
26	Prosser	2000	Statin vs non active	Primary	Public or non-profit	Quality-Adjusted Life Years	$ USA (1997)	66000	66000
26	Prosser	2000	Statin vs non active	Primary	Public or non-profit	Quality-Adjusted Life Years	$ USA (1997)	66000	66000
26	Prosser	2000	Statin vs non active	Primary	Public or non-profit	Quality-Adjusted Life Years	$ USA (1997)	69000	69000
26	Prosser	2000	Statin vs non active	Primary	Public or non-profit	Quality-Adjusted Life Years	$ USA (1997)	70000	70000
26	Prosser	2000	Statin vs non active	Primary	Public or non-profit	Quality-Adjusted Life Years	$ USA (1997)	73000	73000

26	Prosser	2000	Statin vs non active	Primary	Public or non-profit	Quality-Adjusted Life Years	$ USA (1997)	73000	73000
26	Prosser	2000	Statin vs non active	Primary	Public or non-profit	Quality-Adjusted Life Years	$ USA (1997)	73000	73000
26	Prosser	2000	Statin vs non active	Primary	Public or non-profit	Quality-Adjusted Life Years	$ USA (1997)	75000	75000
26	Prosser	2000	Statin vs non active	Primary	Public or non-profit	Quality-Adjusted Life Years	$ USA (1997)	77000	77000
26	Prosser	2000	Statin vs non active	Primary	Public or non-profit	Quality-Adjusted Life Years	$ USA (1997)	77000	77000
26	Prosser	2000	Statin vs non active	Primary	Public or non-profit	Quality-Adjusted Life Years	$ USA (1997)	78000	78000
26	Prosser	2000	Statin vs non active	Primary	Public or non-profit	Quality-Adjusted Life Years	$ USA (1997)	79000	79000
26	Prosser	2000	Statin vs non active	Primary	Public or non-profit	Quality-Adjusted Life Years	$ USA (1997)	80000	80000
26	Prosser	2000	Statin vs non active	Primary	Public or non-profit	Quality-Adjusted Life Years	$ USA (1997)	80000	80000
26	Prosser	2000	Statin vs non active	Primary	Public or non-profit	Quality-Adjusted Life Years	$ USA (1997)	81000	81000
26	Prosser	2000	Statin vs non active	Primary	Public or non-profit	Quality-Adjusted Life Years	$ USA (1997)	81000	81000
26	Prosser	2000	Statin vs non active	Primary	Public or non-profit	Quality-Adjusted Life Years	$ USA (1997)	84000	84000
26	Prosser	2000	Statin vs non active	Primary	Public or non-profit	Quality-Adjusted Life Years	$ USA (1997)	86000	86000
26	Prosser	2000	Statin vs non active	Primary	Public or non-profit	Quality-Adjusted Life Years	$ USA (1997)	87000	87000
26	Prosser	2000	Statin vs non active	Primary	Public or non-profit	Quality-Adjusted Life Years	$ USA (1997)	87000	87000
26	Prosser	2000	Statin vs non active	Primary	Public or non-profit	Quality-Adjusted Life Years	$ USA (1997)	88000	88000
26	Prosser	2000	Statin vs non active	Primary	Public or non-profit	Quality-Adjusted Life Years	$ USA (1997)	88000	88000
26	Prosser	2000	Statin vs non active	Primary	Public or non-profit	Quality-Adjusted Life Years	$ USA (1997)	88000	88000
26	Prosser	2000	Statin vs non active	Primary	Public or non-profit	Quality-Adjusted Life Years	$ USA (1997)	89000	89000
26	Prosser	2000	Statin vs non active	Primary	Public or non-profit	Quality-Adjusted Life Years	$ USA (1997)	90000	90000
26	Prosser	2000	Statin vs non active	Primary	Public or non-profit	Quality-Adjusted Life Years	$ USA (1997)	91000	91000
26	Prosser	2000	Statin vs non active	Primary	Public or non-profit	Quality-Adjusted Life Years	$ USA (1997)	92000	92000
26	Prosser	2000	Statin vs non active	Primary	Public or non-profit	Quality-Adjusted Life Years	$ USA (1997)	93000	93000
26	Prosser	2000	Statin vs non active	Primary	Public or non-profit	Quality-Adjusted Life Years	$ USA (1997)	93000	93000
26	Prosser	2000	Statin vs non active	Primary	Public or non-profit	Quality-Adjusted Life Years	$ USA (1997)	94000	94000
26	Prosser	2000	Statin vs non active	Primary	Public or non-profit	Quality-Adjusted Life Years	$ USA (1997)	94000	94000
26	Prosser	2000	Statin vs non active	Primary	Public or non-profit	Quality-Adjusted Life Years	$ USA (1997)	94000	94000
26	Prosser	2000	Statin vs non active	Primary	Public or non-profit	Quality-Adjusted Life Years	$ USA (1997)	95000	95000
26	Prosser	2000	Statin vs non active	Primary	Public or non-profit	Quality-Adjusted Life Years	$ USA (1997)	95000	95000
26	Prosser	2000	Statin vs non active	Primary	Public or non-profit	Quality-Adjusted Life Years	$ USA (1997)	96000	96000
26	Prosser	2000	Statin vs non active	Primary	Public or non-profit	Quality-Adjusted Life Years	$ USA (1997)	96000	96000
26	Prosser	2000	Statin vs non active	Primary	Public or non-profit	Quality-Adjusted Life Years	$ USA (1997)	96000	96000
26	Prosser	2000	Statin vs non active	Primary	Public or non-profit	Quality-Adjusted Life Years	$ USA (1997)	98000	98000
26	Prosser	2000	Statin vs non active	Primary	Public or non-profit	Quality-Adjusted Life Years	$ USA (1997)	99000	99000
26	Prosser	2000	Statin vs non active	Primary	Public or non-profit	Quality-Adjusted Life Years	$ USA (1997)	100000	100000
26	Prosser	2000	Statin vs non active	Primary	Public or non-profit	Quality-Adjusted Life Years	$ USA (1997)	100000	100000
26	Prosser	2000	Statin vs non active	Primary	Public or non-profit	Quality-Adjusted Life Years	$ USA (1997)	100000	100000
26	Prosser	2000	Statin vs non active	Primary	Public or non-profit	Quality-Adjusted Life Years	$ USA (1997)	100000	100000
26	Prosser	2000	Statin vs non active	Primary	Public or non-profit	Quality-Adjusted Life Years	$ USA (1997)	100000	100000
26	Prosser	2000	Statin vs non active	Primary	Public or non-profit	Quality-Adjusted Life Years	$ USA (1997)	100000	100000
26	Prosser	2000	Statin vs non active	Primary	Public or non-profit	Quality-Adjusted Life Years	$ USA (1997)	100000	100000
26	Prosser	2000	Statin vs non active	Primary	Public or non-profit	Quality-Adjusted Life Years	$ USA (1997)	100000	100000
26	Prosser	2000	Statin vs non active	Primary	Public or non-profit	Quality-Adjusted Life Years	$ USA (1997)	100000	100000

26	Prosser	2000	Statin vs non active	Primary	Public or non-profit	Quality-Adjusted Life Years	$ USA (1997)	100000	100000
26	Prosser	2000	Statin vs non active	Primary	Public or non-profit	Quality-Adjusted Life Years	$ USA (1997)	100000	100000
26	Prosser	2000	Statin vs non active	Primary	Public or non-profit	Quality-Adjusted Life Years	$ USA (1997)	100000	100000
26	Prosser	2000	Statin vs non active	Primary	Public or non-profit	Quality-Adjusted Life Years	$ USA (1997)	110000	110000
26	Prosser	2000	Statin vs non active	Primary	Public or non-profit	Quality-Adjusted Life Years	$ USA (1997)	110000	110000
26	Prosser	2000	Statin vs non active	Primary	Public or non-profit	Quality-Adjusted Life Years	$ USA (1997)	110000	110000
26	Prosser	2000	Statin vs non active	Primary	Public or non-profit	Quality-Adjusted Life Years	$ USA (1997)	110000	110000
26	Prosser	2000	Statin vs non active	Primary	Public or non-profit	Quality-Adjusted Life Years	$ USA (1997)	110000	110000
26	Prosser	2000	Statin vs non active	Primary	Public or non-profit	Quality-Adjusted Life Years	$ USA (1997)	110000	110000
26	Prosser	2000	Statin vs non active	Primary	Public or non-profit	Quality-Adjusted Life Years	$ USA (1997)	110000	110000
26	Prosser	2000	Statin vs non active	Primary	Public or non-profit	Quality-Adjusted Life Years	$ USA (1997)	110000	110000
26	Prosser	2000	Statin vs non active	Primary	Public or non-profit	Quality-Adjusted Life Years	$ USA (1997)	110000	110000
26	Prosser	2000	Statin vs non active	Primary	Public or non-profit	Quality-Adjusted Life Years	$ USA (1997)	110000	110000
26	Prosser	2000	Statin vs non active	Primary	Public or non-profit	Quality-Adjusted Life Years	$ USA (1997)	110000	110000
26	Prosser	2000	Statin vs non active	Primary	Public or non-profit	Quality-Adjusted Life Years	$ USA (1997)	110000	110000
26	Prosser	2000	Statin vs non active	Primary	Public or non-profit	Quality-Adjusted Life Years	$ USA (1997)	110000	110000
26	Prosser	2000	Statin vs non active	Primary	Public or non-profit	Quality-Adjusted Life Years	$ USA (1997)	110000	110000
26	Prosser	2000	Statin vs non active	Primary	Public or non-profit	Quality-Adjusted Life Years	$ USA (1997)	110000	110000
26	Prosser	2000	Statin vs non active	Primary	Public or non-profit	Quality-Adjusted Life Years	$ USA (1997)	120000	120000
26	Prosser	2000	Statin vs non active	Primary	Public or non-profit	Quality-Adjusted Life Years	$ USA (1997)	120000	120000
26	Prosser	2000	Statin vs non active	Primary	Public or non-profit	Quality-Adjusted Life Years	$ USA (1997)	120000	120000
26	Prosser	2000	Statin vs non active	Primary	Public or non-profit	Quality-Adjusted Life Years	$ USA (1997)	120000	120000
26	Prosser	2000	Statin vs non active	Primary	Public or non-profit	Quality-Adjusted Life Years	$ USA (1997)	120000	120000
26	Prosser	2000	Statin vs non active	Primary	Public or non-profit	Quality-Adjusted Life Years	$ USA (1997)	120000	120000
26	Prosser	2000	Statin vs non active	Primary	Public or non-profit	Quality-Adjusted Life Years	$ USA (1997)	120000	120000
26	Prosser	2000	Statin vs non active	Primary	Public or non-profit	Quality-Adjusted Life Years	$ USA (1997)	120000	120000
26	Prosser	2000	Statin vs non active	Primary	Public or non-profit	Quality-Adjusted Life Years	$ USA (1997)	120000	120000
26	Prosser	2000	Statin vs non active	Primary	Public or non-profit	Quality-Adjusted Life Years	$ USA (1997)	120000	120000
26	Prosser	2000	Statin vs non active	Primary	Public or non-profit	Quality-Adjusted Life Years	$ USA (1997)	120000	120000
26	Prosser	2000	Statin vs non active	Primary	Public or non-profit	Quality-Adjusted Life Years	$ USA (1997)	120000	120000
26	Prosser	2000	Statin vs non active	Primary	Public or non-profit	Quality-Adjusted Life Years	$ USA (1997)	120000	120000
26	Prosser	2000	Statin vs non active	Primary	Public or non-profit	Quality-Adjusted Life Years	$ USA (1997)	120000	120000
26	Prosser	2000	Statin vs non active	Primary	Public or non-profit	Quality-Adjusted Life Years	$ USA (1997)	120000	120000
26	Prosser	2000	Statin vs non active	Primary	Public or non-profit	Quality-Adjusted Life Years	$ USA (1997)	130000	130000
26	Prosser	2000	Statin vs non active	Primary	Public or non-profit	Quality-Adjusted Life Years	$ USA (1997)	130000	130000
26	Prosser	2000	Statin vs non active	Primary	Public or non-profit	Quality-Adjusted Life Years	$ USA (1997)	130000	130000
26	Prosser	2000	Statin vs non active	Primary	Public or non-profit	Quality-Adjusted Life Years	$ USA (1997)	130000	130000
26	Prosser	2000	Statin vs non active	Primary	Public or non-profit	Quality-Adjusted Life Years	$ USA (1997)	130000	130000
26	Prosser	2000	Statin vs non active	Primary	Public or non-profit	Quality-Adjusted Life Years	$ USA (1997)	130000	130000
26	Prosser	2000	Statin vs non active	Primary	Public or non-profit	Quality-Adjusted Life Years	$ USA (1997)	130000	130000
26	Prosser	2000	Statin vs non active	Primary	Public or non-profit	Quality-Adjusted Life Years	$ USA (1997)	130000	130000
26	Prosser	2000	Statin vs non active	Primary	Public or non-profit	Quality-Adjusted Life Years	$ USA (1997)	130000	130000
26	Prosser	2000	Statin vs non active	Primary	Public or non-profit	Quality-Adjusted Life Years	$ USA (1997)	140000	140000

26	Prosser	2000	Statin vs non active	Primary	Public or non-profit	Quality-Adjusted Life Years	$ USA (1997)	140000	140000
26	Prosser	2000	Statin vs non active	Primary	Public or non-profit	Quality-Adjusted Life Years	$ USA (1997)	140000	140000
26	Prosser	2000	Statin vs non active	Primary	Public or non-profit	Quality-Adjusted Life Years	$ USA (1997)	140000	140000
26	Prosser	2000	Statin vs non active	Primary	Public or non-profit	Quality-Adjusted Life Years	$ USA (1997)	140000	140000
26	Prosser	2000	Statin vs non active	Primary	Public or non-profit	Quality-Adjusted Life Years	$ USA (1997)	140000	140000
26	Prosser	2000	Statin vs non active	Primary	Public or non-profit	Quality-Adjusted Life Years	$ USA (1997)	140000	140000
26	Prosser	2000	Statin vs non active	Primary	Public or non-profit	Quality-Adjusted Life Years	$ USA (1997)	140000	140000
26	Prosser	2000	Statin vs non active	Primary	Public or non-profit	Quality-Adjusted Life Years	$ USA (1997)	140000	140000
26	Prosser	2000	Statin vs non active	Primary	Public or non-profit	Quality-Adjusted Life Years	$ USA (1997)	140000	140000
26	Prosser	2000	Statin vs non active	Primary	Public or non-profit	Quality-Adjusted Life Years	$ USA (1997)	150000	150000
26	Prosser	2000	Statin vs non active	Primary	Public or non-profit	Quality-Adjusted Life Years	$ USA (1997)	150000	150000
26	Prosser	2000	Statin vs non active	Primary	Public or non-profit	Quality-Adjusted Life Years	$ USA (1997)	150000	150000
26	Prosser	2000	Statin vs non active	Primary	Public or non-profit	Quality-Adjusted Life Years	$ USA (1997)	150000	150000
26	Prosser	2000	Statin vs non active	Primary	Public or non-profit	Quality-Adjusted Life Years	$ USA (1997)	150000	150000
26	Prosser	2000	Statin vs non active	Primary	Public or non-profit	Quality-Adjusted Life Years	$ USA (1997)	150000	150000
26	Prosser	2000	Statin vs non active	Primary	Public or non-profit	Quality-Adjusted Life Years	$ USA (1997)	150000	150000
26	Prosser	2000	Statin vs non active	Primary	Public or non-profit	Quality-Adjusted Life Years	$ USA (1997)	150000	150000
26	Prosser	2000	Statin vs non active	Primary	Public or non-profit	Quality-Adjusted Life Years	$ USA (1997)	150000	150000
26	Prosser	2000	Statin vs non active	Primary	Public or non-profit	Quality-Adjusted Life Years	$ USA (1997)	150000	150000
26	Prosser	2000	Statin vs non active	Primary	Public or non-profit	Quality-Adjusted Life Years	$ USA (1997)	150000	150000
26	Prosser	2000	Statin vs non active	Primary	Public or non-profit	Quality-Adjusted Life Years	$ USA (1997)	150000	150000
26	Prosser	2000	Statin vs non active	Primary	Public or non-profit	Quality-Adjusted Life Years	$ USA (1997)	160000	160000
26	Prosser	2000	Statin vs non active	Primary	Public or non-profit	Quality-Adjusted Life Years	$ USA (1997)	160000	160000
26	Prosser	2000	Statin vs non active	Primary	Public or non-profit	Quality-Adjusted Life Years	$ USA (1997)	160000	160000
26	Prosser	2000	Statin vs non active	Primary	Public or non-profit	Quality-Adjusted Life Years	$ USA (1997)	160000	160000
26	Prosser	2000	Statin vs non active	Primary	Public or non-profit	Quality-Adjusted Life Years	$ USA (1997)	160000	160000
26	Prosser	2000	Statin vs non active	Primary	Public or non-profit	Quality-Adjusted Life Years	$ USA (1997)	160000	160000
26	Prosser	2000	Statin vs non active	Primary	Public or non-profit	Quality-Adjusted Life Years	$ USA (1997)	160000	160000
26	Prosser	2000	Statin vs non active	Primary	Public or non-profit	Quality-Adjusted Life Years	$ USA (1997)	160000	160000
26	Prosser	2000	Statin vs non active	Primary	Public or non-profit	Quality-Adjusted Life Years	$ USA (1997)	160000	160000
26	Prosser	2000	Statin vs non active	Primary	Public or non-profit	Quality-Adjusted Life Years	$ USA (1997)	160000	160000
26	Prosser	2000	Statin vs non active	Primary	Public or non-profit	Quality-Adjusted Life Years	$ USA (1997)	160000	160000
26	Prosser	2000	Statin vs non active	Primary	Public or non-profit	Quality-Adjusted Life Years	$ USA (1997)	160000	160000
26	Prosser	2000	Statin vs non active	Primary	Public or non-profit	Quality-Adjusted Life Years	$ USA (1997)	160000	160000
26	Prosser	2000	Statin vs non active	Primary	Public or non-profit	Quality-Adjusted Life Years	$ USA (1997)	160000	160000
26	Prosser	2000	Statin vs non active	Primary	Public or non-profit	Quality-Adjusted Life Years	$ USA (1997)	160000	160000
26	Prosser	2000	Statin vs non active	Primary	Public or non-profit	Quality-Adjusted Life Years	$ USA (1997)	160000	160000
26	Prosser	2000	Statin vs non active	Primary	Public or non-profit	Quality-Adjusted Life Years	$ USA (1997)	170000	170000
26	Prosser	2000	Statin vs non active	Primary	Public or non-profit	Quality-Adjusted Life Years	$ USA (1997)	170000	170000
26	Prosser	2000	Statin vs non active	Primary	Public or non-profit	Quality-Adjusted Life Years	$ USA (1997)	170000	170000
26	Prosser	2000	Statin vs non active	Primary	Public or non-profit	Quality-Adjusted Life Years	$ USA (1997)	170000	170000
26	Prosser	2000	Statin vs non active	Primary	Public or non-profit	Quality-Adjusted Life Years	$ USA (1997)	170000	170000
26	Prosser	2000	Statin vs non active	Primary	Public or non-profit	Quality-Adjusted Life Years	$ USA (1997)	170000	170000

26	Prosser	2000	Statin vs non active	Primary	Public or non-profit	Quality-Adjusted Life Years	$ USA (1997)	170000	170000
26	Prosser	2000	Statin vs non active	Primary	Public or non-profit	Quality-Adjusted Life Years	$ USA (1997)	180000	180000
26	Prosser	2000	Statin vs non active	Primary	Public or non-profit	Quality-Adjusted Life Years	$ USA (1997)	180000	180000
26	Prosser	2000	Statin vs non active	Primary	Public or non-profit	Quality-Adjusted Life Years	$ USA (1997)	180000	180000
26	Prosser	2000	Statin vs non active	Primary	Public or non-profit	Quality-Adjusted Life Years	$ USA (1997)	180000	180000
26	Prosser	2000	Statin vs non active	Primary	Public or non-profit	Quality-Adjusted Life Years	$ USA (1997)	180000	180000
26	Prosser	2000	Statin vs non active	Primary	Public or non-profit	Quality-Adjusted Life Years	$ USA (1997)	180000	180000
26	Prosser	2000	Statin vs non active	Primary	Public or non-profit	Quality-Adjusted Life Years	$ USA (1997)	180000	180000
26	Prosser	2000	Statin vs non active	Primary	Public or non-profit	Quality-Adjusted Life Years	$ USA (1997)	190000	190000
26	Prosser	2000	Statin vs non active	Primary	Public or non-profit	Quality-Adjusted Life Years	$ USA (1997)	190000	190000
26	Prosser	2000	Statin vs non active	Primary	Public or non-profit	Quality-Adjusted Life Years	$ USA (1997)	190000	190000
26	Prosser	2000	Statin vs non active	Primary	Public or non-profit	Quality-Adjusted Life Years	$ USA (1997)	190000	190000
26	Prosser	2000	Statin vs non active	Primary	Public or non-profit	Quality-Adjusted Life Years	$ USA (1997)	190000	190000
26	Prosser	2000	Statin vs non active	Primary	Public or non-profit	Quality-Adjusted Life Years	$ USA (1997)	190000	190000
26	Prosser	2000	Statin vs non active	Primary	Public or non-profit	Quality-Adjusted Life Years	$ USA (1997)	190000	190000
26	Prosser	2000	Statin vs non active	Primary	Public or non-profit	Quality-Adjusted Life Years	$ USA (1997)	200000	200000
26	Prosser	2000	Statin vs non active	Primary	Public or non-profit	Quality-Adjusted Life Years	$ USA (1997)	200000	200000
26	Prosser	2000	Statin vs non active	Primary	Public or non-profit	Quality-Adjusted Life Years	$ USA (1997)	200000	200000
26	Prosser	2000	Statin vs non active	Primary	Public or non-profit	Quality-Adjusted Life Years	$ USA (1997)	200000	200000
26	Prosser	2000	Statin vs non active	Primary	Public or non-profit	Quality-Adjusted Life Years	$ USA (1997)	200000	200000
26	Prosser	2000	Statin vs non active	Primary	Public or non-profit	Quality-Adjusted Life Years	$ USA (1997)	210000	210000
26	Prosser	2000	Statin vs non active	Primary	Public or non-profit	Quality-Adjusted Life Years	$ USA (1997)	210000	210000
26	Prosser	2000	Statin vs non active	Primary	Public or non-profit	Quality-Adjusted Life Years	$ USA (1997)	210000	210000
26	Prosser	2000	Statin vs non active	Primary	Public or non-profit	Quality-Adjusted Life Years	$ USA (1997)	210000	210000
26	Prosser	2000	Statin vs non active	Primary	Public or non-profit	Quality-Adjusted Life Years	$ USA (1997)	210000	210000
26	Prosser	2000	Statin vs non active	Primary	Public or non-profit	Quality-Adjusted Life Years	$ USA (1997)	210000	210000
26	Prosser	2000	Statin vs non active	Primary	Public or non-profit	Quality-Adjusted Life Years	$ USA (1997)	220000	220000
26	Prosser	2000	Statin vs non active	Primary	Public or non-profit	Quality-Adjusted Life Years	$ USA (1997)	220000	220000
26	Prosser	2000	Statin vs non active	Primary	Public or non-profit	Quality-Adjusted Life Years	$ USA (1997)	220000	220000
26	Prosser	2000	Statin vs non active	Primary	Public or non-profit	Quality-Adjusted Life Years	$ USA (1997)	220000	220000
26	Prosser	2000	Statin vs non active	Primary	Public or non-profit	Quality-Adjusted Life Years	$ USA (1997)	220000	220000
26	Prosser	2000	Statin vs non active	Primary	Public or non-profit	Quality-Adjusted Life Years	$ USA (1997)	220000	220000
26	Prosser	2000	Statin vs non active	Primary	Public or non-profit	Quality-Adjusted Life Years	$ USA (1997)	220000	220000
26	Prosser	2000	Statin vs non active	Primary	Public or non-profit	Quality-Adjusted Life Years	$ USA (1997)	230000	230000
26	Prosser	2000	Statin vs non active	Primary	Public or non-profit	Quality-Adjusted Life Years	$ USA (1997)	230000	230000
26	Prosser	2000	Statin vs non active	Primary	Public or non-profit	Quality-Adjusted Life Years	$ USA (1997)	230000	230000
26	Prosser	2000	Statin vs non active	Primary	Public or non-profit	Quality-Adjusted Life Years	$ USA (1997)	240000	240000
26	Prosser	2000	Statin vs non active	Primary	Public or non-profit	Quality-Adjusted Life Years	$ USA (1997)	240000	240000
26	Prosser	2000	Statin vs non active	Primary	Public or non-profit	Quality-Adjusted Life Years	$ USA (1997)	240000	240000
26	Prosser	2000	Statin vs non active	Primary	Public or non-profit	Quality-Adjusted Life Years	$ USA (1997)	250000	250000
26	Prosser	2000	Statin vs non active	Primary	Public or non-profit	Quality-Adjusted Life Years	$ USA (1997)	250000	250000
26	Prosser	2000	Statin vs non active	Primary	Public or non-profit	Quality-Adjusted Life Years	$ USA (1997)	250000	250000
26	Prosser	2000	Statin vs non active	Primary	Public or non-profit	Quality-Adjusted Life Years	$ USA (1997)	250000	250000

26	Prosser	2000	Statin vs non active	Primary	Public or non-profit	Quality-Adjusted Life Years	$ USA (1997)	250000	250000
26	Prosser	2000	Statin vs non active	Primary	Public or non-profit	Quality-Adjusted Life Years	$ USA (1997)	260000	260000
26	Prosser	2000	Statin vs non active	Primary	Public or non-profit	Quality-Adjusted Life Years	$ USA (1997)	270000	270000
26	Prosser	2000	Statin vs non active	Primary	Public or non-profit	Quality-Adjusted Life Years	$ USA (1997)	270000	270000
26	Prosser	2000	Statin vs non active	Primary	Public or non-profit	Quality-Adjusted Life Years	$ USA (1997)	270000	270000
26	Prosser	2000	Statin vs non active	Primary	Public or non-profit	Quality-Adjusted Life Years	$ USA (1997)	270000	270000
26	Prosser	2000	Statin vs non active	Primary	Public or non-profit	Quality-Adjusted Life Years	$ USA (1997)	280000	280000
26	Prosser	2000	Statin vs non active	Primary	Public or non-profit	Quality-Adjusted Life Years	$ USA (1997)	290000	290000
26	Prosser	2000	Statin vs non active	Primary	Public or non-profit	Quality-Adjusted Life Years	$ USA (1997)	290000	290000
26	Prosser	2000	Statin vs non active	Primary	Public or non-profit	Quality-Adjusted Life Years	$ USA (1997)	290000	290000
26	Prosser	2000	Statin vs non active	Primary	Public or non-profit	Quality-Adjusted Life Years	$ USA (1997)	290000	290000
26	Prosser	2000	Statin vs non active	Primary	Public or non-profit	Quality-Adjusted Life Years	$ USA (1997)	300000	300000
26	Prosser	2000	Statin vs non active	Primary	Public or non-profit	Quality-Adjusted Life Years	$ USA (1997)	300000	300000
26	Prosser	2000	Statin vs non active	Primary	Public or non-profit	Quality-Adjusted Life Years	$ USA (1997)	310000	310000
26	Prosser	2000	Statin vs non active	Primary	Public or non-profit	Quality-Adjusted Life Years	$ USA (1997)	310000	310000
26	Prosser	2000	Statin vs non active	Primary	Public or non-profit	Quality-Adjusted Life Years	$ USA (1997)	310000	310000
26	Prosser	2000	Statin vs non active	Primary	Public or non-profit	Quality-Adjusted Life Years	$ USA (1997)	320000	320000
26	Prosser	2000	Statin vs non active	Primary	Public or non-profit	Quality-Adjusted Life Years	$ USA (1997)	320000	320000
26	Prosser	2000	Statin vs non active	Primary	Public or non-profit	Quality-Adjusted Life Years	$ USA (1997)	330000	330000
26	Prosser	2000	Statin vs non active	Primary	Public or non-profit	Quality-Adjusted Life Years	$ USA (1997)	350000	350000
26	Prosser	2000	Statin vs non active	Primary	Public or non-profit	Quality-Adjusted Life Years	$ USA (1997)	360000	360000
26	Prosser	2000	Statin vs non active	Primary	Public or non-profit	Quality-Adjusted Life Years	$ USA (1997)	360000	360000
26	Prosser	2000	Statin vs non active	Primary	Public or non-profit	Quality-Adjusted Life Years	$ USA (1997)	370000	370000
26	Prosser	2000	Statin vs non active	Primary	Public or non-profit	Quality-Adjusted Life Years	$ USA (1997)	380000	380000
26	Prosser	2000	Statin vs non active	Primary	Public or non-profit	Quality-Adjusted Life Years	$ USA (1997)	390000	390000
26	Prosser	2000	Statin vs non active	Primary	Public or non-profit	Quality-Adjusted Life Years	$ USA (1997)	390000	390000
26	Prosser	2000	Statin vs non active	Primary	Public or non-profit	Quality-Adjusted Life Years	$ USA (1997)	390000	390000
26	Prosser	2000	Statin vs non active	Primary	Public or non-profit	Quality-Adjusted Life Years	$ USA (1997)	390000	390000
26	Prosser	2000	Statin vs non active	Primary	Public or non-profit	Quality-Adjusted Life Years	$ USA (1997)	400000	400000
26	Prosser	2000	Statin vs non active	Primary	Public or non-profit	Quality-Adjusted Life Years	$ USA (1997)	410000	410000
26	Prosser	2000	Statin vs non active	Primary	Public or non-profit	Quality-Adjusted Life Years	$ USA (1997)	410000	410000
26	Prosser	2000	Statin vs non active	Primary	Public or non-profit	Quality-Adjusted Life Years	$ USA (1997)	420000	420000
26	Prosser	2000	Statin vs non active	Primary	Public or non-profit	Quality-Adjusted Life Years	$ USA (1997)	420000	420000
26	Prosser	2000	Statin vs non active	Primary	Public or non-profit	Quality-Adjusted Life Years	$ USA (1997)	420000	420000
26	Prosser	2000	Statin vs non active	Primary	Public or non-profit	Quality-Adjusted Life Years	$ USA (1997)	420000	420000
26	Prosser	2000	Statin vs non active	Primary	Public or non-profit	Quality-Adjusted Life Years	$ USA (1997)	460000	460000
26	Prosser	2000	Statin vs non active	Primary	Public or non-profit	Quality-Adjusted Life Years	$ USA (1997)	470000	470000
26	Prosser	2000	Statin vs non active	Primary	Public or non-profit	Quality-Adjusted Life Years	$ USA (1997)	480000	480000
26	Prosser	2000	Statin vs non active	Primary	Public or non-profit	Quality-Adjusted Life Years	$ USA (1997)	500000	500000
26	Prosser	2000	Statin vs non active	Primary	Public or non-profit	Quality-Adjusted Life Years	$ USA (1997)	550000	550000
26	Prosser	2000	Statin vs non active	Primary	Public or non-profit	Quality-Adjusted Life Years	$ USA (1997)	560000	560000
26	Prosser	2000	Statin vs non active	Primary	Public or non-profit	Quality-Adjusted Life Years	$ USA (1997)	600000	600000
26	Prosser	2000	Statin vs non active	Primary	Public or non-profit	Quality-Adjusted Life Years	$ USA (1997)	620000	620000

26	Prosser	2000	Statin vs non active	Primary	Public or non-profit	Quality-Adjusted Life Years	$ USA (1997)	660000	660000
26	Prosser	2000	Statin vs non active	Primary	Public or non-profit	Quality-Adjusted Life Years	$ USA (1997)	680000	680000
26	Prosser	2000	Statin vs non active	Primary	Public or non-profit	Quality-Adjusted Life Years	$ USA (1997)	690000	690000
26	Prosser	2000	Statin vs non active	Primary	Public or non-profit	Quality-Adjusted Life Years	$ USA (1997)	690000	690000
26	Prosser	2000	Statin vs non active	Primary	Public or non-profit	Quality-Adjusted Life Years	$ USA (1997)	930000	930000
26	Prosser	2000	Statin vs non active	Primary	Public or non-profit	Quality-Adjusted Life Years	$ USA (1997)	960000	960000
26	Prosser	2000	Statin vs non active	Primary	Public or non-profit	Quality-Adjusted Life Years	$ USA (1997)	1400000	1400000
26	Prosser	2000	Statin vs non active	Primary	Public or non-profit	Quality-Adjusted Life Years	$ USA (1997)	1400000	1400000
27	Caro	2000	Statin vs non active	Primary	Private	Life Years Gained	Euro (1998)	29900	42500
28	Ganz	2000	Statin vs non active	Secondary	Public or non-profit	Quality-Adjusted Life Years	$ USA (1998)	18800	18800
29	Shepherd	2001	Statin vs non active	Primary	None	Life Years Gained	£ UK (1996)	13995	22583
30	Lim	2001	Statin vs non active	Primary	Public or non-profit	Life Years Gained	$ Australian (1999)	23000	24691
30	Lim	2001	Statin vs non active	Primary	Public or non-profit	Life Years Gained	$ Australian (1999)	31000	33279
30	Lim	2001	Statin vs non active	Primary	Public or non-profit	Life Years Gained	$ Australian (1999)	37000	39720
30	Lim	2001	Statin vs non active	Primary	Public or non-profit	Life Years Gained	$ Australian (1999)	39000	41867
30	Lim	2001	Statin vs non active	Primary	Public or non-profit	Life Years Gained	$ Australian (1999)	87000	93395
30	Lim	2001	Statin vs non active	Primary	Public or non-profit	Life Years Gained	$ Australian (1999)	110000	118086
31	Grover	2001	Statin vs non active	Primary	Private	Life Years Gained	$ USA (1998)	5063	5063
31	Grover	2001	Statin vs non active	Primary	Private	Life Years Gained	$ USA (1998)	5740	5740
31	Grover	2001	Statin vs non active	Primary	Private	Life Years Gained	$ USA (1998)	6017	6017
31	Grover	2001	Statin vs non active	Secondary	Private	Life Years Gained	$ USA (1998)	8799	8799
31	Grover	2001	Statin vs non active	Secondary	Private	Life Years Gained	$ USA (1998)	9513	9513
31	Grover	2001	Statin vs non active	Secondary	Private	Life Years Gained	$ USA (1998)	12111	12111
31	Grover	2001	Statin vs non active	Primary	Private	Life Years Gained	$ USA (1998)	13121	13121
31	Grover	2001	Statin vs non active	Primary	Private	Life Years Gained	$ USA (1998)	14156	14156
31	Grover	2001	Statin vs non active	Secondary	Private	Life Years Gained	$ USA (1998)	14164	14164
31	Grover	2001	Statin vs non active	Primary	Private	Life Years Gained	$ USA (1998)	14481	14481
31	Grover	2001	Statin vs non active	Secondary	Private	Life Years Gained	$ USA (1998)	14996	14996
31	Grover	2001	Statin vs non active	Secondary	Private	Life Years Gained	$ USA (1998)	16020	16020
31	Grover	2001	Statin vs non active	Primary	Private	Life Years Gained	$ USA (1998)	19019	19019
31	Grover	2001	Statin vs non active	Secondary	Private	Life Years Gained	$ USA (1998)	20494	20494
31	Grover	2001	Statin vs non active	Secondary	Private	Life Years Gained	$ USA (1998)	21628	21628
31	Grover	2001	Statin vs non active	Primary	Private	Life Years Gained	$ USA (1998)	23792	23792
32	Tsevat	2001	Statin vs non active	Secondary	Private	Quality-Adjusted Life Years	$ USA (1996)	16000	16000
32	Tsevat	2001	Statin vs non active	Secondary	Private	Quality-Adjusted Life Years	$ USA (1996)	22000	22000
32	Tsevat	2001	Statin vs non active	Secondary	Private	Quality-Adjusted Life Years	$ USA (1996)	31000	31000
32	Tsevat	2001	Statin vs non active	Secondary	Private	Quality-Adjusted Life Years	$ USA (1996)	32000	32000
33	Chau	2001	Statin vs non active	Secondary	None	Quality-Adjusted Life Years	$ HK (1997)	73218	9152
34	van Hout	2001	Statin vs non active	Secondary	None	Life Years Gained	Euro (1999)	6695	9516
34	van Hout	2001	Statin vs non active	Secondary	None	Life Years Gained	Euro (1999)	8028	11411
34	van Hout	2001	Statin vs non active	Secondary	None	Life Years Gained	Euro (1999)	9970	14171
34	van Hout	2001	Statin vs non active	Primary	None	Life Years Gained	Euro (1999)	26013	36975
34	van Hout	2001	Statin vs non active	Primary	None	Life Years Gained	Euro (1999)	51400	73060

35	Russell	2001	Statin vs statin	Primary	Private		Life Years Gained	$ Canadian (1997)	6904	7194
35	Russell	2001	Statin vs statin	Primary	Private		Life Years Gained	$ Canadian (1997)	8242	8588
35	Russell	2001	Statin vs statin	Secondary	Private		Life Years Gained	$ Canadian (1997)	9655	10061
35	Russell	2001	Statin vs statin	Primary	Private		Life Years Gained	$ Canadian (1997)	9832	10245
35	Russell	2001	Statin vs statin	Secondary	Private		Life Years Gained	$ Canadian (1997)	10128	10554
35	Russell	2001	Statin vs statin	Secondary	Private		Life Years Gained	$ Canadian (1997)	10470	10910
35	Russell	2001	Statin vs statin	Primary	Private		Life Years Gained	$ Canadian (1997)	11073	11538
35	Russell	2001	Statin vs non active	Primary	Private		Life Years Gained	$ Canadian (1997)	11559	12045
35	Russell	2001	Statin vs statin	Primary	Private		Life Years Gained	$ Canadian (1997)	11803	12299
35	Russell	2001	Statin vs statin	Secondary	Private		Life Years Gained	$ Canadian (1997)	12069	12576
35	Russell	2001	Statin vs statin	Secondary	Private		Life Years Gained	$ Canadian (1997)	12333	12851
35	Russell	2001	Statin vs statin	Secondary	Private		Life Years Gained	$ Canadian (1997)	12888	13430
35	Russell	2001	Statin vs non active	Primary	Private		Life Years Gained	$ Canadian (1997)	13010	13557
35	Russell	2001	Statin vs statin	Secondary	Private		Life Years Gained	$ Canadian (1997)	13158	13711
35	Russell	2001	Statin vs statin	Secondary	Private		Life Years Gained	$ Canadian (1997)	13214	13769
35	Russell	2001	Statin vs non active	Secondary	Private		Life Years Gained	$ Canadian (1997)	14128	14722
35	Russell	2001	Statin vs statin	Secondary	Private		Life Years Gained	$ Canadian (1997)	14321	14923
35	Russell	2001	Statin vs statin	Secondary	Private		Life Years Gained	$ Canadian (1997)	14351	14954
35	Russell	2001	Statin vs statin	Secondary	Private		Life Years Gained	$ Canadian (1997)	14618	15232
35	Russell	2001	Statin vs non active	Secondary	Private		Life Years Gained	$ Canadian (1997)	15038	15670
35	Russell	2001	Statin vs non active	Secondary	Private		Life Years Gained	$ Canadian (1997)	15493	16144
35	Russell	2001	Statin vs statin	Secondary	Private		Life Years Gained	$ Canadian (1997)	15778	16441
35	Russell	2001	Statin vs non active	Primary	Private		Life Years Gained	$ Canadian (1997)	15820	16485
35	Russell	2001	Statin vs statin	Primary	Private		Life Years Gained	$ Canadian (1997)	15902	16570
35	Russell	2001	Statin vs non active	Primary	Private		Life Years Gained	$ Canadian (1997)	16281	16965
35	Russell	2001	Statin vs statin	Primary	Private		Life Years Gained	$ Canadian (1997)	16726	17429
35	Russell	2001	Statin vs non active	Primary	Private		Life Years Gained	$ Canadian (1997)	16898	17608
35	Russell	2001	Statin vs non active	Primary	Private		Life Years Gained	$ Canadian (1997)	17429	18161
35	Russell	2001	Statin vs non active	Secondary	Private		Life Years Gained	$ Canadian (1997)	17754	18500
35	Russell	2001	Statin vs non active	Primary	Private		Life Years Gained	$ Canadian (1997)	17846	18596
35	Russell	2001	Statin vs non active	Primary	Private		Life Years Gained	$ Canadian (1997)	18145	18907
35	Russell	2001	Statin vs non active	Secondary	Private		Life Years Gained	$ Canadian (1997)	18217	18982
35	Russell	2001	Statin vs non active	Secondary	Private		Life Years Gained	$ Canadian (1997)	18456	19232
35	Russell	2001	Statin vs non active	Primary	Private		Life Years Gained	$ Canadian (1997)	18470	19246
35	Russell	2001	Statin vs statin	Secondary	Private		Life Years Gained	$ Canadian (1997)	18527	19306
35	Russell	2001	Statin vs statin	Secondary	Private		Life Years Gained	$ Canadian (1997)	18536	19315
35	Russell	2001	Statin vs statin	Secondary	Private		Life Years Gained	$ Canadian (1997)	18736	19523
35	Russell	2001	Statin vs non active	Primary	Private		Life Years Gained	$ Canadian (1997)	19028	19828
35	Russell	2001	Statin vs non active	Secondary	Private		Life Years Gained	$ Canadian (1997)	19186	19992
35	Russell	2001	Statin vs non active	Secondary	Private		Life Years Gained	$ Canadian (1997)	19364	20178
35	Russell	2001	Statin vs non active	Secondary	Private		Life Years Gained	$ Canadian (1997)	19501	20320
35	Russell	2001	Statin vs non active	Secondary	Private		Life Years Gained	$ Canadian (1997)	19610	20434
35	Russell	2001	Statin vs non active	Secondary	Private		Life Years Gained	$ Canadian (1997)	19762	20592

35	Russell	2001	Statin vs non active	Primary	Private	Life Years Gained	$ Canadian (1997)	19867	20702
35	Russell	2001	Statin vs non active	Secondary	Private	Life Years Gained	$ Canadian (1997)	20313	21167
35	Russell	2001	Statin vs non active	Secondary	Private	Life Years Gained	$ Canadian (1997)	20904	21782
35	Russell	2001	Statin vs non active	Secondary	Private	Life Years Gained	$ Canadian (1997)	21087	21973
35	Russell	2001	Statin vs non active	Secondary	Private	Life Years Gained	$ Canadian (1997)	21105	21992
35	Russell	2001	Statin vs non active	Secondary	Private	Life Years Gained	$ Canadian (1997)	21128	22016
35	Russell	2001	Statin vs statin	Secondary	Private	Life Years Gained	$ Canadian (1997)	21147	22036
35	Russell	2001	Statin vs non active	Secondary	Private	Life Years Gained	$ Canadian (1997)	21344	22241
35	Russell	2001	Statin vs non active	Secondary	Private	Life Years Gained	$ Canadian (1997)	21479	22382
35	Russell	2001	Statin vs non active	Secondary	Private	Life Years Gained	$ Canadian (1997)	21688	22599
35	Russell	2001	Statin vs non active	Primary	Private	Life Years Gained	$ Canadian (1997)	21778	22693
35	Russell	2001	Statin vs non active	Secondary	Private	Life Years Gained	$ Canadian (1997)	22634	23585
35	Russell	2001	Statin vs statin	Secondary	Private	Life Years Gained	$ Canadian (1997)	22771	23728
35	Russell	2001	Statin vs non active	Primary	Private	Life Years Gained	$ Canadian (1997)	22807	23765
35	Russell	2001	Statin vs non active	Primary	Private	Life Years Gained	$ Canadian (1997)	22884	23846
35	Russell	2001	Statin vs non active	Secondary	Private	Life Years Gained	$ Canadian (1997)	23179	24153
35	Russell	2001	Statin vs non active	Secondary	Private	Life Years Gained	$ Canadian (1997)	23196	24171
35	Russell	2001	Statin vs non active	Primary	Private	Life Years Gained	$ Canadian (1997)	23247	24224
35	Russell	2001	Statin vs non active	Secondary	Private	Life Years Gained	$ Canadian (1997)	23287	24266
35	Russell	2001	Statin vs non active	Primary	Private	Life Years Gained	$ Canadian (1997)	23774	24773
35	Russell	2001	Statin vs non active	Secondary	Private	Life Years Gained	$ Canadian (1997)	23823	24824
35	Russell	2001	Statin vs non active	Primary	Private	Life Years Gained	$ Canadian (1997)	24251	25270
35	Russell	2001	Statin vs statin	Secondary	Private	Life Years Gained	$ Canadian (1997)	24324	25346
35	Russell	2001	Statin vs non active	Primary	Private	Life Years Gained	$ Canadian (1997)	24448	25475
35	Russell	2001	Statin vs non active	Secondary	Private	Life Years Gained	$ Canadian (1997)	24510	25540
35	Russell	2001	Statin vs non active	Primary	Private	Life Years Gained	$ Canadian (1997)	24523	25553
35	Russell	2001	Statin vs non active	Secondary	Private	Life Years Gained	$ Canadian (1997)	24559	25591
35	Russell	2001	Statin vs non active	Primary	Private	Life Years Gained	$ Canadian (1997)	24562	25594
35	Russell	2001	Statin vs non active	Primary	Private	Life Years Gained	$ Canadian (1997)	24716	25755
35	Russell	2001	Statin vs non active	Primary	Private	Life Years Gained	$ Canadian (1997)	25065	26118
35	Russell	2001	Statin vs non active	Secondary	Private	Life Years Gained	$ Canadian (1997)	25131	26187
35	Russell	2001	Statin vs non active	Secondary	Private	Life Years Gained	$ Canadian (1997)	25183	26241
35	Russell	2001	Statin vs non active	Secondary	Private	Life Years Gained	$ Canadian (1997)	25184	26242
35	Russell	2001	Statin vs non active	Secondary	Private	Life Years Gained	$ Canadian (1997)	25687	26766
35	Russell	2001	Statin vs statin	Primary	Private	Life Years Gained	$ Canadian (1997)	25840	26926
35	Russell	2001	Statin vs non active	Primary	Private	Life Years Gained	$ Canadian (1997)	26010	27103
35	Russell	2001	Statin vs non active	Secondary	Private	Life Years Gained	$ Canadian (1997)	26440	27551
35	Russell	2001	Statin vs non active	Secondary	Private	Life Years Gained	$ Canadian (1997)	26503	27617
35	Russell	2001	Statin vs non active	Secondary	Private	Life Years Gained	$ Canadian (1997)	26552	27668
35	Russell	2001	Statin vs non active	Secondary	Private	Life Years Gained	$ Canadian (1997)	26634	27753
35	Russell	2001	Statin vs non active	Secondary	Private	Life Years Gained	$ Canadian (1997)	26977	28111
35	Russell	2001	Statin vs non active	Primary	Private	Life Years Gained	$ Canadian (1997)	27107	28246
35	Russell	2001	Statin vs non active	Secondary	Private	Life Years Gained	$ Canadian (1997)	27108	28247

35	Russell	2001	Statin vs non active	Secondary	Private		Life Years Gained	$ Canadian (1997)	27159	28300
35	Russell	2001	Statin vs non active	Secondary	Private		Life Years Gained	$ Canadian (1997)	27409	28561
35	Russell	2001	Statin vs non active	Secondary	Private		Life Years Gained	$ Canadian (1997)	27545	28702
35	Russell	2001	Statin vs non active	Secondary	Private		Life Years Gained	$ Canadian (1997)	27618	28779
35	Russell	2001	Statin vs non active	Secondary	Private		Life Years Gained	$ Canadian (1997)	27801	28969
35	Russell	2001	Statin vs non active	Secondary	Private		Life Years Gained	$ Canadian (1997)	27857	29028
35	Russell	2001	Statin vs statin	Secondary	Private		Life Years Gained	$ Canadian (1997)	27992	29168
35	Russell	2001	Statin vs non active	Secondary	Private		Life Years Gained	$ Canadian (1997)	28192	29377
35	Russell	2001	Statin vs non active	Secondary	Private		Life Years Gained	$ Canadian (1997)	28806	30016
35	Russell	2001	Statin vs non active	Secondary	Private		Life Years Gained	$ Canadian (1997)	28852	30064
35	Russell	2001	Statin vs non active	Secondary	Private		Life Years Gained	$ Canadian (1997)	29104	30327
35	Russell	2001	Statin vs non active	Secondary	Private		Life Years Gained	$ Canadian (1997)	29396	30631
35	Russell	2001	Statin vs non active	Secondary	Private		Life Years Gained	$ Canadian (1997)	29407	30643
35	Russell	2001	Statin vs statin	Primary	Private		Life Years Gained	$ Canadian (1997)	29575	30818
35	Russell	2001	Statin vs non active	Secondary	Private		Life Years Gained	$ Canadian (1997)	29801	31053
35	Russell	2001	Statin vs non active	Primary	Private		Life Years Gained	$ Canadian (1997)	30055	31318
35	Russell	2001	Statin vs non active	Secondary	Private		Life Years Gained	$ Canadian (1997)	30242	31513
35	Russell	2001	Statin vs statin	Secondary	Private		Life Years Gained	$ Canadian (1997)	30809	32104
35	Russell	2001	Statin vs non active	Secondary	Private		Life Years Gained	$ Canadian (1997)	30865	32162
35	Russell	2001	Statin vs non active	Primary	Private		Life Years Gained	$ Canadian (1997)	30998	32301
35	Russell	2001	Statin vs non active	Secondary	Private		Life Years Gained	$ Canadian (1997)	31197	32508
35	Russell	2001	Statin vs non active	Primary	Private		Life Years Gained	$ Canadian (1997)	31617	32946
35	Russell	2001	Statin vs non active	Secondary	Private		Life Years Gained	$ Canadian (1997)	31705	33037
35	Russell	2001	Statin vs non active	Secondary	Private		Life Years Gained	$ Canadian (1997)	31943	33285
35	Russell	2001	Statin vs non active	Secondary	Private		Life Years Gained	$ Canadian (1997)	31950	33293
35	Russell	2001	Statin vs non active	Primary	Private		Life Years Gained	$ Canadian (1997)	32076	33424
35	Russell	2001	Statin vs non active	Secondary	Private		Life Years Gained	$ Canadian (1997)	32442	33805
35	Russell	2001	Statin vs non active	Primary	Private		Life Years Gained	$ Canadian (1997)	33142	34535
35	Russell	2001	Statin vs non active	Secondary	Private		Life Years Gained	$ Canadian (1997)	33169	34563
35	Russell	2001	Statin vs non active	Secondary	Private		Life Years Gained	$ Canadian (1997)	33542	34951
35	Russell	2001	Statin vs non active	Primary	Private		Life Years Gained	$ Canadian (1997)	33558	34968
35	Russell	2001	Statin vs non active	Secondary	Private		Life Years Gained	$ Canadian (1997)	33658	35072
35	Russell	2001	Statin vs non active	Primary	Private		Life Years Gained	$ Canadian (1997)	33890	35314
35	Russell	2001	Statin vs non active	Secondary	Private		Life Years Gained	$ Canadian (1997)	34146	35581
35	Russell	2001	Statin vs non active	Secondary	Private		Life Years Gained	$ Canadian (1997)	34250	35689
35	Russell	2001	Statin vs non active	Primary	Private		Life Years Gained	$ Canadian (1997)	34647	36103
35	Russell	2001	Statin vs non active	Secondary	Private		Life Years Gained	$ Canadian (1997)	35540	37033
35	Russell	2001	Statin vs non active	Secondary	Private		Life Years Gained	$ Canadian (1997)	35940	37450
35	Russell	2001	Statin vs non active	Secondary	Private		Life Years Gained	$ Canadian (1997)	36015	37528
35	Russell	2001	Statin vs non active	Secondary	Private		Life Years Gained	$ Canadian (1997)	36186	37707
35	Russell	2001	Statin vs non active	Secondary	Private		Life Years Gained	$ Canadian (1997)	36635	38174
35	Russell	2001	Statin vs non active	Primary	Private		Life Years Gained	$ Canadian (1997)	36801	38347
35	Russell	2001	Statin vs non active	Primary	Private		Life Years Gained	$ Canadian (1997)	36805	38352

35	Russell	2001	Statin vs non active	Secondary	Private	Life Years Gained	$ Canadian (1997)	36824	38371
35	Russell	2001	Statin vs non active	Secondary	Private	Life Years Gained	$ Canadian (1997)	37247	38812
35	Russell	2001	Statin vs statin	Secondary	Private	Life Years Gained	$ Canadian (1997)	37299	38866
35	Russell	2001	Statin vs non active	Secondary	Private	Life Years Gained	$ Canadian (1997)	37556	39134
35	Russell	2001	Statin vs non active	Secondary	Private	Life Years Gained	$ Canadian (1997)	37996	39593
35	Russell	2001	Statin vs non active	Secondary	Private	Life Years Gained	$ Canadian (1997)	38248	39855
35	Russell	2001	Statin vs statin	Primary	Private	Life Years Gained	$ Canadian (1997)	39528	41189
35	Russell	2001	Statin vs statin	Primary	Private	Life Years Gained	$ Canadian (1997)	39545	41207
35	Russell	2001	Statin vs non active	Secondary	Private	Life Years Gained	$ Canadian (1997)	40186	41875
35	Russell	2001	Statin vs non active	Secondary	Private	Life Years Gained	$ Canadian (1997)	40445	42144
35	Russell	2001	Statin vs non active	Secondary	Private	Life Years Gained	$ Canadian (1997)	40478	42179
35	Russell	2001	Statin vs non active	Secondary	Private	Life Years Gained	$ Canadian (1997)	41269	43003
35	Russell	2001	Statin vs non active	Secondary	Private	Life Years Gained	$ Canadian (1997)	41351	43089
35	Russell	2001	Statin vs non active	Secondary	Private	Life Years Gained	$ Canadian (1997)	41603	43351
35	Russell	2001	Statin vs non active	Primary	Private	Life Years Gained	$ Canadian (1997)	41927	43689
35	Russell	2001	Statin vs statin	Secondary	Private	Life Years Gained	$ Canadian (1997)	42225	43999
35	Russell	2001	Statin vs non active	Primary	Private	Life Years Gained	$ Canadian (1997)	42334	44113
35	Russell	2001	Statin vs non active	Primary	Private	Life Years Gained	$ Canadian (1997)	42420	44202
35	Russell	2001	Statin vs non active	Secondary	Private	Life Years Gained	$ Canadian (1997)	42757	44554
35	Russell	2001	Statin vs non active	Secondary	Private	Life Years Gained	$ Canadian (1997)	43524	45353
35	Russell	2001	Statin vs statin	Primary	Private	Life Years Gained	$ Canadian (1997)	45383	47290
35	Russell	2001	Statin vs non active	Secondary	Private	Life Years Gained	$ Canadian (1997)	45460	47370
35	Russell	2001	Statin vs statin	Primary	Private	Life Years Gained	$ Canadian (1997)	45860	47787
35	Russell	2001	Statin vs non active	Secondary	Private	Life Years Gained	$ Canadian (1997)	45903	47832
35	Russell	2001	Statin vs non active	Secondary	Private	Life Years Gained	$ Canadian (1997)	45966	47897
35	Russell	2001	Statin vs non active	Secondary	Private	Life Years Gained	$ Canadian (1997)	46845	48813
35	Russell	2001	Statin vs non active	Secondary	Private	Life Years Gained	$ Canadian (1997)	46943	48916
35	Russell	2001	Statin vs non active	Secondary	Private	Life Years Gained	$ Canadian (1997)	47565	49564
35	Russell	2001	Statin vs non active	Secondary	Private	Life Years Gained	$ Canadian (1997)	47778	49786
35	Russell	2001	Statin vs non active	Primary	Private	Life Years Gained	$ Canadian (1997)	47981	49997
35	Russell	2001	Statin vs non active	Secondary	Private	Life Years Gained	$ Canadian (1997)	49300	51372
35	Russell	2001	Statin vs non active	Secondary	Private	Life Years Gained	$ Canadian (1997)	49596	51680
35	Russell	2001	Statin vs non active	Primary	Private	Life Years Gained	$ Canadian (1997)	50749	52881
35	Russell	2001	Statin vs non active	Primary	Private	Life Years Gained	$ Canadian (1997)	51036	53181
35	Russell	2001	Statin vs non active	Secondary	Private	Life Years Gained	$ Canadian (1997)	53043	55272
35	Russell	2001	Statin vs non active	Secondary	Private	Life Years Gained	$ Canadian (1997)	53413	55657
35	Russell	2001	Statin vs non active	Secondary	Private	Life Years Gained	$ Canadian (1997)	53893	56158
35	Russell	2001	Statin vs non active	Primary	Private	Life Years Gained	$ Canadian (1997)	54627	56922
35	Russell	2001	Statin vs non active	Secondary	Private	Life Years Gained	$ Canadian (1997)	55259	57581
35	Russell	2001	Statin vs non active	Primary	Private	Life Years Gained	$ Canadian (1997)	55295	57618
35	Russell	2001	Statin vs non active	Primary	Private	Life Years Gained	$ Canadian (1997)	55914	58264
35	Russell	2001	Statin vs non active	Secondary	Private	Life Years Gained	$ Canadian (1997)	56922	59314
35	Russell	2001	Statin vs non active	Secondary	Private	Life Years Gained	$ Canadian (1997)	57458	59872

35	Russell	2001	Statin vs non active	Primary	Private	Life Years Gained	$ Canadian (1997)	57620	60041
35	Russell	2001	Statin vs non active	Primary	Private	Life Years Gained	$ Canadian (1997)	57760	60187
35	Russell	2001	Statin vs non active	Secondary	Private	Life Years Gained	$ Canadian (1997)	59229	61718
35	Russell	2001	Statin vs statin	Primary	Private	Life Years Gained	$ Canadian (1997)	60509	63052
35	Russell	2001	Statin vs non active	Secondary	Private	Life Years Gained	$ Canadian (1997)	60765	63318
35	Russell	2001	Statin vs non active	Secondary	Private	Life Years Gained	$ Canadian (1997)	62301	64919
35	Russell	2001	Statin vs non active	Secondary	Private	Life Years Gained	$ Canadian (1997)	62425	65048
35	Russell	2001	Statin vs non active	Secondary	Private	Life Years Gained	$ Canadian (1997)	62547	65175
35	Russell	2001	Statin vs non active	Primary	Private	Life Years Gained	$ Canadian (1997)	63421	66086
35	Russell	2001	Statin vs non active	Primary	Private	Life Years Gained	$ Canadian (1997)	64419	67126
35	Russell	2001	Statin vs non active	Secondary	Private	Life Years Gained	$ Canadian (1997)	66742	69546
35	Russell	2001	Statin vs non active	Primary	Private	Life Years Gained	$ Canadian (1997)	67043	69860
35	Russell	2001	Statin vs non active	Secondary	Private	Life Years Gained	$ Canadian (1997)	71488	74492
35	Russell	2001	Statin vs non active	Secondary	Private	Life Years Gained	$ Canadian (1997)	71532	74538
35	Russell	2001	Statin vs non active	Primary	Private	Life Years Gained	$ Canadian (1997)	71585	74593
35	Russell	2001	Statin vs non active	Primary	Private	Life Years Gained	$ Canadian (1997)	72135	75166
35	Russell	2001	Statin vs non active	Primary	Private	Life Years Gained	$ Canadian (1997)	72271	75308
35	Russell	2001	Statin vs non active	Primary	Private	Life Years Gained	$ Canadian (1997)	74972	78122
35	Russell	2001	Statin vs non active	Secondary	Private	Life Years Gained	$ Canadian (1997)	75511	78684
35	Russell	2001	Statin vs non active	Primary	Private	Life Years Gained	$ Canadian (1997)	75603	78780
35	Russell	2001	Statin vs non active	Primary	Private	Life Years Gained	$ Canadian (1997)	76360	79569
35	Russell	2001	Statin vs non active	Primary	Private	Life Years Gained	$ Canadian (1997)	77224	80469
35	Russell	2001	Statin vs non active	Secondary	Private	Life Years Gained	$ Canadian (1997)	78601	81904
35	Russell	2001	Statin vs non active	Secondary	Private	Life Years Gained	$ Canadian (1997)	80866	84264
35	Russell	2001	Statin vs non active	Secondary	Private	Life Years Gained	$ Canadian (1997)	81236	84650
35	Russell	2001	Statin vs non active	Primary	Private	Life Years Gained	$ Canadian (1997)	83226	86723
35	Russell	2001	Statin vs non active	Primary	Private	Life Years Gained	$ Canadian (1997)	84101	87635
35	Russell	2001	Statin vs non active	Secondary	Private	Life Years Gained	$ Canadian (1997)	85396	88984
35	Russell	2001	Statin vs non active	Primary	Private	Life Years Gained	$ Canadian (1997)	86905	90557
35	Russell	2001	Statin vs non active	Primary	Private	Life Years Gained	$ Canadian (1997)	87906	91600
35	Russell	2001	Statin vs non active	Primary	Private	Life Years Gained	$ Canadian (1997)	88077	91778
35	Russell	2001	Statin vs non active	Secondary	Private	Life Years Gained	$ Canadian (1997)	91564	95412
35	Russell	2001	Statin vs non active	Primary	Private	Life Years Gained	$ Canadian (1997)	91673	95525
35	Russell	2001	Statin vs non active	Primary	Private	Life Years Gained	$ Canadian (1997)	94001	97951
35	Russell	2001	Statin vs non active	Secondary	Private	Life Years Gained	$ Canadian (1997)	94041	97993
35	Russell	2001	Statin vs non active	Primary	Private	Life Years Gained	$ Canadian (1997)	94732	98713
35	Russell	2001	Statin vs non active	Primary	Private	Life Years Gained	$ Canadian (1997)	98291	102421
35	Russell	2001	Statin vs non active	Secondary	Private	Life Years Gained	$ Canadian (1997)	106556	111033
35	Russell	2001	Statin vs non active	Primary	Private	Life Years Gained	$ Canadian (1997)	108154	112699
35	Russell	2001	Statin vs non active	Primary	Private	Life Years Gained	$ Canadian (1997)	109034	113616
35	Russell	2001	Statin vs non active	Primary	Private	Life Years Gained	$ Canadian (1997)	113601	118375
35	Russell	2001	Statin vs non active	Primary	Private	Life Years Gained	$ Canadian (1997)	114128	118924
35	Russell	2001	Statin vs non active	Primary	Private	Life Years Gained	$ Canadian (1997)	114614	119430

35	Russell	2001	Statin vs non active	Primary	Private	Life Years Gained	$ Canadian (1997)	141109	147038
36	Glasziou	2002	Statin vs non active	Secondary	Private	Life Years Gained	$ Australian (1998)	10938	11742
37	Barry	2002	Statin vs non active	Secondary	None	Quality-Adjusted Life Years	Euro (2001)	1172	1666
37	Barry	2002	Statin vs non active	Secondary	None	Quality-Adjusted Life Years	Euro (2001)	2358	3352
37	Barry	2002	Statin vs non active	Secondary	None	Quality-Adjusted Life Years	Euro (2001)	2358	3352
37	Barry	2002	Statin vs non active	Secondary	None	Quality-Adjusted Life Years	Euro (2001)	2788	3963
38	Scuffham	2004	Statin vs non active	Secondary	Private	Quality-Adjusted Life Years	£ UK (2002)	4528	7307
39	Pilote	2005	Statin vs non active	Secondary	Public or non-profit	Life Years Gained	$ Canadian (1996)	10588	11033
39	Pilote	2005	Statin vs non active	Secondary	Public or non-profit	Life Years Gained	$ Canadian (1996)	11903	12403
39	Pilote	2005	Statin vs non active	Secondary	Public or non-profit	Life Years Gained	$ Canadian (1996)	12572	13100
39	Pilote	2005	Statin vs non active	Secondary	Public or non-profit	Life Years Gained	$ Canadian (1996)	13713	14289
39	Pilote	2005	Statin vs non active	Secondary	Public or non-profit	Life Years Gained	$ Canadian (1996)	16122	16799
39	Pilote	2005	Statin vs non active	Secondary	Public or non-profit	Life Years Gained	$ Canadian (1996)	20686	21555
39	Pilote	2005	Statin vs non active	Secondary	Public or non-profit	Life Years Gained	$ Canadian (1996)	21156	22045
39	Pilote	2005	Statin vs non active	Secondary	Public or non-profit	Life Years Gained	$ Canadian (1996)	22314	23252
39	Pilote	2005	Statin vs non active	Primary	Public or non-profit	Life Years Gained	$ Canadian (1996)	22674	23627
39	Pilote	2005	Statin vs non active	Secondary	Public or non-profit	Life Years Gained	$ Canadian (1996)	24631	25666
39	Pilote	2005	Statin vs non active	Secondary	Public or non-profit	Life Years Gained	$ Canadian (1996)	29367	30601
39	Pilote	2005	Statin vs non active	Primary	Public or non-profit	Life Years Gained	$ Canadian (1996)	30676	31965
39	Pilote	2005	Statin vs non active	Primary	Public or non-profit	Life Years Gained	$ Canadian (1996)	33786	35206
39	Pilote	2005	Statin vs non active	Secondary	Public or non-profit	Life Years Gained	$ Canadian (1996)	37394	38965
39	Pilote	2005	Statin vs non active	Primary	Public or non-profit	Life Years Gained	$ Canadian (1996)	37999	39596
39	Pilote	2005	Statin vs non active	Primary	Public or non-profit	Life Years Gained	$ Canadian (1996)	40330	42025
39	Pilote	2005	Statin vs non active	Primary	Public or non-profit	Life Years Gained	$ Canadian (1996)	55755	58098
39	Pilote	2005	Statin vs non active	Secondary	Public or non-profit	Life Years Gained	$ Canadian (1996)	104984	109395
39	Pilote	2005	Statin vs non active	Primary	Public or non-profit	Life Years Gained	$ Canadian (1996)	119250	124261
39	Pilote	2005	Statin vs non active	Primary	Public or non-profit	Life Years Gained	$ Canadian (1996)	124318	129542
39	Pilote	2005	Statin vs non active	Primary	Public or non-profit	Life Years Gained	$ Canadian (1996)	162258	169076
39	Pilote	2005	Statin vs non active	Primary	Public or non-profit	Life Years Gained	$ Canadian (1996)	186811	194661
39	Pilote	2005	Statin vs non active	Primary	Public or non-profit	Life Years Gained	$ Canadian (1996)	214484	223497
39	Pilote	2005	Statin vs non active	Primary	Public or non-profit	Life Years Gained	$ Canadian (1996)	413897	431289
40	Delea	2005	Statin vs non active	Secondary	Private	Quality-Adjusted Life Years	$ USA (2002)	15471	15471
41	Nagata-Kobayash	2005	Statin vs non active	Primary	None	Quality-Adjusted Life Years	Yen (2002)	44000000	532400
41	Nagata-Kobayash	2005	Statin vs non active	Primary	None	Quality-Adjusted Life Years	Yen (2002)	76000000	919600
42	Scuffham	2005	Statin vs non active	Secondary	Private	Quality-Adjusted Life Years	£ UK (2002)	51	82
43	Heart Protection	2006	Statin vs non active	Secondary	Private	Life Years Gained	$ UK (2001)	2610	4212
43	Heart Protection	2006	Statin vs non active	Secondary	Private	Life Years Gained	$ UK (2001)	2860	4615
43	Heart Protection	2006	Statin vs non active	Secondary	Private	Life Years Gained	$ UK (2001)	3040	4905
43	Heart Protection	2006	Statin vs non active	Secondary	Private	Life Years Gained	$ UK (2001)	3340	5390
43	Heart Protection	2006	Statin vs non active	Secondary	Private	Life Years Gained	$ UK (2001)	3620	5841
43	Heart Protection	2006	Statin vs non active	Secondary	Private	Life Years Gained	$ UK (2001)	3870	6245
43	Heart Protection	2006	Statin vs non active	Secondary	Private	Life Years Gained	$ UK (2001)	4190	6761
43	Heart Protection	2006	Statin vs non active	Secondary	Private	Life Years Gained	$ UK (2001)	4610	7439

43	Heart Protection	2006	Statin vs non active	Secondary	Private	Life Years Gained	$ UK (2001)	4710	7600
43	Heart Protection	2006	Statin vs non active	Secondary	Private	Life Years Gained	$ UK (2001)	4900	7907
43	Heart Protection	2006	Statin vs non active	Secondary	Private	Life Years Gained	$ UK (2001)	5230	8439
43	Heart Protection	2006	Statin vs non active	Secondary	Private	Life Years Gained	$ UK (2001)	5740	9262
43	Heart Protection	2006	Statin vs non active	Secondary	Private	Life Years Gained	$ UK (2001)	6010	9698
43	Heart Protection	2006	Statin vs non active	Secondary	Private	Life Years Gained	$ UK (2001)	6170	9956
43	Heart Protection	2006	Statin vs non active	Secondary	Private	Life Years Gained	$ UK (2001)	6430	10376
43	Heart Protection	2006	Statin vs non active	Secondary	Private	Life Years Gained	$ UK (2001)	6910	11150
43	Heart Protection	2006	Statin vs non active	Secondary	Private	Life Years Gained	$ UK (2001)	8470	13668
43	Heart Protection	2006	Statin vs non active	Secondary	Private	Life Years Gained	$ UK (2001)	8720	14071
43	Heart Protection	2006	Statin vs non active	Secondary	Private	Life Years Gained	$ UK (2001)	8910	14378
43	Heart Protection	2006	Statin vs non active	Secondary	Private	Life Years Gained	$ UK (2001)	9260	14942
44	Tonkin	2006	Statin vs non active	Secondary	Public or non-profit	Life Years Gained	$ Australian (1998)	7581	8138
44	Tonkin	2006	Statin vs non active	Secondary	Public or non-profit	Life Years Gained	$ Australian (1998)	14944	16043
45	Kohli	2006	Statin vs statin	Primary	Private	Quality-Adjusted Life Years	$ Canadian (2002)	25344	26409
45	Kohli	2006	Statin vs statin	Primary	Private	Quality-Adjusted Life Years	$ Canadian (2002)	25445	26514
45	Kohli	2006	Statin vs statin	Secondary	Private	Quality-Adjusted Life Years	$ Canadian (2002)	27442	28595
45	Kohli	2006	Statin vs statin	Secondary	Private	Quality-Adjusted Life Years	$ Canadian (2002)	28920	30135
46	Fernández de Bol	2006	Statin vs non active	Primary	Private	Quality-Adjusted Life Years	Euro(2004)	8046	11437
47	Walshe	2006	Statin vs statin	Primary	None	Life Years Gained	£ UK (2005)	24500	39534
47	Walshe	2006	Statin vs statin	Primary	None	Life Years Gained	£ UK (2005)	25500	41148
47	Walshe	2006	Statin vs statin	Primary	None	Life Years Gained	£ UK (2005)	25800	41632
47	Walshe	2006	Statin vs statin	Primary	None	Life Years Gained	£ UK (2005)	29999	48408
47	Walshe	2006	Statin vs statin	Primary	None	Life Years Gained	£ UK (2005)	38999	62930
48	Lindgren	2007	Statin vs statin	Secondary	Private	Quality-Adjusted Life Years	Euro(2005)	35210	50047
48	Lindgren	2007	Statin vs statin	Secondary	Private	Quality-Adjusted Life Years	Euro(2005)	43667	62068
48	Lindgren	2007	Statin vs statin	Secondary	Private	Quality-Adjusted Life Years	Euro (2005)	47197	67086
48	Lindgren	2007	Statin vs statin	Secondary	Private	Quality-Adjusted Life Years	Euro(2005)	62639	89035
50	Raikou	2007	Statin vs non active	Primary	Private	Quality-Adjusted Life Years	£ UK (2004)	6471	10442
51	Lafuma	2008	Statin vs non active	Primary	Private	Life Years Gained	Euro(2007)	1418	2016
52	Peura	2008	Statin vs statin	Primary	Private	Quality-Adjusted Life Years	Euro (2006)	0	0
52	Peura	2008	Statin vs statin	Secondary	Private	Quality-Adjusted Life Years	Euro (2006)	0	0
52	Peura	2008	Statin vs statin	Primary	Private	Quality-Adjusted Life Years	Euro (2006)	36548	51949
52	Peura	2008	Statin vs statin	Secondary	Private	Quality-Adjusted Life Years	Euro (2006)	36548	51949
52	Peura	2008	Statin vs statin	Primary	Private	Quality-Adjusted Life Years	Euro (2006)	105599	150098
52	Peura	2008	Statin vs statin	Secondary	Private	Quality-Adjusted Life Years	Euro (2006)	105599	150098
53	Pinto	2008	Statin vs statin	Primary	Private	Quality-Adjusted Life Years	Euro (2007)	0	0
53	Pinto	2008	Statin vs statin	Primary	Private	Quality-Adjusted Life Years	Euro (2007)	30350	43139
53	Pinto	2008	Statin vs statin	Primary	Private	Quality-Adjusted Life Years	Euro (2007)	39340	55918
54	Ramsey	2008	Statin vs non active	Primary	Private	Quality-Adjusted Life Years	$ USA (2005)	0	0
54	Ramsey	2008	Statin vs non active	Primary	Private	Quality-Adjusted Life Years	$ USA (2005)	3640	3640
54	Ramsey	2008	Statin vs non active	Primary	Private	Quality-Adjusted Life Years	$ USA (2005)	137276	137276
55	Newman	2008	Statin vs non active	Primary	None	Quality-Adjusted Life Years	$ USA (2004)	7176	7176

56	Alonso	2008	Statin vs non active	Primary	Private	Life Years Gained	Euro (2005)	1821	2588
56	Alonso	2008	Statin vs non active	Primary	Private	Life Years Gained	Euro (2005)	3012	4281
56	Alonso	2008	Statin vs non active	Primary	Private	Life Years Gained	Euro (2005)	4021	5715
56	Alonso	2008	Statin vs non active	Primary	Private	Life Years Gained	Euro (2005)	5250	7462
57	Heart Protection	2009	Statin vs non active	Secondary	Private	Life Years Gained	$ USA (2006)	2500	2500
57	Heart Protection	2009	Statin vs non active	Secondary	Private	Life Years Gained	$ USA (2006)	3690	3690
57	Heart Protection	2009	Statin vs non active	Secondary	Private	Life Years Gained	$ USA (2006)	4070	4070
57	Heart Protection	2009	Statin vs non active	Secondary	Private	Life Years Gained	$ USA (2006)	4880	4880
57	Heart Protection	2009	Statin vs non active	Secondary	Private	Life Years Gained	$ USA (2006)	5150	5150
57	Heart Protection	2009	Statin vs non active	Secondary	Private	Life Years Gained	$ USA (2006)	5570	5570
57	Heart Protection	2009	Statin vs non active	Secondary	Private	Life Years Gained	$ USA (2006)	6140	6140
57	Heart Protection	2009	Statin vs non active	Secondary	Private	Life Years Gained	$ USA (2006)	6210	6210
57	Heart Protection	2009	Statin vs non active	Secondary	Private	Life Years Gained	$ USA (2006)	6730	6730
57	Heart Protection	2009	Statin vs non active	Secondary	Private	Life Years Gained	$ USA (2006)	6940	6940
57	Heart Protection	2009	Statin vs non active	Secondary	Private	Life Years Gained	$ USA (2006)	7320	7320
57	Heart Protection	2009	Statin vs non active	Secondary	Private	Life Years Gained	$ USA (2006)	7620	7620
57	Heart Protection	2009	Statin vs non active	Secondary	Private	Life Years Gained	$ USA (2006)	8030	8030
57	Heart Protection	2009	Statin vs non active	Secondary	Private	Life Years Gained	$ USA (2006)	8570	8570
57	Heart Protection	2009	Statin vs non active	Secondary	Private	Life Years Gained	$ USA (2006)	8870	8870
57	Heart Protection	2009	Statin vs non active	Secondary	Private	Life Years Gained	$ USA (2006)	9380	9380
57	Heart Protection	2009	Statin vs non active	Secondary	Private	Life Years Gained	$ USA (2006)	9570	9570
57	Heart Protection	2009	Statin vs non active	Secondary	Private	Life Years Gained	$ USA (2006)	9650	9650
57	Heart Protection	2009	Statin vs non active	Secondary	Private	Life Years Gained	$ USA (2006)	10130	10130
57	Heart Protection	2009	Statin vs non active	Secondary	Private	Life Years Gained	$ USA (2006)	10990	10990
58	Kongnakorn	2009	Statin vs non active	Secondary	Private	Quality-Adjusted Life Years	$ USA (2005)	13916	13916
59	Ara	2009	Statin vs statin	Secondary	Public or non-profit	Quality-Adjusted Life Years	£ UK (2008)	12484	20145
59	Ara	2009	Statin vs statin	Secondary	Public or non-profit	Quality-Adjusted Life Years	£ UK (2008)	17469	28189
60	Taylor	2009	Statin vs statin	Secondary	Private	Quality-Adjusted Life Years	Euro (2005)	9500	13503
60	Taylor	2009	Statin vs statin	Secondary	Private	Quality-Adjusted Life Years	Euro (2005)	15000	21321
60	Taylor	2009	Statin vs statin	Secondary	Private	Quality-Adjusted Life Years	Euro (2005)	21000	29849
61	Wagner	2009	Statin vs statin	Secondary	Private	Quality-Adjusted Life Years	$ Canadian (2007)	11969	12472
62	Wagner	2009	Statin vs statin	Secondary	Private	Quality-Adjusted Life Years	$ Canadian (2007)	26795	27921
63	Lindgren	2009	Statin vs non active	Primary	Private	Quality-Adjusted Life Years	Euro(2007)	8591	12211
63	Lindgren	2009	Statin vs non active	Primary	Private	Quality-Adjusted Life Years	Euro(2007)	11965	17007
64	Annemans	2010	Statin vs non active	Primary	Private	Quality-Adjusted Life Years	Euro (2009)	16681	23710
65	Ohsfeldt	2010	Statin vs non active	Primary	Private	Quality-Adjusted Life Years	$ USA (2009)	7062	7062
66	Soini	2010	Statin vs statin	Secondary	Private	Quality-Adjusted Life Years	Euro (2007)	15335	21797
66	Soini	2010	Statin vs statin	Secondary	Private	Quality-Adjusted Life Years	Euro (2007)	17636	25068
66	Soini	2010	Statin vs statin	Secondary	Private	Quality-Adjusted Life Years	Euro (2007)	19337	27486
66	Soini	2010	Statin vs statin	Secondary	Private	Quality-Adjusted Life Years	Euro (2007)	19738	28056
66	Soini	2010	Statin vs statin	Secondary	Private	Quality-Adjusted Life Years	Euro (2007)	21405	30425
66	Soini	2010	Statin vs statin	Secondary	Private	Quality-Adjusted Life Years	Euro (2007)	22841	32466
66	Soini	2010	Statin vs statin	Secondary	Private	Quality-Adjusted Life Years	Euro (2007)	23596	33539

66	Soini	2010	Statin vs statin	Secondary	Private	Quality-Adjusted Life Years	Euro (2007)	24017	34138
66	Soini	2010	Statin vs statin	Secondary	Private	Quality-Adjusted Life Years	Euro (2007)	26595	37802
66	Soini	2010	Statin vs statin	Secondary	Private	Quality-Adjusted Life Years	Euro (2007)	30810	43793
66	Soini	2010	Statin vs statin	Secondary	Private	Quality-Adjusted Life Years	Euro (2007)	40087	56980
66	Soini	2010	Statin vs statin	Secondary	Private	Quality-Adjusted Life Years	Euro (2007)	46686	66359
67	Slejko	2010	Statin vs non active	Primary	None	Quality-Adjusted Life Years	$ US (2009)	10889	10889
68	Nherera	2010	Statin vs statin	Primary	Public or non-profit	Quality-Adjusted Life Years	£ UK (2008)	0	0
68	Nherera	2010	Statin vs statin	Primary	Public or non-profit	Quality-Adjusted Life Years	£ UK (2008)	0	0
68	Nherera	2010	Statin vs statin	Primary	Public or non-profit	Quality-Adjusted Life Years	£ UK (2008)	3035	4897
69	MacDonald	2010	Statin vs non active	Primary	None	Quality-Adjusted Life Years	$ USA (2009)	35455	35455
69	MacDonald	2010	Statin vs non active	Primary	None	Quality-Adjusted Life Years	$ USA (2009)	90714	90714
70	Reckless	2010	Statin vs statin	Secondary	Private	Quality-Adjusted Life Years	£ UK (2008)	11571	18671
71	Rosen	2010	Statin vs statin	Secondary	Private	Quality-Adjusted Life Years	$ USA (2007)	13600	13600
72	Arrospide	2011	Statin vs non active	Secondary	Private	Quality-Adjusted Life Years	Euro(2009)	9919	14099
73	Ara	2011	Statin vs statin	Secondary	Public or non-profit	Quality-Adjusted Life Years	£ UK (2008)	0	0
73	Ara	2011	Statin vs statin	Secondary	Public or non-profit	Quality-Adjusted Life Years	£ UK (2008)	16344	26373
73	Ara	2011	Statin vs statin	Secondary	Public or non-profit	Quality-Adjusted Life Years	£ UK (2008)	19710	31805
74	Choudhry	2011	Statin vs non active	Primary	Private	Quality-Adjusted Life Years	$ USA (2009)	25198	25198
75	Michailov	2011	Statin vs statin	Secondary	Private	Life Years Gained	Euro (2009)	10342	14700
75	Michailov	2011	Statin vs statin	Secondary	Private	Life Years Gained	Euro (2009)	14081	20015
75	Michailov	2011	Statin vs statin	Secondary	Private	Life Years Gained	Euro (2009)	15579	22144
75	Michailov	2011	Statin vs statin	Secondary	Private	Life Years Gained	Euro (2009)	20462	29085
